# Vaccination and the Risk of Childhood Cancer—A Systematic Review and Meta-Analysis

**DOI:** 10.3389/fonc.2020.610843

**Published:** 2021-01-22

**Authors:** Manuela Marron, Lara Kim Brackmann, Pia Kuhse, Lara Christianson, Ingo Langner, Ulrike Haug, Wolfgang Ahrens

**Affiliations:** ^1^ Leibniz Institute for Prevention Research and Epidemiology—BIPS, Department Epidemiological Methods and Etiological Research, Bremen, Germany; ^2^ Leibniz Institute for Prevention Research and Epidemiology—BIPS, Library, Bremen, Germany; ^3^ Leibniz Institute for Prevention Research and Epidemiology—BIPS, Department Clinical Epidemiology, Bremen, Germany; ^4^ University of Bremen, Faculty of Human and Health Sciences, Bremen, Germany; ^5^ University of Bremen, Faculty of Mathematics and Computer Science, Bremen, Germany

**Keywords:** acute lymphoblastic leukemia, childhood leukemia, leukemia death, immunization, immune system

## Abstract

**Introduction:**

Infections may play a role in the etiology of childhood cancer and immunizations may be protective because vaccinations stimulate the immune system. Observational studies reported inconsistent associations between vaccination and risk of childhood cancer. Since a synthesis of the evidence is lacking, we conducted a meta-analysis stratified by histological and site-specific cancer.

**Methods:**

We performed a systematic review (CRD42020148579) following PRISMA guidelines and searched for literature in MEDLINE, Embase, and the Science Citation Index databases. We identified in three literature databases 7,594 different articles of which 35 met the inclusion criteria allowing for 27 analyses of 11 cancer outcomes after exposure to nine different types of vaccinations. We calculated summary odds ratios (ORs) and 95% confidence intervals (CIs) using random effects models.

**Results:**

We observed four inverse associations between childhood leukemia and certain vaccines as well as the number of vaccinations: OR 0.49 (95% CI = 0.32 to 0.74) for leukemia death after bacillus Calmette–Guérin vaccination; OR 0.76 (95% CI = 0.65 to 0.90) for acute lymphoblastic leukemia after Haemophilus influenzae type b vaccination; OR 0.57 (95% CI = 0.36 to 0.88) for leukemia; and OR 0.62 (95% CI = 0.46 to 0.85) for acute lymphoblastic leukemia after three or more vaccinations of any type. All other conducted analyses did not show any associations.

**Discussion:**

The results are consistent with the hypothesis that vaccinations reduce the risk of childhood leukemia. However, the robustness and validity of these results is limited due to the small number, substantial heterogeneity, and methodological limitations of available studies.

## Introduction

Childhood cancers include a broad spectrum of histological and site-specific cancers occurring before 18 years of age (1–3). An estimated 10% of childhood cancers can be traced back to specific rare genetic syndromes with a high cancer risk (4, 5) or a common genetic susceptibility with a small increased risk for childhood cancer (6–11). The only established environmental risk factors are high doses of ionizing radiation (3) and certain chemicals such as benzene for leukemia (12) and for acute myeloid leukemia (AML) cytostatic drugs (5). However, the evidence to date does not suggest that environmental risk factors alone can explain the majority of childhood cancers. Indeed gene-environment interactions of several pre- and postnatal factors are assumed to be involved in their etiology (3, 13).

The peaks in the incidence of acute leukemia in children aged 2 to 5 years that parallel the peaks in infection rates supports the hypothesis that immunological risk factors are involved in the etiology of leukemia (14–16). However, no single infectious agent has been identified as a risk factor for the development of leukemia to date (2, 17). Instead, Kinlen (18–20) proposed the “population mixing” hypothesis with an increased risk for leukemia and non-Hodgkin lymphoma in isolated areas due to lower herd immunity to infections. In addition, Greaves (21) suggested the “delayed infection” hypothesis with a higher risk of acute lymphoblastic leukemia (ALL) in children who did not experience any strengthening of the immune system by an acquired infection in their first year of life. This theory corresponds to the current state of science according to which the immune system plays an important role in the development of cancer (22). For acute leukemia, the possible protective role of immunization is based on the assumption that vaccines also stimulate a better performance of the immune system by formation of antibodies (23). Moreover, it has been suggested that vaccination regulates the risk of childhood cancer in general by non-specific stimulation of certain macrophages and natural killer cells that target tumors (24). This non-specific effects of vaccines may be related to cross-reactivity of the adaptive immune system with unrelated pathogens and to training of the innate immune system through epigenetic reprogramming (25). However, the beneficial effect may be limited to specific vaccines e.g. live vaccines, may be reversed with other vaccines and thus may depend on the sequence of different vaccinations (25). In line with these theories, epidemiological studies investigated the relationship between various factors (26–31) that could stimulate the immune system such as vaccinations and the occurrence of leukemia (32) and other childhood cancers (3, 33).

To summarize evidence on the association between vaccination and childhood cancer, only two meta-analyses have been conducted so far. One focusing on poliomyelitis vaccines, simian virus 40 and human cancer was published in 2004 (34) and another one focusing on early vaccination and childhood leukemia was published in 2017 (32). However, to our knowledge, there is no meta-analysis on different types of vaccinations and the risk of childhood cancer in general or on histological and site-specific subtypes other than leukemia. We aimed to fill this gap and conducted a systematic literature search and meta-analysis of the association between different types of vaccination and the risk of childhood cancer including stratification by cancer sites.

## Materials and Methods

Following the meta-analysis of observational studies in epidemiology (MOOSE) guidelines (35) and the preferred reporting items for systematic reviews and meta-analyses (PRISMA) (36), we conducted a comprehensive review of the literature to identify all available risk estimates on the association between vaccination and childhood cancer (PROSPERO registration: CRD42020148579).

### Search Strategy

**Figure 1:**
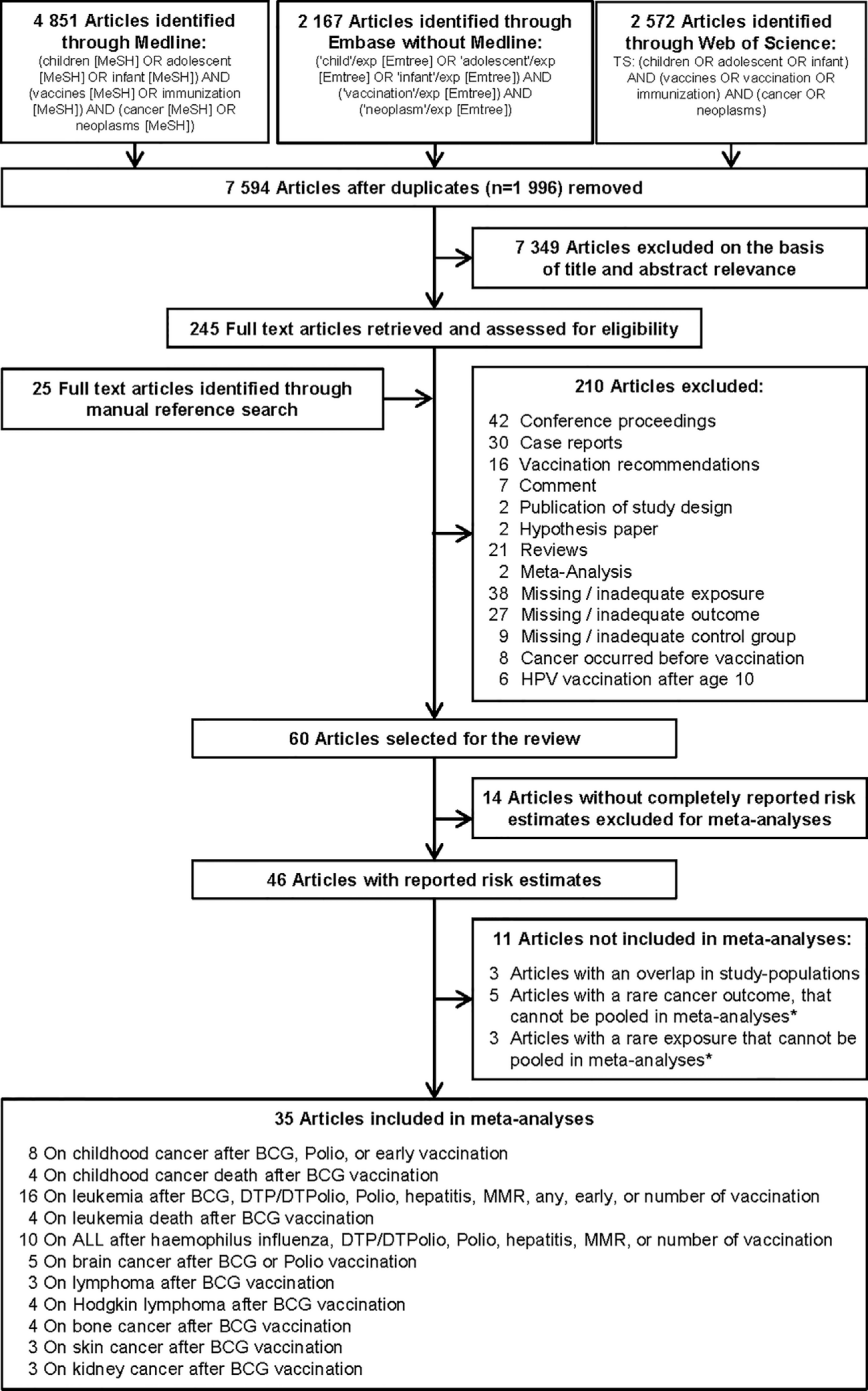
Selection of studies included in the systematic review and meta-analysis on vaccination and risk of childhood cancer. * Studies could not be pooled in the meta-analysis due to an insufficient number of estimates (only one or two) on the same association. ALL, acute lymphoblastic leukemia; BCG, Bacillus Calmette-Guérin; DTP/DTPolio, diphtheria-tetanus-pertussis/-poliomyelitis; MMR, mumps-measles-rubella; Polio, poliomyelitis.

To identify studies on the association between vaccination and childhood cancer, we systematically searched the literature databases MEDLINE, Embase, and the Science Citation Index for relevant articles published before November 2020. We used subject headings and keywords in English depending on the search structure of the literature database to combine the references related to the population, the exposure, and the disease. The search was not restricted by language filters and no date limits or other filters were used. A detailed description of the search strategy is provided in **Figure 1**. Included articles were also manually searched for potentially relevant citations not detected by the electronic search.

### Study Selection

Duplicates found by the three literature searches were deleted using EndNote. Two independent researchers performed the screening of titles and abstracts for relevant publications and conducted the full-text review of selected articles. Studies were considered for inclusion in the review if they met the following three criteria: They were 1) an original epidemiological study that examined the influence of vaccination on the risk of childhood cancer or cancer death; 2) a proper reference group without cancer or without the same cancer as the investigated outcome; and 3) the recommended first application of the studied vaccine should be before age 10 (e.g. exclusion of human papillomaviruses vaccination). Detailed exclusion criteria are shown in **Figure 1**. For inclusion in the meta-analysis, studies had to report quantitative risk estimates [odds ratio (OR), relative risk (RR), or hazard ratio (HR)], and their variability [variance, standard error (SE), standard deviation (SD), or confidence interval (CI)], or provide the numbers of cases and controls so that crude risk estimates with CI could be calculated. When multiple articles reported on the same population, the most recent article or the most informative publication was included. Since most studies used non-vaccinated children as a reference, we excluded four studies that compared vaccinated children in one time period with vaccinated children in another time period (33, 37–39). Disagreements between the two reviewers were resolved by discussion with a third reviewer.

### Data Extraction

Two reviewers extracted a predetermined set of data for each risk estimate on vaccinations and childhood cancer independently from each publication (**Supplementary Tables 1** and **2A–C**). The extracted data included the following information: name of the first author, year of publication, study location, study period, study design, number of cases (cancers or cancer deaths), number, and type of subjects in the reference group (e.g. population-based or hospital-based), assessment of the outcome, cancer site, age range at diagnosis or death, exposure assessment, vaccine, age at vaccination, matching factors, adjustment variables, reason for exclusion from meta-analysis (no or incomplete risk estimates, i.e. a RR without the 95% CI, without the *P* value for measures of association and without the number of cases and controls, overlap with other study, less than three studies on an outcome or an exposure), statistical model, risk estimate [OR, RR, HR, standard incidence ratio (SIR) along with SE, SD, 95% CI, *P* value for measures of association], and number of subjects per group. A third reviewer adjudicated inconsistencies between the two original reviewers.

### Quality Assessment

The quality of each study was assessed by applying the Newcastle-Ottawa Scale (NOS) (40), which has been widely used as recommended by the Cochrane Collaboration (41). The NOS comprises nine items categorized into three sections. Depending on the study design, NOS items differ. For case-control studies, the quality was assessed by case definition and representativeness, selection and definition of controls, comparability between cases and controls, ascertainment of exposure, same method of ascertainment for cases and controls, and response proportion. The cohort studies were evaluated for representativeness of the exposure cohort, selection of the non-exposed cohort, ascertainment of exposure, demonstration that outcome of interest was not present at start of study, comparability of cohorts on the basis of the design or analysis, validity of the outcome assessment, duration of follow-up long enough for outcomes to occur, and adequacy of the follow-up cohort (complete follow-up or follow-up >50%). Since the NOS is very general (42) and no other tool for quality assessment is established for observational studies (43), we applied a self-developed scoring system in addition. This score (maximum 45 points) was composed of the following eight criteria, each of which consists of 4 to 15 items: study design (up to six points), study size (up to six points), outcome assessment (up to six points), exposure assessment (up to six points), controlling for confounders (up to six points), statistical methods (up to six points), other methods (up to six points), and reported important characteristics of the study population (up to three points). At the end of the evaluation, there was the possibility to decrease the score by up to six points for selection problems, confounding, bias, and other limitations not described in the eight items before. Quality criteria are detailed in **Supplementary Tables 3A, B**. Two reviewers extracted the quality items of the included studies independently, discussed the results, and solved disagreements with a third investigator.

### Data Analysis

Pooled ORs with 95% CI were calculated using random effects models if three or more studies on a specific research question were available (44). To assess the association of cancer with increasing number of vaccinations (dose-response), trend analyses were conducted based on the method of Greenland and Longnecker (45) where possible. To conduct the trend analyses required for these analyses, at least two estimates with different numbers of vaccination and the same cancer outcome had to be available. The I² statistic was calculated to quantify between-study heterogeneity. We considered values of 50% or less, more than 50 to 75%, and more than 75% to indicate low, moderate, and substantial heterogeneity, respectively (46). Statistical significance of I^2^ was analyzed with the Q statistic [*P* value for heterogeneity (*P*)]. We explored whether heterogeneity could be reduced by omitting each study in turn from the meta-analysis (47). Potential sources of heterogeneity were investigated by conducting subgroup analyses by time period before and after contamination of the poliomyelitis vaccine by carcinogenic simian virus 40 (<1964; 1964+), study design (case-control or ecological study; cohort or case-cohort study), quality of exposure assessment (low: self-report or vaccination card; high: trial, registry, or medical documentation), quality of confounder control (low: basic or no adjustment/matching; high: adjustment or matching for other vaccines or immunological factors), consideration of a latency period (no; yes), Newcastle-Ottawa Scale below and above the fourth quintile (low: quality scale <6; high: quality scale 6+), quality score below and above the fourth quintile (low: detailed quality score <24.7; high: detailed quality score 24.7+), and assessment of outcome *via* registries (yes: *via* registries; no: other sources). For analyses with five or more included studies, publication bias was evaluated using funnel plots and the tests described by Egger et al. (48). All *P* values are two-sided. All calculations were performed using STATA version 14 (StataCorp LP, College Station, TX, USA) (49) or Excel version 2013 (Microsoft Cooperation, Redmond, WA, USA).

## Results

### Literature Search

Of the 9,590 identified articles, 1,996 were duplicate search results from the three literature databases and 7,349 articles were excluded on the basis of title and abstract screening, leaving 245 articles for full-text evaluation (**Figure 1**). We identified additional 25 potentially relevant articles by evaluating cross-references. Finally, 210 full-text articles were discarded according to the exclusion criteria, leaving 60 studies (50–109) in the systematic review (**Supplementary Table 1**). These studies reported 709 risk estimates for different childhood cancers after diverse types of vaccination (**Supplementary Tables 2A–C**). Overall, 85 effect estimates showed a decreased risk of different childhood cancers, 48 showed an increased risk, and 576 revealed no significant association. For the analyses, 25 of the 60 studies were excluded because (a) they reported no or incomplete risk estimates [number of studies (N) = 14], (b) the study sample overlapped with another included study (N = 3), or (c) the outcome (N = 5) or specific type of vaccination (N = 3) was reported by less than three studies. This left 35 remaining studies for inclusion in 27 specific analyses on 11 different childhood cancer outcomes after exposure to nine different types of vaccination (**Figure 1**).

### Study Characteristics and Quality

Characteristics of the 35 studies included in the meta-analysis are provided in **Tables 1** **–** **4**. Studies were published between 1968 and 2019 and covered a study period of 65 years (1943 to 2008). They were conducted in Europe (57%), North America (26%), South America (8%), Australia or New Zealand (6%), and Asia (3%) with sample sizes ranging from 148 to 1,224,914 participants. Most studies examined only children under 18 years of age, with some exceptions (60, 63, 64, 66, 73, 74, 77, 79, 98, 99, 104). The distribution of selected quality-related factors is summarized in **Supplementary Tables 3A, B**. The minority of the studies included in the meta-analysis were retrospective cohort or case cohort studies (31%), the majority were case-control studies (69%), of which 18 included population-based (63, 65, 70, 74, 76, 81, 82, 92–95, 97–101, 103, 109) and four hospital-based controls (53, 83, 86, 91). There was only one study with an ecological design that used aggregated data (105). Most studies used laboratory, trial, accounting, registry, or medical documentation to assess the outcome (86%). Only three studies (62, 66, 76) used death certificates, and two studies (77, 94) did not report on this issue. To assess the type and date of vaccination, 40% of the studies used trial, accounting, registry, or medical data, and 40% used parental reports (74, 76, 81–83, 86, 91, 92, 94, 95, 97–99, 109). Further 17% used vaccination cards (63, 93, 100, 101, 103, 104) and 3% used aggregated, external data (105). The majority of studies controlled only for basic confounders (57%), mainly age and sex, while 20% did not take any confounding into account. Ten studies (29%) accounted for a latency period of at least 1 month between the vaccination and the onset of the childhood cancer and verified by this the correct temporal sequence of exposure and outcome. None of the studies reported on the inclusion of secondary cancers and 15 studies (43%) limited their cancers to incidence cases (**Supplementary Table 3B**). Overall, the methodological quality assessments of the 35 studies included in the meta-analysis yielded an average score of 4.7 out of 9.0 for the NOS and 22.0 out of 45.0 for our own detailed quality score (**Supplementary Tables 3A, B**). The quality of the 25 studies that were excluded from the meta-analysis was low (mean: 3.8 out of 9.0 points for the NOS and 13.5 out of 45.0 points for the detailed quality score, **Supplementary Tables 3A, B**). Their characteristics and main results are briefly described in **Supplementary Table 1**.

**Table 1:** Characteristics of studies included in meta-analysis of the associations between vaccination and childhood cancer, 1963–1978.

First Author, Year (Ref No.)	Location	Study Years	No. of Cases	No. of Controls	Age Range	Cancer Sites	Vaccines [Early Age]	Results	Outcome	Exposure	Study Design	Comment	Study Quality^d^
Innis, 1968 (53)^a^	Australia (Sydney, Brisbane)	1958–1967	816	816	children	Cancer	D, T, P, Polio, Sma, BCG, Typ, Cho [<1 y]	↑ risk after Polio vaccination >1year; others: no association	Hospital	Record	Case-control	[Mat: age, sex]; hospital- based without cancer; update of Innis 1965	15.8; 3
Davignon, 1970 (54)^a^	Canada (Quebec)	1960–1963	96	191	<15	Leukemia	BCG	↓ leukemia mortality rates in vaccinated group	Registry	Registry	Retrospective cohort	Mortality rate; irrelevant errors in table 1 corrected by Davignon 1971	23.9; 4
MRC, 1972 (60)^a^	England	1950–1952	65	54,174	15–30	Cancer, leukemia, lymphoma	BCG	No association	Follow- Up	Trial	Retrospective cohort	Mortality rate; outcome incidence and cancer deaths; trial-based; original study of Sutherland 1982	21.4; 5
Heinonen, 1973 (62)^a^	USA	1959–1965	24	50,873	0–4	Cancer, neural tumors, leukemia	Polio, Inf [prenatal]	↑ risk after prenatal killed polio; others: no association	Record	Self-report	Cohort	[Adj: race]; prenatal vaccination	19.6; 7
Mathé, 1974 (63)^a^	France	1965	130	130	<20	Leukemia	BCG	No association	Hospital	Vaccination card	Case-control	[Mat: age]; population-based without cancer; socioeconomical status not considered	11.2; 3
Comstock, 1975 (64)^a^	Puerto Rico	1949–1951	135	77,877	1–18	Cancer, leukemia, lymphoma, HL, brain, bone, skin, kidney, ...^b^	BCG	No association	Registry	Trial	Retrospective cohort	Trial based, trial arm according to birth year; original study of Snider 1978	25.1; 5
Salonen, 1975 (65)	Finland	1959–1968	972	972	<15	Cancer, leukemia, brain, eye, kidney, bone, other	Polio, BCG	No association	Registry	Record	Case-control	Mat: age, area, birth season; population-based without cancer; original study of Salonen 1976	25.8; 6
Crispen, 1976 (66)	USA (Chicago)	1957–1969	319	619,907	<20	Cancer, leukemia	BCG [newborns]	↓ risk for cancer death in vaccinated group	Death certificate	Record	Retrospective cohort	Mortality rate; update of Rosenthal 1972	21.5; 5
Salonen, 1976 (67)^a^	Finland	1959–1968	972	972	<15	Cancer, leukemia, brain, eye, kidney, bone, other tumors	Any, BCG	No association	Registry	Record	Case-control	[Mat: age, area, birth season]; population-based without cancer; update of Salonen 1975	22.4; 5
Andersen, 1978 (70)^a^	Denmark (Copenhagen)	1943–1970	63	182	school children	HL	BCG	No association	Registry	Record	Case-control	[Mat: age, sex, socioeconomical status]; 1:3; population-based without cancer; Fisher´s exact test	19.3; 3
Snider, 1978 (73)^a^	Puerto Rico	1949–1973	227	77,745	1–18	Cancer, leukemia, lymphoma, HL, brain, bone, skin, kidney, ... ^c^	BCG	No association	Registry	Trial	Retrospective cohort	Trial based, trial arm according to birth year; update von Comstock, 1975	24.5; 4

Adj, Adjustment; BCG, Bacillus Calmette–Guérin (vole bacillus; tuberculosis); Cho, Cholera; D, Diphtheria; Inf, Influenza; HL, Hodgkin lymphoma; Mat, Matching; MRC, Medical Research Council; Ref No., Reference number; Sma, Smallpox; Typ, Typhoid; y, years.

^a^Calculation of crude ORs.

^b^Cancer, leukemia, lymphoma, HL, nervous system, bone, kidney, ovary, male genitalia, skin, bladder, salivary glands, mouth, esophagus, stomach, colon, liver, larynx, lungs, breast, cervix, uterus, other endocrine organs, connective tissue.

^c^Cancer, leukemia, multiple myeloma, lymphatic tissue, HL, brain, other nervous system, bone, kidney, bladder, other urinary organs, ovary, prostate, other female/male genital organs, eye, skin, other skin, breast, bronchus and lung, cervix, connective tissue, esophagus, large intestine, larynx, liver, mouth, nose, other digestive organs, other endocrine glands, pancreas, peritoneum, rectum, salivary gland, stomach, thyroid, tonsils, uterus.

^d^Study quality with detailed quality score (−6 to 45 points) and Newcastle-Ottawa Scale (0 to 9 points).

**Table 2:** Characteristics of studies included in meta-analysis of the associations between vaccination and childhood cancer, 1979–1997.

First Author, Year (Ref No.)	Location		Study Years	No. of Cases	No. of Controls	Age Range	Cancer Sites	Vaccines [Early Age]	Results	Outcome	Exposure	Study Design	Comment	Study Quality^e^
Farwell, 1979 (74)^a^	USA (Connecticut)		1956–1962	120	240	≤19	Central nervous system, glioma, medulloblastoma	Polio [prenatal]	↑ risk for medullablastoma; others: no association	Registry	Self-report	Case-control	[Mat: age, sex, area of residence]; original study of Farwell 1984	15.8; 3
Neumann, 1980 (76)^a^	Germany		1972–1976	74	74	≤14	Cancer, leukemia	D, T, Polio, BCG, Pox	No association	Death certificate	Self-report	Case-control	Cancer death; [Mat: age, sex]; population-based; article in German	13.8; 3
Kendrick, 1981 (77)^a^	USA (Georgia, Alabama)		1950–1977	852	33,915	>5–<20cancer;>5 sub- sites	Cancer, leukemia, multiple myeloma, lymphoma, HL, bone, brain, skin, kidney, ...^d^	BCG	No association	–	Trial	Retrospective cohort	Trial-based; update of Comstock 1971	21.2; 3
Sutherland, 1982 (79)^a^		England	1950–1979	28	54,211	15–30	Leukemia	BCG	No association	Registry	Trial	Retrospective cohort	Mortality rate; trial-based; update/external validation of trial follow-up using registry data of MRC 1972	23.8; 5
Van Steensel-Moll, 1985 (81)		Netherlands	1973–1982	625	615	<15	Leukemia	Any [prenatal]	No association	Registry	Self-report	Case-control	Mat: age, sex, area; Adj: age, sex; population-based	21.8; 5
Kneale, 1986 (82)^b^	England (Oxford)		1953–1977	12,281	12,281	0–15	Cancer, leukemia, lymphoma, cerebral tumor, neuroblastoma, osteosarcoma, Wilms tumor, other solid tumors	Any [0–1 y], Sma, DT, P, Mea, Rub, Polio, BCG	↓ death risk for leukemia, Wilms tumor, neuroblastoma, cerebral tumor and other solid tumors; ↓ death risk for cancer onset age 0–1 after vaccination age 0–1, onset age 2–4 after vaccination age 0–1 and 2–4, onset age 10–15 after vaccination age 10–15 and all ages; others no association	Hospital	Self-report	Case-control	Cancer death; Mat: sex, area, birth date (birth year, season);% risk; population-based child alive; Update of Stewart 1965	19.6; 4
McKinney, 1987 (83)	England (West Midlands, North West, Yorkshire)		1980–1983	234	468	1–15	Leukemia, ML, lymphoma	Any (T, D, P, Polio, Mea, triple, Sma)	↓ risk for leukemia in general; no association for myeloid leukemia, leukemia/lymphoma, lymphoma	Registry	Self-report	Case-control	Mat: age, sex; hospital-based without cancer; original study of Hartley 1988	22.6; 4
Nishi, 1989 (86)	Japan (Hokkaido)		1986–1987	63	126	0–14	Non-T cell ALL	BCG, Mea [<2 y]	↓ risk	Hospital	Self-report	Case-control	Mat: age, sex, area; hospital- based	15.6; 2
Petridou, 1997 (91)^c^	Greece		1993–1994	153	300	0–14	Leukemia	DTP, BCG, viral (R, Mum, Mea, Hep)	No association	Hospital	Self-report	Case-control	Mat: age, sex, area; hospital- based without cancer	19.0; 4

Adj, Adjustment; ALL, Acute lymphoblastic leukemia; BCG, Bacillus Calmette–Guérin (vole bacillus; tuberculosis); D, Diphtheria; DT, Diphtheria-Tetanus; DTP, Diphtheria-Tetanus-Pertussis/Whooping cough; Hep, Hepatitis; HL, Hodgkin lymphoma; Mat, Matching; Mea, Measles; MRC, Medical Research Council; Mum, Mumps; Ref No., Reference number; Sma, Smallpox; y, years.

^a^Calculation of crude ORs.

^b^Calculation of crude ORs taking individual matching into account.

^c^Partly calculation of crude ORs not included in meta-analysis.

^d^Cancer, leukemia, multiple myeloma, lymphatic tissue, HL, brain, other nervous system, bone, kidney, bladder, other urinary organs, ovary, prostate, other female/male genital organs, eye, skin, other skin, breast, bronchus and lung, cervix, connective tissue, esophagus, large intestine, larynx, liver, mouth, nose, other digestive organs, other endocrine glands, pancreas, peritoneum, rectum, salivary gland, stomach, thyroid, tonsils, uterus.

^e^Study quality with detailed quality score (−6 to 45 points) and Newcastle-Ottawa Scale (0 to 9 points).

**Table 3:** Characteristics of studies included in meta-analysis of the associations between vaccination and childhood cancer, 1998–2004.

First Author, Year (Ref No.)	Location	Study Years	No. of Cases	No. of Controls	Age Range	Cancer Sites	Vaccines [Early Age]	Results	Outcome	Exposure	Study Design	Comment	Study Quality^a^
Kaatsch, 1998 (92)	Germany (West Germany)	1992–1994	2358	2588	0–14	Leukemia	Number	↓ risk for leukemia for 0–3and 4–6 *versus >*6 shots; other cancer (NHL, CNS, neuro- and nephroblastoma, bone, soft-tissue sarcoma) not indicated	Registry	Self-report	Case-control	Adj: socioeconomic status, urban-rural status; Mat: age, sex, area; population-based; update Kaatsch 1996 and original study Schüz 1999 and von Kries 2000	21.6; 3
Dockerty, 1999 (93)	New Zealand	1990–1993	121	121	0–14	Leukemia	Any, number, routine, DTP, DT, BCG, Hep and other [>3 m]; MMR and Mea [>9 m]; Polio [>6 m]; R [>15 m]	↓ risk for leukemia after 1–4 different vaccinations (adj. only for age and sex); others no association	Registry	Record (parent held)	Case-control	Adj: age, sex; Mat: age, sex; latency considered; population-based	24.2; 5
Groves, 1999 (94)	USA (IL, IN, IA, MI,MN, NJ, OH, PA, WI)	1989–1993	439	439	0–14	ALL	DTP, D, T, Polio, MMR, Hib	↓ risk for ALL after Hib (conjug.); others no association	–	Record	Case-control	Adj: age, sex, race, birth year, day care attendance, parental education, family income; Mat: age, race, telephone number; population-based	18.0; 4
Schüz, 1999 (95)	Germany	1980–1994	1,010	1,010	0–14	AL, ALL	Number (D, T, P, Polio, Mum, Mea, R, Sma, Men, routine)	↑ risk for leukemia for0–3 and 4–6 *versus >*6 vaccinations	Registry	Self-report	Case-control	Adj: socioeconomic status; Mat: sex, birth year; population-based non- diseased; update Kaatsch 1996 and 1998	22.2; 4
Auvinen, 2000 (96)	Finland	1985–1987	77	113,923	0–14	Leukemia, ALL	Hib (PRP-D) [3, 4,6, and 14/18 m]	No association	Registry	Trial	Retrospective cohort	Adj: other vaccinations; Trial- based	35.4; 6
Von Kries, 2000 (97)	Germany (Lower Saxony)	1988–1993	420	613	0–15	Cancer, leukemia, tumors	BCG [newborns]	No association	Registry	Self-report	Case-control	Adj: age, sex; Mat: age, sex; population-based without cancer; power only 50%; update Kaatsch 1996 and 1998and Schüz 1999	22.2; 5
Krone, 2003 (98)	UK, Bulgaria, Italy, Germany, Estonia, Israel, Austria, France	1994–1997	603	627	0+	Malignant melanoma	BCG, Sma, Inf	↓ risk for melanoma after BCG, smallpox, or both in total and in several single countries	Hospital	Self-report (some cards)	Case-control	Adj: age, sex, race, study center, skin type, pigmented naevi, sunburns, freckling index; population-based	24.4; 6
Frentzel- Beyme, 2004 (99)	Austria	1978–1988	88	208	8–25	Osteo- and Ewing- sarcoma, other bone tumors	D, T, P, Polio, BCG, Chi, vaccination reaction	↓ risk after repeated pertussis vaccination in girls univariate; others no association	Registry	Self-report	Case-control	Mat: age, sex; population- based, hospital-based	21.0; 5

Adj, Adjustment; AL, Acute leukemia; ALL, Acute lymphoblastic leukemia; BCG, Bacillus Calmette–Guérin (vole bacillus; tuberculosis); Chi, Chicken pox (varicella zoster); D, Diphtheria; DT, Diphtheria-Tetanus; DTP, Diphtheria-Tetanus-Pertussis/Whooping cough; Hep, Hepatitis; Hib, Haemophilus influenzae type b; Inf, Influenza; m, months; Mat, Matching; Mea, Measles; Men, Meningococcus; MMR, Mumps-Measles-Rubella; Mum, Mumps; NHL, Non-Hodgkin lymphoma; Ref No., Reference number; Sma, Smallpox; y, years.

^a^Study quality with detailed quality score (−6 to 45 points) and Newcastle-Ottawa Scale (0 to 9 points).

**Table 4:** Characteristics of studies included in meta-analysis of the associations between vaccination and childhood cancer, 2005–2019.

First Author, Year (Ref No.)	Location	Study Years		No. of Cases	No. of Controls	Age Range	Cancer Sites	Vaccines [Early Age]	Results	Outcome	Exposure	Study Design	Comment	Study Quality^b^
Ma, 2005 (100)	USA (California)		1995–2002	323	409	0–14	Leukemia, ALL	DPT, Polio, MMR,Hep [<1 y], Hib	↓ risk for leukemia and ALL after Hib vaccination; others no association	Registry	Vaccination card	Case-control	Adj: birth weight, day care attendance, family income, maternal education; Mat: age, sex, mother´s race, Hispanic status; population-based	23.2; 5
Mallol- Mesnard, 2007 (101)^a^	France	2003–2004		776	1681	<15	AL, ALL, AML	Number [6m]; BCG [newborns]; D, T, P, Hep, Hib, Pne, Men and Polio [<6 m]; Mum, Mea & R [1y]	↑ risk of AML after 1–2 vaccinations <6 months compared to ≥4 vaccinations; others no association	Registry	Vaccination card	Case-control	Adj: age, sex, birth order, maternal and paternal educational level, degree of urbanization; Mat: age, sex; population-based	27.8; 6
MacArthur, 2008 (103)	Canada	1990–1994		399	399	0–14	Leukemia, ALL	D, T, P, Polio, Mum, Mea, R, BCG, Hep, other	No association	Registry	Vaccination card	Case-control	Adj: race, family income, maternal education & age at birth, number of residences since birth; Mat: age, sex area; population-based	26.6; 5
Villumsen, 2009 (104)	Denmark	1965–1976		71	2,073	5–35	Lymphoma, NHL, HL, leukemia	BCG, Sma	↓ lymphoma risk after BCG; others: no association	Registry	Vaccination card	Retrospective case-cohort	Adj: day care, family social class; register-based; Sub- cohort; update of Danish data in Waaler 1970	27.6; 8
Pagaoa, 2011 (105)	USA (Texas)	1995–2006		2800	11,200	2–17	Cancer, ALL, NHL,medullablastoma	DTP, Polio, MMR,Chi, Hep, Hib, combination	↓ risk for all cancers and ALL after Hib and for ALL after combined vaccination by region; ↓ risk for all cancers and ALL after Hep and for ALL after IPV, Hep and combined vaccination, ↑ risk formedullablastoma after Hiband NHL after MMR by country	Registry	Registry	Ecological	Adj: age, sex, race, birth weight, birth year, birth type, birth order, premature birth, maternal education, maternal marital status, prior births, diabetes, preterm labor, tobacco use, and alcohol use, mother age at birth; Mat: sex, birth year; 1:4; population- based without cancer	13.7; 5
Soegaard, 2017 (108)	Denmark	1981–2008		490	1,224,914	0–14	ALL	DTPolio [5, 6,16 m], P (<3,10 m], MMR, Hib[3–16 m], routine vaccination	No association	Registry	Registry	Retrospective cohort	Adj: sex, race, birth weight, year, order and mode, other vaccination, gestational age; down syndrome excluded; latency considered; register- based; hazard ratio	37.0; 8
Figueroa, 2019 (109)	Costa Rica	1995–2003		240	578	1–15	ALL	Routine vaccination	↓ risk for ALL	Registry	Self-report	Case-control	Adj: sex, birth year, socioeconomic status	21.6; 6

Adj, Adjustment; AL, Acute leukemia; ALL, Acute lymphoblastic leukemia; AML, Acute myeloid leukemia; BCG, Bacillus Calmette–Guérin (vole bacillus; tuberculosis); Chi, Chicken pox (varicella zoster); D, Diphtheria; DTP, Diphtheria-Tetanus-Pertussis/Whooping cough; DTPolio, Diphtheria-Tetanus-Poliomyelitis; Hep, Hepatitis; Hib, Haemophilus influenzae type b; HL, Hodgkin lymphoma; m, months; Mat, Matching; Mea, Measles; Men, Meningococcus; MMR, Mumps-Measles-Rubella; Mum, Mumps; NHL, Non-Hodgkin lymphoma; Pne, Pneumococcus; Ref No., Reference number; Sma, Smallpox.

^a^ Partly calculation of crude ORs not included in meta-analysis.

^b^ Study quality with detailed quality score (−6 to 45 points) and Newcastle-Ottawa Scale (0 to 9 points).

### Results of the Meta-Analysis

**Figure 2:**
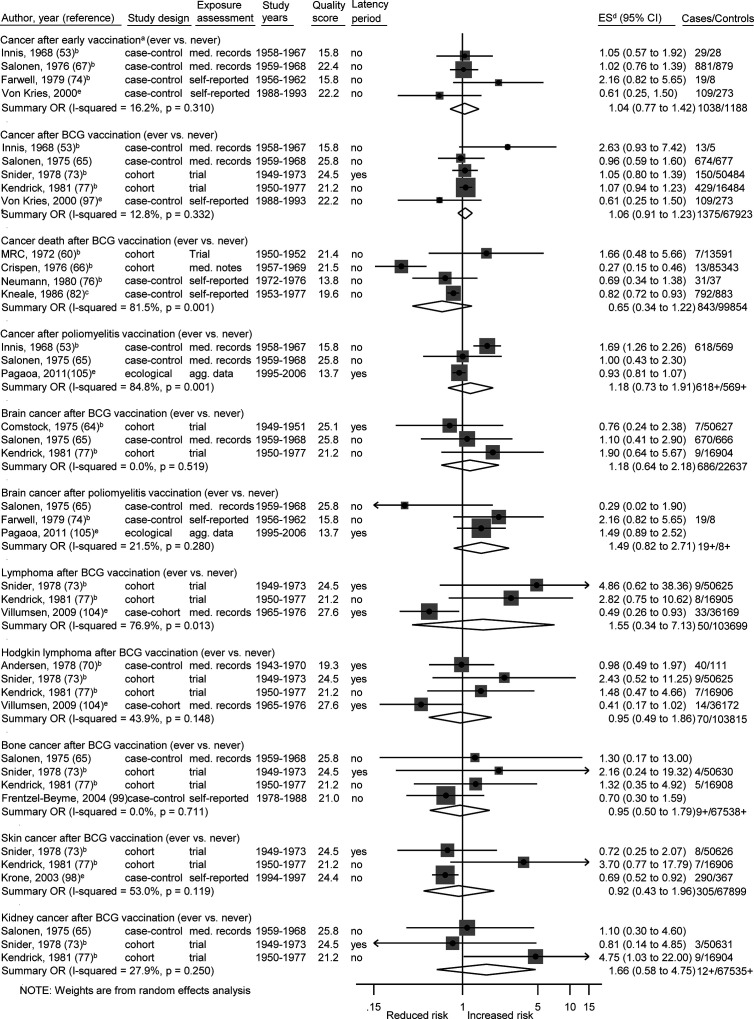
Vaccination and the risk of cancer. agg. data, aggregated data; BCG, Bacillus Calmette-Guérin; med. records, medical records; OR, odds ratio; ES, estimate. (a) Early vaccinations: Innis (poliomyelitis vaccination, age <1), Salonen (any vaccination, perinatal), Farwell (poliomyelitis vaccination, prenatal), von Kries (BCG vaccination, newborns). (b) Calculation of crude ORs. (c) Calculation of crude ORs taking individual matching into account. (d) ES includes single-study odds ratios or hazard ratios and summary odds ratios. (e) Adjusted estimate as indicated by published study.

Among 27 specific analyses on 11 different childhood cancer outcomes after exposure to nine different types of vaccinations (**Figures 2** **–** **4**), we observed four inverse associations between childhood leukemia and certain vaccines as well as after three or more vaccinations of any type.

**Figure 3:**
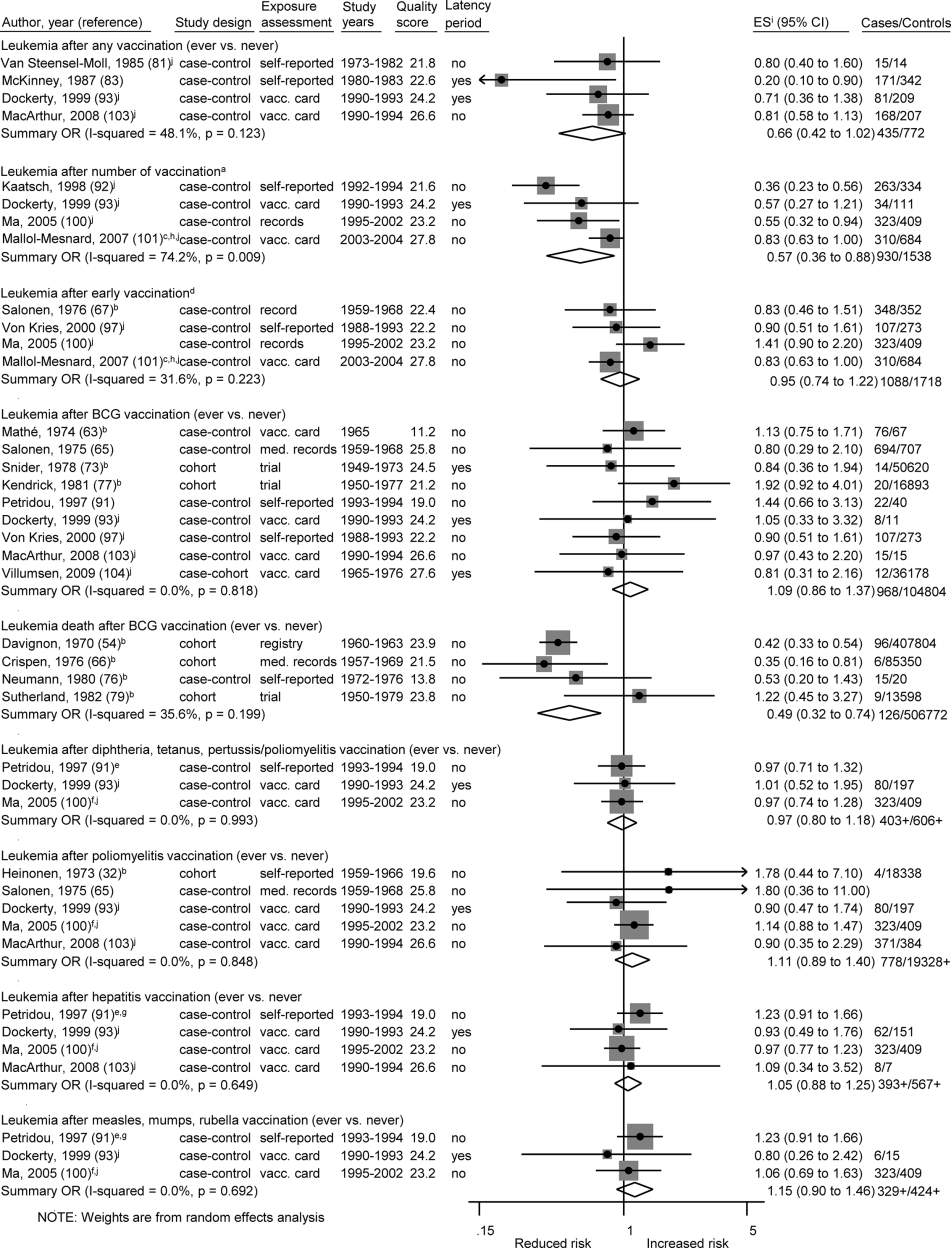
Vaccination and the risk of leukemia. BCG, Bacillus Calmette-Guérin; med. records, medical records; OR, odds ratio; ES, estimate. (a) Number of vaccinations: Kaatsch (any >6 *vs.* 0–3), Dockerty (any 3–4 *vs.* 0), Ma (Haemophilus influenzae type b 3+ *vs.* 1–2), Mallol-Messnard (any >3 *vs.* 3). (b) Calculation of crude ORs. (c) Estimates for acute leukemia. (d) Early vaccinations: Salonen (any ever *vs.* never, perinatal), Ma (hepatitis 3+ *vs.* 1–2, age <1), Mallol-Mesnard (any >3 *vs.* 3, age <0.5) von Kries (BCG vaccination, newborns). (e) Increment by ~3 doses. (f) Each additional dose. (g) Estimate for combination of measles, mumps, rubella, and hepatitis vaccination. (h) Inverted reference category. (i) ES includes single-study odds ratios or hazard ratios and summary odds ratios. (j) Adjusted estimate as indicated by published study.

**Figure 4:**
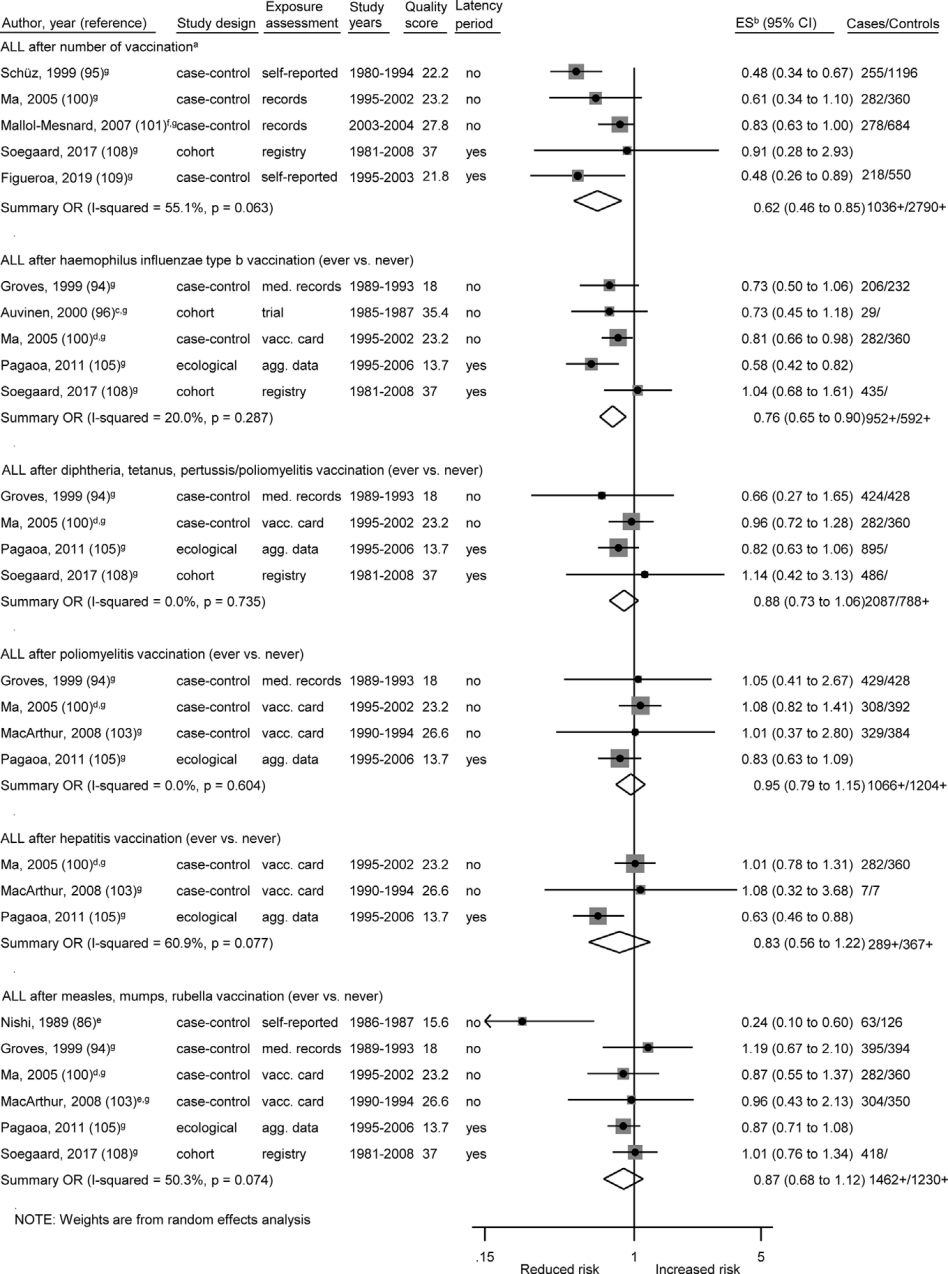
Vaccination and the risk of acute lymphoblastic leukemia (ALL). agg. data, aggregated data; ALL, acute lymphoblastic leukemia; med. records, medical records; OR, odds ratio; ES, estimate. (a) Number of vaccinations: Schüz (any >6 *vs.* 0–3), Ma (Haemophilus influenzae type b 3+ *vs.* 1–2), Mallol-Mesnard (any >3 *vs.* 3), Soegaard (complete *vs.* no/incomplete routine vaccination), Figueroa (complete *vs.* no/incomplete routine vaccination). (b) Calculation of crude ORs. (c) Early *vs.* late vaccination. (d) Each additional dose. (e) Estimate for measles. (f) Inverted reference category. (g) ES includes single-study odds ratios or hazard ratios and summary odds ratios. (h) Adjusted estimate as indicated by published study.

The summary OR of leukemia death was 0.49 (95% CI 0.32 to 0.74; I^2^ = 36%; N = 4; *P* value = 0.20) for bacillus Calmette–Guérin (BCG) vaccination compared to children without this vaccination (**Figure 3**). The four included studies were conducted between 1970 and 1982 and none of the studies accounted for a latency period. Three of the four studies (two cohort and one case-control study) reported a risk estimate below 1.0. Two obvious outlier studies were detected by omitting each study in turn from the meta-analysis (**Table 5B**). The observed risk reduction of the summary OR disappeared after the exclusion of Davignon et al. (54) or of Crispen et al. (66), which are two large and old cohort studies with valid exposure assessment *via* registry and medical documentation. Only the study of Neumann et al. (76) matched by age and sex, whereas the other three studies did not control for any confounders (54, 66, 79). Stratification by study period, study design, exposure assessment, and outcome assessment did not reveal any heterogeneity (I^2^ = 0%; **Supplementary Figures 1A–D**).

**Table 5:** Exclusion of single studies A) for **Figure 2**, B) for **Figure 3**, and C) for **Figure 4**.

Model description		OR (95% CI)	*I*-squared		*P* value	Model description		OR (95% CI)	*I*-squared	*P* value
**A) OMITTED STUDY ** **Figure 2**										
**Cancer after early vaccination^a^ (ever *vs.* never)**						**Leukemia after BCG vaccination (ever *vs.* never)**				
	Innis, 1968 (53)^b^	1.06 (0.63 to 1.79)	44.1%		0.167		Mathé, 1974 (63)^b^	1.07 (0.80 to 1.42)	0.0%	0.738
	Salonen, 1976 (67)^b^	1.09 (0.58 to 2.02)	43.7%		0.169		Salonen, 1975 (65)	1.11 (0.87 to 1.41)	0.0%	0.777
	Farwell, 1979 (74)^b^	0.98 (0.76 to 1.27)	0.0%		0.551		Snider, 1978 (73)^b^	1.11 (0.87 to 1.42)	0.0%	0.778
	Von Kries, 2000 (97)	1.09 (0.82 to 1.45)	6.0%		0.345		Kendrick, 1981 (77)^b^	1.02 (0.80 to 1.30)	0.0%	0.968
**Cancer after BCG vaccination (ever *vs.* never)**							Petridou, 1997 (91)	1.06 (0.83 to 1.35)	0.0%	0.796
	Innis, 1968 (53)^b^	1.05 (0.93 to 1.18)		0.0%	0.657		Dockerty, 1999 (93)	1.09 (0.86 to 1.38)	0.0%	0.732
	Salonen, 1975 (65)	1.07 (0.87 to 1.32)		32.1%	0.220		Von Kries, 2000 (97)	1.13 (0.87 to 1.46)	0.0%	0.789
	Snider, 1978 (73)^b^	1.06 (0.78 to 1.43)		34.5%	0.205		MacArthur, 2008 (103)	1.10 (0.86 to 1.40)	0.0%	0.741
	Kendrick, 1981 (77)^b^	1.05 (0.74 to 1.47)		34.0%	0.209		Villumsen, 2009 (104)	1.11 (0.87 to 1.41)	0.0%	0.776
	Von Kries, 2000 (97)	1.07 (0.95 to 1.22)		2.9%	0.378	**Leukemia death after BCG vaccination (ever *vs.* never)**				
**Cancer death after BCG vaccination (ever *vs.* never)**							Davignon, 1970 (54)^b^	0.59 (0.28 to 1.22)	45.2%	0.161
	MRC, 1972 (60)^b^	0.55 (0.27 to 1.11)		86.4%	0.001		Crispen, 1976 (66)^b^	0.57 (0.31 to 1.04)	53.7%	0.115
	Crispen, 1976 (66)^b^	0.82 (0.72 to 0.93)		0.0%	0.472		Neumann, 1980 (76)^b^	0.50 (0.28 to 0.89)	55.8%	0.104
	Neumann, 1980 (76)^b^	0.65 (0.27 to 1.58)		87.6%	0.000		Sutherland, 1982 (79)^b^	0.42 (0.33 to 0.53)	0.0%	0.820
	Kneale, 1986 (82)^c^	0.60 (0.23 to 1.56)		77.8%	0.011	**Leukemia after diphtheria, tetanus, pertussis/poliomyelitis vaccination (ever *vs.* never)**				
**Cancer after poliomyelitis vaccination (ever *vs.* never)**							Petridou, 1997 (91)^h^	0.98 (0.76 to 1.26)	0.0%	0.912
	Innis, 1968 (53)^b^	0.93 (0.81 to 1.07)		0.0%	0.867		Dockerty, 1999 (93)	0.97 (0.79 to 1.19)	0.0%	0.909
	Salonen, 1975 (65)	1.24 (0.69 to 2.22)		92.4%	0.000		Ma, 2005 (100)^i^	0.98 (0.74 to 1.29)	0.0%	0.914
	Pagaoa, 2011 (105)	1.52 (1.00 to 2.30)		25.5%	0.247	**Leukemia after poliomyelitis vaccination (ever *vs.* never)**				
**Brain cancer after BCG vaccination (ever *vs.* never)**							Heinonen, 1973 (62)^b^	1.10 (0.88 to 1.38)	0.0%	0.818
	Comstock, 1975 (64)^b^	1.40 (0.68 to 2.91)		0.0%	0.465		Salonen, 1975 (65)	1.11 (0.88 to 1.39)	0.0%	0.784
	Salonen, 1975 (65)	1.22 (0.50 to 3.00)		22.1%	0.257		Dockerty, 1999 (93)	1.15 (0.90 to 1.46)	0.0%	0.823
	Kendrick, 1981 (77)^b^	0.94 (0.45 to 1.98)		0.0%	0.631		Ma, 2005 (100)^i^	1.03 (0.64 to 1.67)	0.0%	0.742
**Brain cancer after poliomyelitis vaccination (ever *vs.* never)**							MacArthur, 2008 (103)	1.13 (0.89 to 1.43)	0.0%	0.762
	Salonen, 1975 (65)	1.62 (1.02 to 2.56)		0.0%	0.507	**Leukemia after hepatitis vaccination (ever *vs.* never)**				
	Farwell, 1979 (74)^b^	0.97 (0.24 to 3.98)		47.0%	0.170		Petridou, 1997 (91)^h,j^	0.97 (0.78 to 1.20)	0.0%	0.973
	Pagaoa, 2011 (105)	1.04 (0.16 to 6.92)		60.5%	0.112		Dockerty, 1999 (93)	1.06 (0.88 to 1.27)	0.0%	0.474
**Lymphoma after BCG vaccination (ever *vs.* never)**							Ma, 2005 (100)^i^	1.17 (0.89 to 1.52)	0.0%	0.735
	Snider, 1978 (73)^b^	1.06 (0.19 to 5.84)		81.6%	0.020		MacArthur, 2008 (103)	1.05 (0.88 to 1.25)	0.0%	0.440
	Kendrick, 1981 (77)^b^	1.24 (0.14 to 11.27)		76.9%	0.038	**Leukemia after mumps, measles, rubella vaccination (ever *vs.* never)**				
	Villumsen, 2009 (104)	3.31 (1.08 to 10.10)		0.0%	0.664		Petridou, 1997 (91)^h,j^	1.02 (0.68 to 1.53)	0.0%	0.645
**Hodgkin lymphoma after BCG vaccination (ever *vs.* never)**							Dockerty, 1999 (93)	1.17 (0.92 to 1.50)	0.0%	0.578
	Andersen, 1978 (70)^b^	1.01 (0.34 to 3.01)		61.9%	0.073		Ma, 2005 (100)^i^	1.19 (0.89 to 1.60)	0.0%	0.465
	Snider, 1978 (73)^b^	0.81 (0.41 to 1.62)		43.9%	0.168	**C) OMITTED STUDY ** **Figure 4**				
	Kendrick, 1981 (77)^b^	0.86 (0.37 to 2.00)		55.4%	0.106	**ALL after number of vaccination^k^**				
	Villumsen, 2009 (104)	1.22 (0.70 to 2.12)		0.0%	0.531		Schüz, 1999 (95)^e^	0.74 (0.58 to 0.94)	11.3%	0.336
**Bone cancer after BCG vaccination (ever *vs.* never)**							Ma, 2005 (100)	0.67 (0.42 to 1.07)	73.8%	0.022
	Salonen, 1975 (65)	0.92 (0.47 to 1.79)		0.0%	0.526		Mallol-Mesnard, 2007 (101)^e^	0.52 (0.40 to 0.66)	0.0%	0.685
	Snider, 1978 (73)^b^	0.88 (0.45 to 1.71)		0.0%	0.677		Soegaard, 2017 (108)	0.61 (0.43 to 0.85)	65.8%	0.033
	Kendrick, 1981 (77)^b^	0.85 (0.41 to 1.77)		0.0%	0.591		Figueroa, 2019 (109)	0.65 (0.46 to 0.94)	61.6%	0.050
	Frentzel-Beyme, 2004 (99)	1.46 (0.54 to 3.96)		0.0%	0.925	**ALL after Haemophilus influenzae type b vaccination (ever *vs.* never)**				
**Skin cancer after BCG vaccination (ever *vs.* never)**							Groves, 1999 (94)	0.77 (0.62 to 0.95)	39.1%	0.178
	Snider, 1978 (73)^b^	1.33 (0.27 to 6.62)		76.5%	0.039		Auvinen, 2000 (96)^l^	0.76 (0.62 to 0.94)	39.5%	0.175
	Kendrick, 1981 (77)^b^	0.69 (0.53 to 0.91)		0.0%	0.939		Ma, 2005 (100)^i^	0.74 (0.58 to 0.94)	31.9%	0.221
	Krone, 2003 (98)	1.47 (0.30 to 7.19)		65.2%	0.090		Pagaoa, 2011 (105)	0.81 (0.70 to 0.95)	0.0%	0.623
**Kidney cancer after BCG vaccination (ever *vs.* never)**							Soegaard, 2017 (108)	0.74 (0.64 to 0.86)	0.0%	0.416
	Salonen, 1975 (65)	2.09 (0.37 to 11.77)		53.8%	0.141	**ALL after diphtheria, tetanus, pertussis/poliomyelitis vaccination (ever *vs.* never)**				
	Snider, 1978 (73)^b^	2.19 (0.52 to 9.16)		48.8%	0.162		Groves, 1999 (94)	0.89 (0.74 to 1.07)	0.0%	0.644
	Kendrick, 1981 (77)^b^	0.98 (0.33 to 2.91)		0.0%	0.790		Ma, 2005 (100)	0.82 (0.65 to 1.05)	0.0%	0.728
**B) OMITTED STUDY ** **Figure 3**							Pagaoa, 2011 (105)	0.94 (0.72 to 1.23)	0.0%	0.688
**Leukemia after any vaccination (ever *vs.* never)**							Soegaard, 2017 (108)	0.87 (0.72 to 1.05)	0.0%	0.605
	Steensel-Moll, 1985 (81)	0.58 (0.31 to 1.10)		64.9%	0.058	**ALL after poliomyelitis vaccination (ever *vs.* never)**				
	McKinney, 1987 (83)	0.79 (0.60 to 1.04)		0.0%	0.942		Groves, 1999 (94)	0.95 (0.79 to 1.15)	0.0%	0.405
	Dockerty, 1999 (93)	0.60 (0.31 to 1.15)		65.4%	0.056		Ma, 2005 (100)^i^	0.86 (0.66 to 1.10)	0.0%	0.846
	MacArthur, 2008 (103)	0.55 (0.27 to 1.11)		57.8%	0.093		MacArthur, 2008 (103)	0.95 (0.79 to 1.15)	0.0%	0.399
**Leukemia after number of vaccination^d^**							Pagaoa, 2011 (105)	1.07 (0.83 to 1.38)	0.0%	0.991
	Kaatsch, 1998 (92)^e^	0.73 (0.55 to 0.95)		20.3%	0.285	**ALL after hepatitis vaccination (ever *vs.* never)**				
	Dockerty, 1999 (93)	0.56 (0.32 to 0.97)		82.7%	0.003		Ma, 2005 (100)^i^	0.65 (0.48 to 0.89)	0.0%	0.403
	Ma, 2005 (100)	0.56 (0.31 to 1.03)		81.9%	0.004		MacArthur, 2008 (103)	0.81 (0.51 to 1.28)	79.9%	0.026
	Mallol-Mesnard, 2007 (101)^e,f^	0.45 (0.33 to 0.61)		0.0%	0.382		Pagaoa, 2011 (105)	1.01 (0.79 to 1.31)	0.0%	0.916
**Leukemia after early vaccination^g^**						**ALL after mumps, measles, rubella vaccination (ever *vs.* never)**				
	Salonen, 1976 (67)^b^	0.99 (0.71 to 1.39)		53.2%	0.118		Nishi, 1989 (86)	0.93 (0.80 to 1.08)	0.0%	0.824
	Von Kries, 2000 (97)	0.97 (0.69 to 1.38)		54.4%	0.112		Groves, 1999 (94)	0.83 (0.63 to 1.10)	55.8%	0.060
	Ma, 2005 (100)	0.84 (0.69 to 1.03)		0.0%	0.967		Ma, 2005 (100)^i^	0.86 (0.63 to 1.18)	60.2%	0.040
	Mallol-Mesnard, 2007 (101)^e,f^	1.07 (0.76 to 1.51)		20.2%	0.285		MacArthur, 2008 (103)	0.86 (0.65 to 1.14)	60.1%	0.040
							Pagaoa, 2011 (105)	0.84 (0.58 to 1.23)	59.6%	0.042
							Soegaard, 2017 (108)	0.81 (0.58 to 1.15)	56.2%	0.058

ALL, Acute lymphoblastic leukemia; BCG, Bacillus Calmette–Guérin (vole bacillus, tuberculosis); CI, confidence interval; OR, odds ratio.

^a^ Early vaccinations: Innis (poliomyelitis vaccination ever vs. never, age <1), Salonen (any vaccination, newborns), Farwell (poliomyelitis vaccination ever vs. never, prenatal).

^b^ Calculation of crude ORs.

^c^ Calculation of crude ORs taking individual matching into account.

^d^ Number of vaccinations: Kaatsch (any >6 vs. 0–3), Dockerty (any 5+ vs. 0), Ma (Haemophilus influenzae type b 3+ vs. 1–2), Mallol-Messnard (any >3 vs. 3).

^e^ Inverted reference category.

^f^ Estimates for Acute Leukemia.

^g^ Early vaccinations: Salonen (any ever vs. never, perinatal), Ma (hepatitis 3+ vs. 1–2, age <1), Mallol-Mesnard (any >3 vs. 3, age <0.5).

^h^ Increment by ~3 doses.

^i^ Each additional dose.

^j^ Estimate for combination of hepatitis and MMR vaccine.

^k^ Number of vaccinations: Schüz (any >6 vs. 0–3), Ma (Haemophilus influenzae type b 3+ vs. 1–2), Mallol-Mesnard (any >3 vs. 3), Soegaard (complete vs. no/incomplete routine

^l^ Early vs. late vaccination.

The association between ALL and Haemophilus influenzae type b (Hib) vaccination was assessed based on five studies, the OR was 0.76 (0.65 to 0.90; I² = 20%; N = 5, *P* = 0.29; **Figure 4**). Four of the five studies (one cohort, two case-control, one ecological study) reported a risk estimate below 1.0 (**Figure 4**). Omitting each study in turn from the meta-analysis did not reveal an obvious outlier among the five studies and all summary ORs still showed a risk reduction after any one study was excluded (**Table 5C**). The included studies were conducted between 1999 and 2017. All studies had a good assessment of the outcome and controlled for other vaccinations or other exposures in the immunological pathway e.g. infections. The stratification by study design, exposure assessment, inclusion of a latency period, and study quality did not show any heterogeneity (I^2^ = 0%; **Supplementary Figures 1B, C, E, F**).

Of two analyses focusing on the number of vaccine injections, one showed a risk reduction for leukemia (OR = 0.57; 0.36 to 0.88; N = 4; I^2^ = 74%; *P* value = 0.01) and one for ALL (OR = 0.62; 0.46 to 0.85; N = 5; I^2^ = 55%; *P* value = 0.06) after three or more vaccinations of any type. For both associations, all studies reported a risk estimate smaller than 1.0. Omitting each study in turn from the two analyses revealed obvious outlier studies for both associations. The heterogeneity across studies disappeared after exclusion of the German studies (92, 95) or the French study (101) and the summary OR was no longer significant after exclusion of the US study (100) (**Tables 5B, C**). The included studies covered together a study period of 29 years (1980 to 2008) for ALL and of 15 years (1990 to 2004) for leukemia. Most investigations were case-control studies (92, 93, 95, 100, 101, 109) and only one cohort study (108) was included in the meta-analysis for ALL. This Danish study was also the only study with exposure assessment based on a medical registry. The stratification by study design, exposure assessment, and consideration of a latency period did not show any heterogeneity (I^2^ = 0%; **Supplementary Figures 1B, C, E**). After stratification by study quality and adjustment, substantial heterogeneity was observed with a stronger risk reduction of ALL and leukemia for basic adjustment (ALL: OR = 0.48; 0.36 to 0.64; N = 2; leukemia: OR = 0.41; 0.27 to 0.61; N = 2) as compared to advanced adjustment (ALL: OR = 0.80; 0.65 to 0.99; N = 3; leukemia: OR = 0.73; 0.50 to 1.06; N = 2; **Supplementary Figure 1G**) and a significant risk reduction of ALL and leukemia for low quality below 24.7 points (ALL: OR = 0.50; 0.39 to 0.65; N = 3; leukemia: OR = 0.45; 0.33 to 0.91; N = 3) compared to a non-significant risk reduction for high quality equal or above 24.7 points (ALL: OR = 0.88; 0.66 to 1.04; N = 2; leukemia: OR = 0.83, 0.66 to 1.05; N = 1; **Supplementary Figures 1F, H**). In addition, a dose-response analysis was conducted to assess the risk of leukemia for an increasing number of vaccine injections (**Supplementary Figure 2**). The observed risk reduction was also observed in this analysis, even though not significant (OR = 0.94; 0.89 to 1.00; N = 2; I^2^ = 0%; *P* value = 0.54). However, trends required for the dose-response analysis could only be calculated for the studies of Kaatsch (92) and Dockerty (93). The other two studies had to be excluded due to an insufficient number of reported estimates (100) and significant deviations between the reported estimates of the different vaccine injections (101). For the same reasons, it was not possible to conduct a dose-response analysis for number of vaccine injections and ALL.

The remaining 22 specific analyses did not show any association between different types of vaccination (any, early, BCG, poliomyelitis, hepatitis, diphtheria-tetanus-pertussis/-poliomyelitis, measles-mumps-rubella) and overall childhood cancer risk, cancer death, or site-specific cancers (lymphoma, Hodgkin lymphoma, bone cancer, brain cancer, kidney cancer, skin cancer, leukemia, ALL; **Figures 2**–**4**). We observed substantial heterogeneity for cancer death after BCG vaccination (OR = 0.65; 0.34 to 1.22; N = 4; I^2^ = 82%; *P* value < 0.01), for cancer after poliomyelitis vaccination (OR = 1.18; 0.73 to 1.91; N = 3; I^2^ = 85%; *P* value <0.01), and for lymphoma after BCG vaccination (OR = 1.55; 0.34 to 7.13; N = 3; I^2^ = 77%; *P* value = 0.01). In each of these analyses, heterogeneity disappeared (I^2^ = 0%) after exclusion of one specific outlier study. However, the outlier studies differ in different analyses (**Tables 5A–C**). The stratified results are shown in **Supplementary Figures 1A–H**. Overall, no evidence of publication bias was seen in analyses including five or more studies either when using the funnel plot or when using the test by Egger et al. (48) (**Supplementary Figures 3**-**8A–C**).

## Discussion

We observed an inverse association between BCG vaccination and leukemia death, between Hib vaccination and ALL, and between a high number of unspecified vaccinations and ALL or leukemia. The other 23 conducted analyses did show any associations. Despite the fact that we included a large number of publications over a long time period, the question of a possible risk reduction of childhood cancer after vaccination has not yet been finally clarified in our review and meta-analysis, since the exposure assessment of many studies has limited validity. This might be one of the reasons why published results are inconsistent. Some of these studies also have insufficient statistical power. In addition, most studies suffer from further methodological limitations, especially regarding the consideration of confounder control and latency periods. For most specific associations of interest, only few studies were available for pooling.

The risk reduction of ALL after Hib vaccination by 24% was fairly consistent across studies and by study characteristics. Only a recent Danish cohort study (108), that had the highest quality score (37.0), showed no association. However, we observed no heterogeneity after the stratification by study quality. A potential explanation for the link between Hib vaccination and ALL risk might be activation of the immune system early in life (21). ALL can frequently be traced back to a pre-leukemic clone carrying a prenatal genetic lesion (13, 110, 111). Postnatal acquired mutations then drive clonal evolution towards overt ALL. The protective role of vaccination in the development of ALL is based on the hypothesis that vaccines like Hib stimulate early formation of antibodies, prevent other infections, and modulate future responses to common childhood infections (23, 112). In line with this, mechanistic studies with mice that were repeatedly exposed to inflammatory stimuli, paralleling chronic infections in childhood, demonstrated that two enzymes, AID and RAG1-RAG2, drive clonal evolution of the most common subtype of ALL, B-cell precursor ALL (113). In addition, *in vivo* genetic studies connected inherited susceptibility to B-cell precursor ALL with postnatal infections by showing that B-cell precursor ALL was initiated in Pax5 heterozygous mice only when they were exposed to common pathogens (114). Moreover, among children in a large population-based birth cohort study, associations were observed between seven investigated serum immunoglobulin G titers and 10 exposures, either administered vaccines (e.g. BCG vaccination) or infections (115). These results indicate the existence of associations between immunogenic exposures and unrelated antibody titers, which may be responsible for non-specific effects of vaccinations on all-cause morbidity and mortality among children. Thus, early exposure to Hib vaccination may be responsible for the observed inverse association regarding ALL risk in our meta-analysis.

Our meta-analysis also showed a risk reduction for leukemia death after BCG vaccination in childhood, but not for the development of leukemia itself. The analyses on leukemia death and cancer death were limited to studies on relative risks or odds ratios of death among vaccinated and unvaccinated children. The study populations consisted of vaccination cohorts with vaccinated and unvaccinated children (60, 79), cohort studies calculating cancer mortality within the vaccinated and unvaccinated population (54, 66), or case-control studies with vaccinated and unvaccinated cancer deaths and healthy controls (76, 82). The observed risk reduction for leukemia death after BCG vaccination was mainly driven by two cohort studies (54, 66) with valid exposure and outcome assessment but without control for any confounders. We did not observe any heterogeneity between the four included studies in the stratified results.

In addition, studies comparing a high *versus* a low number of unspecified vaccinations observed an inverse association with ALL (95, 100, 101, 108, 109) and leukemia (92, 93, 100, 101). While this design of unspecified vaccinations mitigates some sources of confounding and bias, we noticed moderate heterogeneity across the study estimates of number of vaccinations. This heterogeneity disappeared after the exclusion of two large German case-control studies where both exposure assessment (interview of parents) and control of confounders (birth year, sex, socioeconomic status, and area) were suboptimal (92, 95). Assessment of the number of vaccinations was based on objective records in other studies but there were still differences (medical records, vaccination cards, medical claims data) that may affect the degree of misclassification (116). Claims data are assumed to have the highest validity regarding information on vaccination and were used in a large Danish cohort study (108). This high-quality study, which also carefully adjusted for confounders and considered latency periods, did not observed an association between number of vaccinations and ALL. In contrast, most other studies analyzing number of vaccinations (92, 95, 100, 101) and all studies that examined BCG vaccination (54, 66, 76, 79) did not take into account a latency period. However, this would result in non-differential misclassification and bias toward the null.

We did not find any other significant risk reduction of childhood cancer other than leukemia in our meta-analysis. With respect to poliomyelitis vaccination, the results of studies included in our meta-analysis were not consistent. Between 1955 and 1963, some poliomyelitis vaccines have been contaminated by simian virus 40 (34) that has the potential to initiate malignancy in various target tissues. This may explain the increased risk of childhood cancer for poliomyelitis vaccinations before 1963 with good exposure assessment. However in our stratified analyses, the increased risk of cancer after poliomyelitis immunization was only observed without consideration of a latency period, insufficient confounder control, low assessment of outcome, and low overall study quality. Moreover, there were also methodological limitations in the recent study that used an ecological design and did not show a correlation between childhood cancer and poliomyelitis vaccination (105). In such a study, an ecological bias may be introduced since only aggregated data are available. In addition, we observed substantial heterogeneity for the analysis of lymphoma after BCG vaccination. The recent Danish case-cohort study from Villumsen et al. (104) with very reliable vaccination information on an individual level, consideration of a latency period, and good confounder control indicated a beneficial effect of BCG vaccination on the risk of lymphomas. However, the other two old cohort studies in this analysis (73, 77), each with less than 10 cases and low overall quality, did not support this result.

In contrast to the conducted meta-analysis by Morra et al. (32), we did not observed an inverse association between leukemia and early vaccination before the age of 1 year in our synthesis. These different meta-analysis results can be explained by our additional inclusion of the study from Ma et al. (100) from 2005, which did not show any association between early vaccination and leukemia with careful adjustment. In addition contrary to our analysis, the meta-analysis of Morra et al. (32) included two old and large studies (54, 66) on leukemia death after early BCG vaccination, which found a strong risk reduction without control for any confounders. We preferred to analyze these studies on BCG vaccination and early immunization based on the different outcomes, leukemia death, and leukemia incidence, separately.

Our meta-analysis has specific strengths including the extensive search strategy we used to ensure that all relevant publications on this topic were identified. This enabled us to conduct separate analyses on histological and site-specific childhood cancers as well as on certain vaccines, age at vaccination, and number of vaccinations. To consider the overall study quality, we used the established NOS as well as our own more detailed quality assessment scale. The latter additionally considered important issues such as latency periods, quality of statistical methods, training of interviewers, exposure assessment in cohort studies, and cancer among controls of case-control studies. There was no conclusive evidence of publication bias, but the power of the test is poor in a meta-analysis with only a few included studies. However, a graphical examination of the plots also did not suggest a publication bias.

The small number of studies for each exposure-disease association and the relatively high level of heterogeneity across studies in some of our analyses is the main limitation of this meta-analysis. When the number of studies and the true fraction of heterogeneity is small, there appears a substantial positive bias for I^2^ but this bias is typically negative when the true fraction of heterogeneity is large and the number of studies is small (117). To account for potential heterogeneity we used random effects models and to assess its effect on our results, we stratified our analyses by study characteristics.

In conclusion, we found evidence of an inverse association between BCG vaccination and leukemia death, Hib vaccination and ALL, and a high number of unspecified vaccinations and ALL as well as leukemia. However, these results should be interpreted with caution given the small number of studies, no consideration of latency, and limited exposure assessment in some studies as well as insufficient confounder adjustment, in particular for infections. All studies included in this review and meta-analysis had at least one of these substantial limitations. Finally, although risk reductions after vaccination appear biologically plausible in leukemia, studies on dose effect and on age at vaccination with good exposure assessment and advanced confounder controlling are rare. Large cohort studies with valid assessment of immunizations, adequate consideration of the latency period, and detailed information on possible confounders (e.g. infections and other vaccines) are needed to assess the association between different types of vaccinations and specific childhood cancers.

## Data Availability Statement

The original contributions presented in the study are included in the article/**Supplementary Material**. Further inquiries can be directed to the corresponding author.

## Author Contributions

MM designed the meta-analysis, the analytic strategies and the detailed quality score, and directed its implementation, including quality assurance and control. She was in charge of creating the manuscript and conducting the literature review and the meta-analysis. LB and PK assisted in conducting the literature review and meta-analysis including acquisition of data, quality assessment, and preparation of figures and tables. LC assisted conducting the literature review including database searches, selections, and assessment of publications. IL, UH, and WA contributed to the analyses and interpretation of the results. WA assumes an overall scientific responsibility during the creation of the publication and thus assumes a particular responsibility for the quality and integrity of the publication. All authors revised the manuscript critically for important intellectual content and agreed to be accountable for all aspects of the work in ensuring that questions related to the accuracy or integrity of any part of the work are appropriately investigated and resolved. All authors contributed to the article and approved the submitted version.

## Conflict of Interest

The authors declare that the research was conducted in the absence of any commercial or financial relationships that could be construed as a potential conflict of interest.

## Abbreviations

Adj, adjustment; AL, acute leukemia; ALL, acute lymphoblastic leukemia; AML, acute myeloid leukemia; BCG, bacillus Calmette–Guérin (vole bacillus; tuberculosis); BCP, B-cell precursor; cALL, common acute lymphoblastic leukemia; Chi, chicken pox (varicella zoster); Cho, cholera; CI, confidence interval; CNS, central nervous system; D, diphtheria; DT, diphtheria-tetanus; DTP, diphtheria-tetanus-pertussis/whooping cough; DTPolio, diphtheria-tetanus-poliomyelitis; Exc, exclusion for meta-analysis; Hep, hepatitis; Hib, *Haemophilus influenzae* type b; Inf, influenza; IPV, inactivated poliomyelitis vaccine; HL, Hodgkin lymphoma; m, months; Mat, matching; Mea, measles; Men, meningococcus; MMR, measles-mumps-rubella; MRC, Medical Research Council; Mum, mumps; NHL, non-Hodgkin lymphoma; OR, odds ratio; P, points; Pne, pneumococcus; Polio, poliomyelitis; SE, standard error; Sma, smallpox; Typ, typhoid; y, years; Yel, yellow fever.
