# Supplementary material for: Vaccination and the Risk of Childhood Cancer—A Systematic Review and Meta-Analysis
Source: Front Oncol. 2021 Jan 22;10:610843. doi: 10.3389/fonc.2020.610843 (PMC7862764; doi:10.3389/fonc.2020.610843)
Supplement: Supplementary file 1 [file DataSheet_1.pdf]

Supplementary Table 1. Characteristics of included and excluded studies in the meta-analysis of the associations between vaccination and childhood cancer, 1963-2017

First Author, Year	Location	Study Years	No. of Cases	No. of Controls	Age Range	Cancer Sites	Vaccines [Early Age]	Results	Outcome	Exposure	Study Design	Comment	Study Quality[g]
Fraumeni, 1963	USA	1950-1959	-	-	>1	Cancer	Polio	No association	-	Record	Ecological	Exc: without complete estimates; cancer death	6.9 3
Innis, 1965	Australia (Brisbane)	1958-1964	59	343	children	Leukemia	Any (D, P, T) [>3m]	↑ risk	Hospital	Record	Case-control	Exc: without complete estimates; hospital-based; pilot study; letter to editor; original study of Innis 1968	12.2 2
Stewart, 1965[a]	England (Oxford)	1953-1965	2107	2107	<9	Cancer, leukemia	D, T, P, Sma, Polio, BCG, Yel, Typ	No association	Hospital	Self-report	Case-control	Exc: overlapping population; cancer death; population-based; letter to the editor; original study of Kneale 1986 & Gilman 1989	7.7 2
Innis, 1968[a,d]	Australia (Sydney, Brisbane)	1958-1967	816	816	children	Cancer	D, T, P, Polio, Sma, BCG, Typ, Cho [<1y]	↑ risk after polio vaccination >1year; others: no association	Hospital	Record	Case-control	[Mat: age, sex;] hospital-based without cancer; update of Innis 1965	15.8 3
Davignon, 1970[a,d]	Canada (Quebec)	1960-1963	96	191	<15	Leukemia	BCG	↓ leukemia mortality rates in vaccinated group	Registry	Registry	Retrospective cohort	Mortality rate; irrelevant errors in table 1 corrected by Davignon 1971	23.9 4
Waaler, 1970	Norway, Denmark, Sweden	1964-1968	-	-	0-43	Leukemia	BCG	No association	-	-	Ecological	Exc: without complete estimates; age specific mortality; letter to editor; original study of Villumsen 2009	-3.4 3
Berkeley, 1971	Scotland (Glasgow, Dundee, Aberdeen, Edinburgh)	1939-1968	-	-	0-14	Leukemia	BCG	↑ leukemia mortality after BCG in all age groups	Registry	-	Ecological	Exc: without complete estimates; outcome cancer deaths; letter to editor	4.2 3
Comstock, 1971[a]	USA (Georgia, Alabama)	1950-1971	60	-	>5	Leukemia, lymphoma, HL	BCG	No association	Hospital	Trial	Retrospective cohort	Exc: overlapping population; [Adj: age, race;] trial-based; original study of Kendrick 1981	20.8 4
Hems, 1971	England, Wales	1940-1970	-	-	10-29	Leukemia	BCG	↓ leukemia mortality	-	-	Ecological	Exc: without complete estimates; cancer deaths; letter to editor; latency considered	-2.2 3
Kinlen, 1971	Canada (Quebec), Scotland (Glasgow)	1950-1969	-	-	<19	Leukemia	BCG	No association	Registry	Registry	Ecological	Exc: without complete estimates; standardized incidence ratio	4.9 3
MRC, 1972[a,d]	England	1950-1952	65	54174	15-30	Cancer, leukemia, lymphoma	BCG	No association	Follow-Up	Trial	Retrospective cohort	Mortality rate; outcome incidence & cancer deaths; trial-based; original study of Sutherland 1982	21.4 5

Table continues

Supplementary Table 1. Continued

First Author, Year	Location	Study Years	No. of Cases	No. of Controls	Age Range	Cancer Sites	Vaccines [Early Age]	Results	Outcome	Exposure	Study Design	Comment	Study Quality[g]
Rosenthal, 1972[a]	USA (Chicago)	1964-1969	22	227378	0-6	Leukemia	BCG [newborns]	↑ mortality rate in unvaccinated infants	Registry	Record	Retrospective cohort	Exc: overlapping population; mortality rate; immigration not factored; [Adj: age race;] original study of Crispen 1976	23.57
Heinonen, 1973[a,d]	USA	1959-1965	24	50873	0-4	Cancer, Neural tumors, leukemia	Polio, Inf [prenatal]	↑ risk after prenatal killed polio; others: no association	Record	Self-report	Cohort	[Adj: race;] prenatal vaccination	19.67
Mathé, 1974[a,d]	France	1965	130	130	<20	Leukemia	BCG	No association	Hospital	Vaccination card	Case-control	[Mat: age]; population-based without cancer; socioeconomical status not considered	11.23
Comstock, 1975[a,d]	Puerto Rico	1949-1951	135	77877	1-18	Cancer, leukemia, lymphoma, HL, brain, bone, skin, kidney[e]	BCG	No association	Registry	Trial	Retrospective cohort	Trial based, trial arm according to birth year; original study of Snider 1978	25.15
Salonen, 1975[d]	Finland	1959-1968	972	972	<15	Cancer, leukemia, brain, eye, kidney, bone, other	Polio, BCG	No association	Registry	Record	Case-control	Mat: age, area, birth season; population-based without cancer; original study of Salonen 1976	25.86
Crispen, 1976[a,d]	USA (Chicago)	1957-1969	319	619907	<20	Cancer, leukemia	BCG [newborns]	↓ risk for cancer death in vaccinated group	Death certificate	Record	Retrospective cohort	Mortality rate; update of Rosenthal 1972	21.55
Salonen, 1976[a,d]	Finland	1959-1968	972	972	<15	Cancer, leukemia, brain, eye, kidney, bone, other tumors	Any, BCG	No association	Registry	Record	Case-control	[Mat: age, area, birth season;] population-based without cancer; update of Salonen 1975	22.45
Hofman, 1977	Europe	1965-1972	-	-	0-24	Leukemia	BCG	↓ mortality in countries with obligatory BCG	-	-	Ecological	Exc: without complete estimates; article in Polish	-2.63
Ambrosch, 1978	Austria	1954-1976	-	-	0-5	Leukemia	BCG	↓ leukemia mortality age 0-5 after BCG in newborns	-	-	Ecological	Exc: without complete estimates; protection rate	-0.63
Andersen, 1978[a,d]	Denmark (Copenhagen)	1943-1970	63	182	school children	HL	BCG	No association	Registry	Record	Case-control	[Mat: age, sex, socioeconomical status;] 1:3; population-based without cancer; Fisher´s exact test	19.33
Sinniah, 1978	Malaysia	1968-1975	38	16171	children	ALL	BCG	No association	Hospital	-	Ecological	Exc: without complete estimates; comparison of individual vaccination rates of cases & aggregated controls	11.54

Table continues

Supplementary Table 1. Continued

First Author, Year	Location	Study Years	No. of Cases	No. of Controls	Age Range	Cancer Sites	Vaccines [Early Age]	Results	Outcome	Exposure	Study Design	Comment	Study Quality[g]
Skegg, 1978	New Zealand (North & South Island)	1955-1973	-	-	13-23	HL, NHL, leukemia	BCG	No association	Registry	Registry	Ecological	Exc: without complete estimates; Adj: routine vaccination	10.0<br>4
Snider, 1978[a,d]	Puerto Rico	1949-1973	227	77745	1-18	Cancer, leukemia, lymphoma, HL, brain, bone, skin, kidney[e]	BCG	No association	Registry	Trial	Retrospective cohort	Trial based, trial arm according to birth year; update von Comstock, 1975	24.5<br>4
Farwell, 1979[a,d]	USA (Connecticut)	1956-1962	120	240	≤19	Central nervous system, glioma, medulloblastoma	Polio [prenatal]	↑ risk for medullablastoma; others: no association	Registry	Self-report	Case-control	[Mat: age, sex, area of residence;] original study of Farwell 1984	15.8<br>3
Nilsson, 1979	Sweden	1976-1977	37	194314	<2 (≤7)	Cancer	BCG [newborns]	No association	Registry	-	Ecological	Exc: without complete estimates; letter to editor	6.2<br>3
Neumann, 1980[a,d]	Germany	1972-1976	74	74	≤14	Cancer, leukemia	D, T, Polio, BCG, Pox	No association	Death certificate	Self-report	Case-control	Cancer death; [Mat: age, sex;] population-based; article in German	13.8<br>3
Kendrick, 1981[a,d]	USA (Georgia, Alabama)	1950-1977	852	33915	>5-<20 cancer; >5 sub-sites	Cancer, leukemia, multiple myeloma, lymphoma, HL, bone , brain, skin, kidney[f]	BCG	No association	-	Trial	Retrospective cohort	Trial-based; update of Comstock 1971	21.2<br>3
Gruffermann, 1982	USA (North Carolina)	1967-1976	33	99	0-14	Rhabdomyo-sarcoma	Any, Sma, MMR, DTP	↓ risk after smallpox vaccination; others: no association	Hospital	Self-report	Case-control	Exc: rare cancer; Mat: age, sex, race; Results from unmatched analyses; population-based	17.8<br>3
Sutherland, 1982[a,d]	England	1950-1979	28	54211	15-30	Leukemia	BCG	No association	Registry	Trial	Retrospective cohort	Mortality rate; trial-based; update / external validation of trial follow-up using registry data of MRC 1972	23.8<br>5
Farwell, 1984	USA (Connecticut)	1951-1974	180	360	0-19	Medulloblastoma	Polio [prenatal]	↑ risk for medullablastoma after prenatal exposure to polio vaccination	Registry	Self-report	Case-control	Exc: without complete estimates; Mat: age, sex, area of residence; information for prenatal vaccination for ~60% of cases and controls; update of Farwell 1979	18.6<br>3
Van Steensel-Moll 1985[d]	Netherlands	1973-1982	625	615	<15	Leukemia	Any [prenatal]	No association	Registry	Self-report	Case-control	Mat: age, sex, area; Adj: age, sex; population-based	21.8<br>5

Table continues

Supplementary Table 1. Continued

First Author, Year	Location	Study Years	No. of Cases	No. of Controls	Age Range	Cancer Sites	Vaccines [Early Age]	Results	Outcome	Exposure	Study Design	Comment	Study Quality[g]
Kneale, 1986[c,d]	England (Oxford)	1953-1977	12281	12281	0-15	Cancer, leukemia, lymphoma, cerebral tumor, neuroblastoma, osteosarcoma, Wilms tumor, other solid tumors	Any [0-1y], Sma, DT, P, Mea, Rub, Polio, BCG	↓ death risk for leukemia, wilms tumor, neuroblastoma, cerebral tumor & other solid tumors; ↓ death risk for cancer onset age 0-1 after vaccination age 0-1, onset age 2-4 after vaccination age 0-1 & 2-4, onset age 10-15 after vaccination age 10-15 & all ages; others no association	Hospital	Self-report	Case-control	Cancer death; Mat: sex, area, birth date (birth year, season); % risk; population-based child alive; Update of Stewart 1965	19.64
McKinney, 1987[d]	England (West Midlands, North West, Yorkshire)	1980-1983	234	468	1-15	Leukemia, ML, lymphoma	Any (T, D, P, Polio, Mea, triple, Sma)	↓ risk for leukemia in general; no association for myeloid leukemia, leukemia/ lymphoma, lymphoma	Registry	Self-report	Case-control	Mat: age, sex; hospital-based without cancer; original study of Hartley 1988	22.64
Hartley, 1988	England (West Midlands, North West, Yorkshire)	1980-1983	550	1110	2-14	Cancer	Any (T, D, P, Polio, R, Mea, Sma)	↓ risk for childhood cancer after 1+ vaccination compared to unvaccinated	Registry	Self-report	Case-control	Exc: rare vaccination; Mat: age, sex; hospital-based without cancer; never versus 1+; update of McKinney 1978	18.22
Gilman, 1989[b]	England, Scotland, Wales	1953-1979	8059	8059	Children	Cancer	Any [prenatal], gamma globulin, Inf, Polio, Sma, T, other	↑risk for childhood cancer after prenatal vaccination in general and after smallpox vaccination; others: no association	Hospital	Self-report	Case-control	Exc: rare vaccination; cancer death; Mat: sex, area, birth date (birth year, season); Adj: other factors; self-report supplied by general practitioner in 55% of cases and 59% of controls; population-based child alive; update of Stewart 1965 & Kneale 1986	21.25
Nishi, 1989[d]	Japan (Hokkaido)	1986-1987	63	126	0-14	Non-T cell ALL	BCG, Mea [<2y]	↓ risk	Hospital	Self-report	Case-control	Mat: age, sex, area; hospital-based	15.62
Buckley, 1994	USA	1982-1991	990 404	1636 440	0+	ALL	MMR, Sma, Mea	↓ risk for all & t-cell ALL after Sma with community controls; ↑ risk for ALL after MMR with cancer controls; others no association	Hospital	Self-report	Case-control	Exc: without complete estimates; Mat: age, race, area, family income; Children Cancer Group (CCG) study; population-based with & without cancer	21.05

Table continues

Supplementary Table 1. Continued

First Author, Year	Location	Study Years	No. of Cases	No. of Controls	Age Range	Cancer Sites	Vaccines [Early Age]	Results	Outcome	Exposure	Study Design	Comment	Study Quality[g]
Shu, 1995	USA, Canada	1982-1989	105	639	<15	Malignant germ-cell tumors	D, T, P, Polio, Mum, Mea, R, Sma	↓ risk after Sma; others: no association	Hospital	Self-report	Case-control	Exc: rare cancer; Adj: age, sex, gestational age, number livebirth (birth order), smoking during pregnancy, maternal education; population-based	22.46
Kaatsch, 1996	Germany (Lower Saxony)	1988-1993	348	433	0-14	Leukemia	Not specified	↓ vaccination rates in cases	Registry	Self-report	Case-control	Exc: without complete estimates; Mat: age, sex; population-based without & with cancer; original study Kaatsch 1998, Schüz 1999 & von Kries 2000	21.04
Bhatia, 1997	USA, Canada		459	683 3719	0-15	Langerhans cell histiocytosis (LCH)	D, P, T, Mea, R, Mum, Polio	↓ risk for multisystem LCH after D, T, P, Mea, Mum, R & Polio ↓ risk for single-system LCH after D, Polio, R, Mum & P; others no association	Record	Self-report	Case-control	Exc: rare cancer; Adj: age at questionnaire, area, birth year & order, annual household income, paternal education, maternal age; compared to community & cancer controls	20.05
Petridou, 1997[b,d]	Greece	1993-1994	153	300	0-14	Leukemia	DTP, BCG, viral (R, Mum, Mea, Hep)	No association	Hospital	Self-report	Case-control	Mat: age, sex, area; hospital-based without cancer	19.04
Kaatsch, 1998[d]	Germany (West Germany)	1992-1994	2358	2588	0-14	Leukemia	Number	↓ risk for leukemia for 0-3 & 4-6 versus >6 vaccinations; other cancer (NHL, CNS, neuro- & nephroblastoma, bone, soft-tissuesarcoma) not indicated	Registry	Self-report	Case-control	Adj: socioeconomic status, urban-rural status; Mat: age, sex, area; population-based; update Kaatsch 1996 & original study Schüz 1999 & von Kries 2000	21.63
Dockerty, 1999[d]	New Zealand	1990-1993	121	121	0-14	Leukemia	Any, number, routine, DTP, DT, BCG, Hep & other [>3m]; MMR & Mea [>9m]; Polio [>6m]; R [>15m]	↓ risk for leukemia after 1-4 different vaccinations (adj. only for age & sex); others no association	Registry	Record (parent held)	Case-control	Adj: age, sex; Mat: age, sex; latency considered; population-based	24.25
Groves, 1999[d]	USA (IL, IN, IA, MI, MN, NJ, OH, PA, WI)	1989-1993	439	439	0-14	ALL	DTP, D, T, Polio, MMR, Hib	↓ risk for ALL after Hib (conjug.); others no association	-	Record	Case-control	Adj: age, sex, race, birth year, day care attendance, parental education, family income; Mat: age, race, telephone number; population-based	18.04

Table continues

Supplementary Table 1. Continued

First Author, Year	Location	Study Years	No. of Cases	No. of Controls	Age Range	Cancer Sites	Vaccines [Early Age]	Results	Outcome	Exposure	Study Design	Comment	Study Quality[g]
Schüz, 1999[d]	Germany	1980-1994	1010	1010	0-14	AL, ALL	Number (D, T, P, Polio, Mum, Mea, R, Sma, Men, routine)	↑ risk for leukemia for 0-3 & 4-6 versus >6 vaccinations	Registry	Self-report	Case-control	Adj: socioeconomic status; Mat: sex, birth year; population-based non-diseased; update Kaatsch 1996 & 1998	22.24
Auvinen, 2000[d]	Finland	1985-1987	77	113923	0-14	Leukemia, ALL	Hib (PRP-D) [3, 4, 6 & 14/18m]	No association	Registry	Trial	Retrospective cohort	Adj: other vaccinations; Trial-based	35.46
Von Kries, 2000[d]	Germany (Lower Saxony)	1988-1993	420	613	0-15	Cancer, leukemia, tumors	BCG [newborns]	No association	Registry	Self-report	Case-control	Adj: age, sex; Mat: age, sex; population-based without cancer; power only 50%; update Kaatsch 1996 & 1998 & Schüz 1999	22.25
Krone, 2003[d]	UK, Bulgaria, Italy, Germany, Estonia, Israel, Austria, France	1994-1997	603	627	0+	Malignant melanoma	BCG, Sma, Inf	↓ risk for for melanoma after BCG, smallpox or both in total & in several single countries	Hospital	Self-report (some cards)	Case-control	Adj: age, sex, race, study center, skin type, pigmented naevi, sunburns, freckling index; population-based	24.46
Frentzel-Beyme, 2004[d]	Austria	1978-1988	88	208	8-25	Osteo- & Ewing-sarcoma, other bone tumors	D, T, P, Polio, BCG, Chi, vaccination reaction	↓ risk after repeated pertussis vaccination in girls univariate; others no association	Registry	Self-report	Case-control	Mat: age, sex; population-based, hospital-based	21.05
Ma, 2005[d]	USA (California)	1995-2002	323	409	0-14	Leukemia, ALL	DPT, Polio, MMR, Hep [<1y], Hib	↓ risk for leukemia & ALL after Hib vaccination; others no association	Registry	Vaccination card	Case-control	Adj: birth weight, day care attendance, family income, maternal education; Mat: age, sex, mother´s race, hispanic status; population-based	23.25
Mallol-Mesnard, 2007[b,d]	France	2003-2004	776	1681	<15	AL, ALL, AML	Number [6m]; BCG [newborns]; D, T, P, Hep, Hib, Pne, Men & Polio [<6m]; Mum, Mea & R [1y]	↑ risk of AML after 1-2 vaccinations <6 months compared to ≥4 vaccinations; others no association	Registry	Vaccination card	Case-control	Adj: age, sex, birth order, maternal and paternal educational level, degree of urbanization; Mat: age, sex; population-based	27.86
Daniels, 2008	USA, Canada	1999-2003	521	517	<16	Wilms tumor	Any [prenatal]	No association	Hospital	Self-report	Case-control	Exc: rare vaccination; Mat: age, area; Adj: age, area, sex, maternal education, household income; population based	22.25
MacArthur, 2008[d]	Canada	1990-1994	399	399	0-14	Leukemia, ALL	D, T, P, Polio, Mum, Mea, R, BCG, Hep, other	No association	Registry	Vaccination card	Case-control	Adj: race, family income, maternal education & age at birth, number of residences since birth; Mat: age, sex area; population-based	26.65

Table continues

Supplementary Table 1. Continued

First Author, Year	Location	Study Years	No. of Cases	No. of Controls	Age Range	Cancer Sites	Vaccines [Early Age]	Results	Outcome	Exposure	Study Design	Comment	Study Quality[g]
Villumsen, 2009	Denmark	1965-1976	71	2073	5-35	Lymphoma, NHL, HL, leukemia	BCG, Sma	↓ lymphoma risk after BCG; others: no association	Registry	Vaccination card	Retrospective case-cohort	Adj: day care, family social class; register-based; Sub-cohort; update of Danish data in Waaler 1970	27.68
Pagaoa, 2011	USA (Texas)	1995-2006	2800	11200	2-17	Cancer, ALL, NHL, medullablastoma	DTP, Polio, MMR, Chi, Hep, Hib, combination	↓ risk for all cancers & ALL after Hib & for ALL after combined vaccination by region; ↓ risk for all cancers & ALL after Hep & for ALL after IPV, Hep & combined vaccination, ↑ risk for medullablastoma after Hib	Registry	Registry	Ecological	Adj: age, sex, race, birth weight, birth year, birth type, birth order, premature birth, maternal education, maternal marital status, prior births, diabetes, preterm labor, tobacco use, and alcohol use, mother age at birth; Mat: sex, birth year; 1:4; population- based without cancer	13.75
Qu, 2014[b]	China	1983-1990	17	64997	0-30	Hepatocellular carcinoma, primary liver cancer	Hep	↓ risk for hepatocellular carcinoma	Registry	Trial	Retrospective	Exc: rare cancer; Adj: cluster; trial-based; mortality rates combined with incidence rates	32.47
Sankaran 2016	USA	1982-1988	322	322	0-20	Rhabdomyo-sarcoma	DTP, Polio, MMR, completed vs. non-completed	↓ risk for complete vaccination; ↑ risk for incomplete DTP to complete DTP; others no association	Trial	Self-report	Case-control	Exc: rare cancer; Adj: family income, maternal education; Mat: age, sex, race; population-based; no controls for 8% cases	21.24
Soegaard, 2017	Denmark	1981-2008	490	1224914	0-14	ALL	DTPolio [5, 6, 16m], P (<3, 10m], MMR, Hib [3-16m], routine	No association	Registry	Registry	Retrospective cohort	Adj: sex, race, birth weight, year, order & mode, other vaccination, gestational age; down syndrome excluded; latency considered; register-	37.08
Figueroa, 2019	Costa Rica	1995-2003	240	578	1-15	ALL	Routine vaccination	↓ risk for ALL	Registry	Self-report	Case-control	Adj: sex, birth year, SES	21.66

= Not included in meta-analysis
= Included in meta-analysis with cancer cases
= Included in meta-analysis with cancer deaths

Abbreviations: Adj, Adjustment; AL, Acute leukemia; ALL, Acute lymphoblastic leukemia; AML, Acute myeloid leukemia; BCG, Bacillus Calmette–Guérin (vole bacillus; tuberculosis); Chi, Chicken pox (varicella zoster); Cho, Cholera; D, Diphtheria; DT, Diphtheria-Tetanus; DTP, Diphtheria-Tetanus-Pertussis/Whooping cough; DTPolio, Diphtheria-Tetanus-Poliomyelitis; Exc, Exclusion for meta-analysis; Hep, Hepatitis; Hib, Haemophilus influenzae type b; Inf, Influenza; HL, Hodgkin lymphoma; m, months; Mat, Matching; Mea, Measles; Men, Meningococcus; MMR, Mumps-Measles-Rubella; MRC, Medical Research Council; Mum, Mumps; NHL, Non-Hodgkin lymphoma; Pne, Pneumococcus; Sma, Smallpox; Typ, Typhoid; y, years; Yel, Yellow fever.

[a] Calculation of crude ORs .
[b] Partly calculation of crude ORs not included in meta-analysis
[c] Calculation of crude ORs taking individual matching into account.
[d] Study included in meta-analysis.
[e] Cancer, leukemia, lymphoma, HL, nervous system, bone, kidney, ovary, male genitalia, skin, bladder, salivary glands, mouth, esophagus, stomach, colon, liver, larynx, lungs, breast, cervix, uterus, other endocrine organs, connective tissue.
[f] Cancer, leukemia, multiple myeloma, lymphatic tissue, HL, brain, other nervous system, bone, kidney, bladder, other urinary organs, ovary, prostate, other female/male genital organs, eye, skin, other skin, breast, bronchus & lung, cervix, connective tissue, esophagus, large intestine, larynx, liver, mouth, nose, other digestive organs, other endocrine glands, pancreas, peritoneum, rectum, salivary gland, stomach, thyroid, tonsil, uterus.
[g] Study quality with detailed quality score (-6 to 45 points) and Newcastle-Ottawa Scale (0 to 9 points).
