# Supplementary material for: Vaccination and the Risk of Childhood Cancer—A Systematic Review and Meta-Analysis
Source: Front Oncol. 2021 Jan 22;10:610843. doi: 10.3389/fonc.2020.610843 (PMC7862764; doi:10.3389/fonc.2020.610843)
Supplement: Supplementary file 2 [file DataSheet_2.pdf]

Supplementary Table 2A. Estimates of cohort studies included in the systematic review

Reference	Age Range	Exposure	Outcome	Comments	Cancer Site	Vaccine	Subsample	Model (Estimate / Ref)	No. of cases (Estimate / Ref)	No. of Controls or Expected (Estimate / Ref)	OR	95%CI
Davignon, 1970[a,c]	<15	Registry	Registry	Mortality rate; irrelevant errors in table 1 corrected by Davignon 1971	Leukemia death	BCG	1960-1963	Ever / Never	96 / 191	407804 / 341669	0.42	(0.33 to 0.54)[a,d]
					Leukemia death	BCG	1960	Ever / Never	20 / 52	93820 / 88698	0.36	(0.22 to 0.61)[a]
					Leukemia death	BCG	1961	Ever / Never	22 / 46	99508 / 86754	0.42	(0.25 to 0.69)[a]
					Leukemia death	BCG	1962	Ever / Never	33 / 44	105257 / 83806	0.60	(0.38 to 0.94)[a]
					Leukemia death	BCG	1963	Ever / Never	21 / 49	109219 / 82411	0.32	(0.19 to 0.54)[a]
Comstock, 1971[a]	>5	Trial	Hospital	Exc: overlapping population; [Adj: age, race;] trial-based; original study of Kendrick 1981	Hodgkin lymphoma	BCG		Ever / Never	4 / 4	16909 / 17850	1.06	(0.26 to 4.22)[a]
					Leukemia	BCG		Ever / Never	6 / 6	16907 / 17848	1.06	(0.34 to 3.27)[a]
					Lymphoma	BCG		Ever / Never	4 / 6	16909 / 17848	0.70	(0.20 to 2.49)[a]
MRC, 1972[a,c]	15-30	Trial	Follow-Up	Mortality rate; outcome incidence & cancer deaths; trial-based; original study of Sutherland 1982	Cancer death	BCG		Ever / Never	7 / 4	13591 / 12863	1.66	(0.48 to 5.66)[a,d]
					Cancer death	BCG (Vole Bacillus)		Ever / Never	3 / 4	5814 / 12863	1.66	(0.37 to 7.42)[a]
					Leukemia or Lymphoma death	BCG		Ever / Never	6 / 8	13592 / 12859	0.71	(0.25 to 2.05)[a]
					Leukemia or Lymphoma death	BCG (Vole Bacillus)		Ever / Never	1 / 8	5816 / 12859	0.28	(0.03 to 2.21)[a]
Rosenthal, 1972[a]	0-6	Records	Registry	Exc: overlapping population; mortality rate; immigration not factored; [Adj: age race;] original study of Crispen 1976	Leukemia death	BCG		Ever / Never	1 / 21	54413 / 172975	0.15	(0.02 to 1.13)[a]
					Leukemia death	BCG	Death age <1	Ever / Never	0 / 0	7820 / 21901		
					Leukemia death	BCG	Death age 1-3	Ever / Never	0 / 8	26482 / 68816		
					Leukemia death	BCG	Death age 4-6	Ever / Never	1 / 13	20111 / 82248	0.31	(0.04 to 2.41)[a]
Heinonen, 1973[a,c]	0-4	Self-report	Records	[Adj: race;] prenatal vaccination	Cancer	Polio	Vaccination in pregnancy	Ever / Never	14 / 10	18328 / 32545	2.49	(1.10 to 5.60)[a]
					Cancer	Polio	Vaccination in pregnancy (first 3 months)	Ever / Never	6 / 10	3846 / 32545	5.08	(1.84 to 13.98)[a]
					Cancer	Polio	Vaccination in pregnancy (first 4 months)	Ever / Never	9 / 10	6825 / 32545	4.29	(1.74 to 10.57)[a]
					Cancer	Polio	Vaccination in pregnancy, diagnosis age <1	Ever / Never	7 / 6			
					Cancer	Polio	Vaccination in pregnancy, diagnosis age 1-4	Ever / Never	7 / 2			
					Cancer	Polio	Vaccination in pregnancy, diagnosis stillborns	Ever / Never	0 / 2			
					Cancer, other than leukemia and neural	Polio	Vaccination in pregnancy	Ever / Never	3 / 5	18339 / 32550	1.06	(0.25 to 4.46)[a]
					Cancer, other than leukemia and neural	Polio	Vaccination in pregnancy, diagnosis age <1	Ever / Never	1 / 3			
					Cancer, other than leukemia and neural	Polio	Vaccination in pregnancy, diagnosis age 1-4	Ever / Never	2 / 0			
					Cancer, other than leukemia and neural	Polio	Vaccination in pregnancy, diagnosis stillborns	Ever / Never	0 / 2			
					Leukemia	Polio	Vaccination in pregnancy	Ever / Never	4 / 4	18338 / 32551	1.78	(0.44 to 7.10)[a,d]
					Leukemia	Polio	Vaccination in pregnancy, diagnosis age <1	Ever / Never	0 / 2			

Supplementary Table 2. Continued

Reference	Age Range	Exposure	Outcome	Comments	Cancer Site	Vaccine	Subsample	Model (Estimate / Ref)	No. of cases (Estimate / Ref)	No. of Controls or Expected (Estimate / Ref)	OR	95%CI
					Leukemia	Polio	Vaccination in pregnancy, diagnosis age 1-4	Ever / Never	4 / 2			
					Leukemia	Polio	Vaccination in pregnancy, diagnosis stillborns	Ever / Never	0 / 0			
					Neural tumors	Polio	Vaccination in pregnancy	Ever / Never	7 / 1	18335 / 32554	12.43	(1.53 to 101.03)[a]
					Neural tumors	Polio	Vaccination in pregnancy, diagnosis age <1	Ever / Never	6 / 1			
					Neural tumors	Polio	Vaccination in pregnancy, diagnosis age 1-4	Ever / Never	1 / 0			
					Neural tumors	Polio	Vaccination in pregnancy, diagnosis stillborns	Ever / Never	0 / 0			
					Neural tumors	Polio + Influenza	Vaccination in pregnancy	Ever / Never	1 / -			
Comstock, 1975[a,c]	1-18	Trial	Registry	Trial based, trial arm according to birth year; original study of Snider 1978	Bladder	BCG		Ever / Never	2 / 0	50632 / 27338		
					Bone	BCG		Ever / Never	3 / 1	50631 / 27337	1.62	(0.17 to 15.57)[a]
					Brain tumor	BCG		Ever / Never	7 / 5	50627 / 27333	0.76	(0.24 to 2.38)[a,d]
					Breast	BCG		Ever / Never	3 / 0	50631 / 27338		
					Cancer	BCG		Ever / Never	98 / 37	50536 / 27331	1.43	(0.98 to 2.09)[a]
					Cancer, other than leukemia and lymphoma	BCG		Ever / Never	69 / 27	50565 / 27311	1.38	(0.88 to 2.15)[a]
					Cancer, other than leukemia and lymphoma	BCG	Trial entry age 1-3	Ever / Never	6 / 2	50628 / 27336	1.62	(0.33 to 8.03)[a]
					Cancer, other than leukemia and lymphoma	BCG	Trial entry age 4-6	Ever / Never	5 / 3	50629 / 27335	0.90	(0.22 to 3.77)[a]
					Cancer, other than leukemia and lymphoma	BCG	Trial entry age 7-9	Ever / Never	7 / 6	50627 / 27332	0.63	(0.21 to 1.87)[a]
					Cancer, other than leukemia and lymphoma	BCG	Trial entry age 10-12	Ever / Never	22 / 7	50612/ 27331	1.70	(0.72 to 3.97)[a]
					Cancer, other than leukemia and lymphoma	BCG	Trial entry age 13-15	Ever / Never	17 / 8	50617 / 27330	1.15	(0.50 to 2.66)[a]
					Cancer, other than leukemia and lymphoma	BCG	Trial entry age 16-18	Ever / Never	12 / 1	50622 / 27337	6.48	(0.84 to 49.84)[a]
					Cancer death	BCG		Ever / Never	40 / 13	50594 / 27325	1.66	(0.89 to 3.11)[a]
					Cervix	BCG		Ever / Never	21 / 10	50613 / 27328	1.13	(0.53 to 2.41)[a]
					Colon	BCG		Ever / Never	1 / 1	50633 / 27337	0.54	(0.03 to 8.63)[a]
					Connective tissue	BCG		Ever / Never	4 / 3	50630 / 27335	0.72	(0.16 to 3.22)[a]
					Endocrine organs	BCG		Ever / Never	1 / 0	50633 / 27338		
					Esophagus	BCG		Ever / Never	1 / 0	50633 / 27338		
					Hodgkin lymphoma	BCG		Ever / Never	9 / 0	50625 / 27338		
					Hodgkin lymphoma	BCG	Trial entry age 1-3	Ever / Never	1 / 0	50633 / 27338		
					Hodgkin lymphoma	BCG	Trial entry age 4-6	Ever / Never	1 / 0	50633 / 27338		
					Hodgkin lymphoma	BCG	Trial entry age 7-9	Ever / Never	2 / 0	50632 / 27338		
					Hodgkin lymphoma	BCG	Trial entry age 10-12	Ever / Never	4 / 0	50630 / 27338		
					Hodgkin lymphoma	BCG	Trial entry age 13-15	Ever / Never	0 / 0	50634 / 27338		
					Hodgkin lymphoma	BCG	Trial entry age 16-18	Ever / Never	1 / 0	50633 / 27338		
					Kidney	BCG		Ever / Never	3 / 1	50631 / 27337	1.62	(0.17 to 15.57)[a]
					Larynx	BCG		Ever / Never	2 / 0	50632 / 27338		
					Leukemia	BCG		Ever / Never	12 / 9	50622 / 27329	0.72	(0.30 to 1.71)[a]

Table continues

Supplementary Table 2. Continued

Reference	Age Range	Exposure	Outcome	Comments	Cancer Site	Vaccine	Subsample	Model (Estimate / Ref)	No. of cases (Estimate / Ref)	No. of Controls or Expected (Estimate / Ref)	OR	95%CI
					Leukemia	BCG	Trial entry age 1-3	Ever / Never	0 / 1	50634 / 27337		
					Leukemia	BCG	Trial entry age 4-6	Ever / Never	1 / 2	50633 / 27336	0.27	(0.02 to 2.98)[a]
					Leukemia	BCG	Trial entry age 7-9	Ever / Never	2 / 3	50632 / 27335	0.36	(0.06 to 2.15)[a]
					Leukemia	BCG	Trial entry age 10-12	Ever / Never	4 / 2	50630 / 27336	1.08	(0.20 to 5.90)[a]
					Leukemia	BCG	Trial entry age 13-15	Ever / Never	5 / 0	50629 / 27338		
					Leukemia	BCG	Trial entry age 16-18	Ever / Never	0 / 1	50634 / 27337		
					Liver	BCG		Ever / Never	1 / 0	50633 / 27338		
					Lung	BCG		Ever / Never	1 / 0	50633 / 27338		
					Lymphoma	BCG		Ever / Never	8 / 1	50626 / 27337	4.32	(0.54 to 34.54)[a]
					Lymphoma	BCG	Trial entry age 1-3	Ever / Never	0 / 1	50634 / 27337		
					Lymphoma	BCG	Trial entry age 4-6	Ever / Never	0 / 0	50634 / 27338		
					Lymphoma	BCG	Trial entry age 7-9	Ever / Never	1 / 0	50633 / 27338		
					Lymphoma	BCG	Trial entry age 10-12	Ever / Never	4 / 0	50630 / 27338		
					Lymphoma	BCG	Trial entry age 13-15	Ever / Never	1 / 0	50633 / 27338		
					Lymphoma	BCG	Trial entry age 16-18	Ever / Never	2 / 0	50632 / 27338		
					Male genitalia	BCG		Ever / Never	1 / 0	50633 / 27338		
					Mouth	BCG		Ever / Never	0 / 1	50634 / 27337		
					Ovary	BCG		Ever / Never	3 / 1	50631 / 27337	1.62	(0.17 to 15.57)[a]
					Salivary gland	BCG		Ever / Never	1 / 0	50633 / 27338		
					Skin	BCG		Ever / Never	4 / 1	50630 / 27337	2.16	(0.24 to 19.32)[a]
					Stomach	BCG		Ever / Never	3 / 0	50631 / 27338		
					Thyroid	BCG		Ever / Never	5 / 2	50629 / 27336	1.35	(0.26 to 6.96)[a]
					Uterus	BCG		Ever / Never	2 / 1	50632 / 27337	1.08	(0.10 to 11.91)[a]
Crispen, 1976[a,c]	<20	Records	Death certificates	Mortality rate; update of Rosenthal 1972	Cancer death	BCG	Death age <1	Ever / Never	0 / 7	85356 / 534863		
					Cancer death	BCG	Death age 1-4	Ever / Never	8 / 77	85348 / 534793	0.65	(0.31 to 1.35)[a]
					Cancer death	BCG	Death age 5-9	Ever / Never	4 / 81	85352 / 534789	0.31	(0.11 to 0.84)[a]
					Cancer death	BCG	Death age 10-14	Ever / Never	0 / 67	85356 / 534803		
					Cancer death	BCG	Death age 15-20	Ever / Never	1 / 74	85355 / 534796	0.08	(0.01 to 0.61)[a]
					Cancer death	BCG	Death, female	Ever / Never	8 / 138	42928 / 265430	0.36	(0.18 to 0.73)[a]
					Cancer death	BCG	Death, male	Ever / Never	5 / 170	42415 / 269134	0.19	(0.08 to 0.45)[a]
					Cancer death	BCG	Death, total	Ever / Never	13 / 306	85343 / 534564	0.27	(0.15 to 0.46)[a,d]
					Leukemia death	BCG	Death age <1	Ever / Never	0 / 2	85356 / 534868		
					Leukemia death	BCG	Death age 1-4	Ever / Never	5 / 34	85351 / 534836	0.92	(0.36 to 2.36)[a]
					Leukemia death	BCG	Death age 5-9	Ever / Never	1 / 36	85355 / 534834	0.17	(0.01 to 1.27)[a]
					Leukemia death	BCG	Death age 10-14	Ever / Never	0 / 18	85356 / 534852		
					Leukemia death	BCG	Death age 15-20	Ever / Never	0 / 16	85356 / 534854		
					Leukemia death	BCG	Death, total	Ever / Never	6 / 106	85350 / 534764	0.35	(0.16 to 0.81)[a,d]
					Other than leukemia death	BCG	Death age <1	Ever / Never	0 / 5	85356 / 534865		
					Other than leukemia death	BCG	Death age 1-4	Ever / Never	3 / 43	85353 / 534827	0.44	(0.14 to 1.41)[a]

Table continues

Supplementary Table 2. Continued

Reference	Age Range	Exposure	Outcome	Comments	Cancer Site	Vaccine	Subsample	Model (Estimate / Ref)	No. of cases (Estimate / Ref)	No. of Controls or Expected (Estimate / Ref)	OR	95%CI
					Other than leukemia death	BCG	Death age 5-9	Ever / Never	3 / 45	85353 / 534825	0.42	(0.13 to 1.34)[a]
					Other than leukemia death	BCG	Death age 10-14	Ever / Never	0 / 49	85356 / 534821		
					Other than leukemia death	BCG	Death age 15-20	Ever / Never	1 / 58	85355 / 534812	0.11	(0.01 to 0.78)[a]
					Other than leukemia death	BCG	Death, total	Ever / Never	7 / 200	85349 / 534670	0.22	(0.10 to 0.47)[a]
Snider, 1978[a,c]	1-18	Trial	Registry	Trial based, trial arm according to birth year; update von Comstock, 1975	Bladder	BCG		Ever / Never	2 / 0	50632 / 27338		
					Bone tumor	BCG		Ever / Never	4 / 1	50630 / 27337	2.16	(0.24 to 19.32)[a,d]
					Breast	BCG		Ever / Never	7 / 5	50627 / 27333	0.76	(0.24 to 2.38)[a]
					Cancer	BCG		Ever / Never	150 / 77	50484 / 27261	1.05	(0.80 to 1.39)[a,d]
					Cancer, other than leukemia and lymphoma	BCG	Trial entry age 1-3	Ever / Never	7 / 3	5525 / 2816	1.19	(0.31 to 4.60)[a]
					Cancer, other than leukemia and lymphoma	BCG	Trial entry age 4-6	Ever / Never	11 / 6	7534 / 3151	0.77	(0.28 to 2.08)[a]
					Cancer, other than leukemia and lymphoma	BCG	Trial entry age 7-9	Ever / Never	21 / 12	10877 / 6639	1.07	(0.53 to 2.17)[a]
					Cancer, other than leukemia and lymphoma	BCG	Trial entry age 10-12	Ever / Never	40 / 21	13823 / 7543	1.04	(0.61 to 1.76)[a]
					Cancer, other than leukemia and lymphoma	BCG	Trial entry age 13-15	Ever / Never	25 / 19	8931 / 5294	0.78	(0.43 to 1.42)[a]
					Cancer, other than leukemia and lymphoma	BCG	Trial entry age 16-18	Ever / Never	14 / 4	3826 / 1830	1.67	(0.55 to 5.09)[a]
					Cancer, unknown site	BCG		Ever / Never	0 / 1	50634 / 27337		
					Colon/rectum	BCG		Ever / Never	3 / 3	50631 / 27335	0.54	(0.11 to 2.68)[a]
					Connective tissue	BCG		Ever / Never	4 / 3	50630 / 27335	0.72	(0.16 to 3.22)[a]
					Endocrine organs	BCG		Ever / Never	1 / 0	50633 / 27338		
					Esophagus	BCG		Ever / Never	1 / 0	50633 / 27338		
					Extrahepatic bile duct	BCG		Ever / Never	0 / 1	50634 / 27337		
					Hodgkin lymphoma	BCG		Ever / Never	9 / 2	50625 / 27336	2.43	(0.52 to 11.25)[a,d]
					Hodgkin lymphoma	BCG	Trial entry age 1-3	Ever / Never	1 / 0	5531 / 2819		
					Hodgkin lymphoma	BCG	Trial entry age 4-6	Ever / Never	1 / 0	7544 / 3157		
					Hodgkin lymphoma	BCG	Trial entry age 7-9	Ever / Never	3 / 0	10895 / 6651		
					Hodgkin lymphoma	BCG	Trial entry age 10-12	Ever / Never	3 / 1	13860 / 7563	1.64	(0.17 to 15.74)[a]
					Hodgkin lymphoma	BCG	Trial entry age 13-15	Ever / Never	1 / 1	8955 / 5312	0.59	(0.04 to 9.49)[a]
					Hodgkin lymphoma	BCG	Trial entry age 16-18	Ever / Never	0 / 0	3840 / 1834		
					Kidney	BCG		Ever / Never	3 / 2	50631 / 27336	0.81	(0.14 to 4;85)[a,d]
					Larynx	BCG		Ever / Never	2 / 1	50632 / 27337	1.08	(0.10 to 11.91)[a]
					Leukemia	BCG		Ever / Never	14 / 9	50620 / 27329	0.84	(0.36 to 1.94)[a,d]
					Leukemia	BCG	Trial entry age 1-3	Ever / Never	0 / 1	5532 / 2818		
					Leukemia	BCG	Trial entry age 4-6	Ever / Never	2 / 2	7543 / 3155	0.42	(0.06 to 2.97)[a]
					Leukemia	BCG	Trial entry age 7-9	Ever / Never	3 / 3	10895 / 6648	0.61	(0.12 to 3.02)[a]
					Leukemia	BCG	Trial entry age 10-12	Ever / Never	5 / 2	13858 / 7562	1.36	(0.26 to 7.03)[a]
					Leukemia	BCG	Trial entry age 13-15	Ever / Never	4 / 0	8952 / 5313		
					Leukemia	BCG	Trial entry age 16-18	Ever / Never	0 / 1	3840 / 1833		

Table continues

Reference	Age Range	Exposure	Outcome	Comments	Cancer Site	Vaccine	Subsample	Model (Estimate / Ref)	No. of cases (Estimate / Ref)	No. of Controls or Expected (Estimate / Ref)	OR	95%CI
					Liver	BCG		Ever / Never	1 / 0	50633 / 27338		
					Lung	BCG		Ever / Never	2 / 0	50632 / 27338		
					Lymphoma	BCG		Ever / Never	9 / 1	50625 / 27337	4.86	(0.62 to 38.36)[a,d]
					Lymphoma	BCG	Trial entry age 1-3	Ever / Never	0 / 1	5532 / 2818		
					Lymphoma	BCG	Trial entry age 4-6	Ever / Never	0 / 0	7545 / 3157		
					Lymphoma	BCG	Trial entry age 7-9	Ever / Never	1 / 0	10897 / 6651		
					Lymphoma	BCG	Trial entry age 10-12	Ever / Never	4 / 0	13859 / 7564		
					Lymphoma	BCG	Trial entry age 13-15	Ever / Never	3 / 0	8953 / 5313		
					Lymphoma	BCG	Trial entry age 16-18	Ever / Never	1 / 0	3839 / 1834		
					Male genitalia	BCG		Ever / Never	1 / 1	50633 / 27337	0.54	(0.03 to 8.63)[a]
					Mouth	BCG		Ever / Never	0 / 1	50634 / 27337		
					Nervous system	BCG		Ever / Never	9 / 7	50635 / 27331	0.69	(0.26 to 1.86)[a]
					Ovary	BCG		Ever / Never	4 / 1	50630 / 27337	2.16	(0.24 to 19.32)[a]
					Salivary gland	BCG		Ever / Never	4 / 0	50629 / 27338		
					Skin	BCG		Ever / Never	8 / 6	50626 / 27332	0.72	(0.25 to 2.07)[a,d]
					Small intestine	BCG		Ever / Never	2 / 0	50632 / 27338		
					Stomach	BCG		Ever / Never	5 / 1	50629 / 27337	2.70	(0.32 to 23.11)[a]
					Thyroid	BCG		Ever / Never	8 / 3	50626 / 27335	1.44	(0.38 to 5.43)[a]
					Uterus	BCG		Ever / Never	47 / 28	50587 / 27310	0.91	(0.57 to 1.45)[a]
Kendrick, 1981[a,c]	>5	Trial		Trial-based; update of Comstock 1971	Bladder	BCG		Ever / Never	17 / 11	16896 / 17843	1.63	(0.76 to 3.49)[a]
					Bone tumor	BCG		Ever / Never	5 / 4	16908 / 17850	1.32	(0.35 to 4.92)[a,d]
					Brain tumor	BCG		Ever / Never	9 / 5	16904 / 17849	1.90	(0.64 to 5.67)[a,d]
					Breast	BCG		Ever / Never	79 / 81	16834 / 17773	1.03	(0.75 to 1.40)[a]
					Bronchus and lung	BCG		Ever / Never	34 / 36	16879 / 17818	1.0	(0.62 to 1.59)[a]
					Cancer	BCG	All cases	Ever / Never	429 / 423	16484 / 17431	1.07	(0.94 to 1.23)[a,d]
					Cancer	BCG	White males, diagnosis all ages	Ever / Never	99 / 106	16817 / 17748	0.99	(0.75 to 1.30)[a]
					Cancer	BCG	White males, diagnosis age <20	Ever / Never	8 / 12	16905 / 17842	0.70	(0.29 to 1.72)[a]
					Cancer	BCG	White females, diagnosis all ages	Ever / Never	254 / 220	16659 / 17634	1.22	(1.02 to 1.47)[a]
					Cancer	BCG	White females, diagnosis age <20	Ever / Never	14 / 22	16899 / 17832	0.67	(0.34 to 1.31)[a]
					Cancer	BCG	Nonwhite males, diagnosis all ages	Ever / Never	23 / 24	16890 / 17830	1.01	(0.57 to 1.79)[a]
					Cancer	BCG	Nonwhite males, diagnosis age <20	Ever / Never	5 / 4	16908 / 17850	1.32	(0.35 to 4.92)[a]
					Cancer	BCG	Nonwhite females, diagnosis all ages	Ever / Never	53 / 73	16860 / 17781	0.77	(0.54 to 1.09)[a]
					Cancer	BCG	Nonwhite females, diagnosis age <20	Ever / Never	10 / 19	16903 / 17835	0.56	(0.26 to 1.19)[a]
					Cancer, unknown site	BCG		Ever / Never	21 / 13	16892 / 17841	1.71	(0.85 to 3.41)[a]
					Cancer, two sites	BCG		Ever / Never	7 / 7	16906 / 17847	1.06	(0.37 to 3.01)[a]
					Cervix	BCG		Ever / Never	39 / 44	16874 / 17810	0.94	(0.61 to 1.44)[a]
					Connective tissue	BCG		Ever / Never	3 / 7	16910 / 17847	0.45	(0.12 to 1.75)[a]

Table continues

Supplementary Table 2. Continued

Reference	Age Range	Exposure	Outcome	Comments	Cancer Site	Vaccine	Subsample	Model (Estimate / Ref)	No. of cases (Estimate / Ref)	No. of Controls or Expected (Estimate / Ref)	OR	95%CI
					Esophagus	BCG		Ever / Never	1 / 3	16912 / 17851	0.35	(0.04 to 3.38)[a]
					Eye	BCG		Ever / Never	1 / 1	16912 / 17853	1.06	(0.07 to 16.88)[a]
					Hodgkin lymphoma	BCG		Ever / Never	7 / 5	16906 / 17849	1.48	(0.47 to 4.66)[a,d]
					Kidney	BCG		Ever / Never	9 / 2	16904 / 17852	4.75	(1.03 to 22.00)[a,d]
					Large intestine	BCG		Ever / Never	24 / 40	16889 / 17814	0.63	(0.38 to 1.05)[a]
					Larynx	BCG		Ever / Never	4 / 6	16909 / 17848	0.70	(0.20 to 2.49)[a]
					Leukemia	BCG		Ever / Never	20 / 11	16893 / 17843	1.92	(0.92 to 4.01)[a,d]
					Liver	BCG		Ever / Never	1 / 4	16912 / 17850	0.26	(0.03 to 2.36)[a]
					Lymphoma	BCG		Ever / Never	8 / 3	16905 / 17851	2.82	(0.75 to 10.62)[a,d]
					Mouth	BCG		Ever / Never	7 / 8	16906 / 17846	0.92	(0.33 to 2.55)[a]
					Multiple myeloma	BCG		Ever / Never	2 / 2	16911 / 17852	1.06	(0.15 to 7.50)[a]
					Nose	BCG		Ever / Never	3 / 1	16910 / 17853	3.17	(0.33 to 30.45)[a]
					Other digestive organs	BCG		Ever / Never	3 / 2	16910 / 17852	1.58	(0.26 to 9.48)[a]
					Other endocrine glands	BCG		Ever / Never	1 / 1	16912 / 17853	1.06	(0.07 to 16.88)[a]
					Other female genital organs	BCG		Ever / Never	4 / 3	16909 / 17851	1.41	(0.31 to 6.29)[a]
					Other male genital organs	BCG		Ever / Never	1 / 4	16912 / 17850	0.26	(0.03 to 2.36)[a]
					Other nervous system	BCG		Ever / Never	1 / 1	16912 / 17853	1.06	(0.07 to 16.88)[a]
					Other skin cancers	BCG		Ever / Never	29 / 31	16884 / 17823	0.99	(0.59 to 1.64)[a]
					Other urinary organs	BCG		Ever / Never	1 / 1	16912 / 17853	1.06	(0.07 to 16.88)[a]
					Ovary	BCG		Ever / Never	17 / 12	16896 / 17842	1.50	(0.71 to 3.13)[a]
					Pancreas	BCG		Ever / Never	8 / 9	16905 / 17845	0.94	(0.36 to 2.43)[a]
					Peritoneum	BCG		Ever / Never	1 / 2	16912 / 17852	0.53	(0.05 to 5.82)[a]
					Polycythemia vera	BCG		Ever / Never	0 / 2	16913 / 17852		
					Prostate gland	BCG		Ever / Never	15 / 16	16898 / 17838	0.99	(0.49 to 2.00)[a]
					Rectum	BCG		Ever / Never	10 / 10	16903 / 17844	1.06	(0.44 to 2.54)[a]
					Salivary gland	BCG		Ever / Never	4 / 7	16909 / 17847	0.60	(0.18 to 2.06)[a]
					Skin melanoma	BCG		Ever / Never	7 / 2	16906 / 17852	3.70	(0.77 to 17.79)[a,d]
					Stomach	BCG		Ever / Never	13 / 5	16900 / 17849	2.75	(0.98 to 7.70)[a]
					Thyroid	BCG		Ever / Never	6 / 10	16907 / 17844	0.63	(0.23 to 1.74)[a]
					Tonsil	BCG		Ever / Never	3 / 1	16910 / 17853	3.17	(0.33 to 30.45)[a]
					Uterus	BCG		Ever / Never	18 / 24	16895 / 17830	0.79	(0.43 to 1.46)[a]
Sutherland, 1982[a,c]	15-30		Registry	Exc: rare cancer; Mat: age, sex, race; results from unmatched analyses; population-based	Leukemia death	BCG		Ever / Never	9 / 7	13598 / 12867	1.22	(0.45 to 3.27)[a,d]
					Leukemia death	BCG (Vole-bacillus)		Ever / Never	3 / 7	5817 / 12867	0.95	(0.25 to 3.67)[a]
Auvinen, 2000[c]	0-14	Trial	Registry	Adj: other vaccinations; trial-based	Leukemia	Hib		Early / Late	33 / 44		0.72	(0.46 to 1.13)
					Leukemia	Hib	Diagnosis age 0.25-2	Early / Late	4 / 10		0.47	(0.09 to 2.59)
					ALL	Hib		Early / Late	29 / 38		0.73	(0.45 to 1.18)[d]
					ALL	Hib	Diagnosis age 0.25-2	Early / Late	5 / 1		0.19	(0.00 to 1.72)

Table continues

Supplementary Table 2. Continued

Reference	Age Range	Exposure	Outcome	Comments	Cancer Site	Vaccine	Subsample	Model (Estimate / Ref)	No. of cases (Estimate / Ref)	No. of Controls or Expected (Estimate / Ref)	OR	95%CI
Villumsen, 2009[c]	5-35	Vaccina-tion card	Registry	Adj: day care, family social class; register-based; sub-cohort; update of Danish data in Waaler 1970	Hodgkin lymphoma	BCG	Female	Ever / Never	7 / 4	18092 / 8002	0.45	(0.11 to 1.84)
					Hodgkin lymphoma	BCG	Male	Ever / Never	7 / 6	18081 / 8186	0.37	(0.11 to 1.27)
					Hodgkin lymphoma	BCG		Ever / Never	14 / 10	36172 / 16188	0.41	(0.17 to 1.02)[d]
					Hodgkin lymphoma	Smallpox	Female	Ever / Never	7 / 4	16505 / 9589	1.28	(0.26 to 6.23)
					Hodgkin lymphoma	Smallpox	Male	Ever / Never	5 / 8	15255 / 11012	0.55	(0.14 to 2.09)
					Hodgkin lymphoma	Smallpox		Ever / Never	12 / 12	31759 / 20601	0.86	(0.32 to 2.27)
					Leukemia	BCG	Controlgroup subcohort	Ever / Never	12 / 8	36178 / 16188	0.81	(0.31 to 2.16)[d]
					Leukemia	BCG	Female	Ever / Never	7 / 3	18092 / 8002	0.87	(0.19 to 4.00)
					Leukemia	BCG	Male	Ever / Never	5 / 5	18087 / 8186	0.75	(0.25 to 2.25)
					Leukemia	Smallpox	Controlgroup subcohort	Ever / Never	12 / 8	31765 / 20601	1.32	(0.49 to 3.53)
					Leukemia	Smallpox	Female	Ever / Never	6 / 4	16505 / 9589	1.27	(0.34 to 4.80)
					Leukemia	Smallpox	Male	Ever / Never	6 / 4	15261 / 11012	1.54	(0.35 to 6.84)
					Lymphoma	BCG	Controlgroup subcohort	Ever / Never	33 / 18	36169 / 16188	0.49	(0.26 to 0.93)[d]
					Lymphoma	BCG	Female	Ever / Never	13 / 7	18088 / 8002	0.45	(0.17 to 1.21)
					Lymphoma	BCG	Male	Ever / Never	20 / 11	18081 / 8186	0.52	(0.23 to 1.20)
					Lymphoma	Smallpox	Controlgroup subcohort	Ever / Never	34 / 17	31756 / 20601	1.32	(0.56 to 3.08)
					Lymphoma	Smallpox	Female	Ever / Never	14 / 6	16501 / 9589	1.48	(0.36 to 6.50)
					Lymphoma	Smallpox	Male	Ever / Never	20 / 11	15225 / 11012	1.09	(0.38 to 3.13)
					Non-Hodgkin lymphoma	BCG	Female	Ever / Never	6 / 3	18088 / 8002	0.44	(0.12 to 1.67)
					Non-Hodgkin lymphoma	BCG	Male	Ever / Never	13 / 5	18088 / 8186	0.66	(0.21 to 2.08)
					Non-Hodgkin lymphoma	BCG		Ever / Never	19 / 8	36176 / 16188	0.58	(0.24 to 1.40)
					Non-Hodgkin lymphoma	Smallpox	Female	Ever / Never	7 / 2	16501 / 9589	1.84	(0.11 to 30.4)
					Non-Hodgkin lymphoma	Smallpox	Male	Ever / Never	15 / 3	15262 / 11012	2.75	(0.35 to 21.8)
					Non-Hodgkin lymphoma	Smallpox		Ever / Never	22 / 5	31763 / 20601	2.64	(0.48 to 14.4)
Qu, 2014	0-30	Trial	Registry	Exc: rare cancer; Adj: cluster; trial-based; mortality rates combined with incidence rates	Hepatocellular carcinoma	Hepatitis	Diagnosis age 0-9	Ever / Never	1 / 1	34470 / 30542	0.89	(0.06 to 14.17)[a]
					Hepatocellular carcinoma	Hepatitis	Diagnosis age 10-19	Ever / Never	1 / 3	34470 / 30540	0.30	(0.03 to 2.84)[a]
					Primary liver cancer	Hepatitis		Ever / Never	2 / 12	34469 / 30531	0.16	(0.03 to 0.77)
Soegaard, 2017[c]	0-14	Registry	Registry	Adj: sex, race, birth weight, year, order & mode, other vaccination, gestational age; down syndrome excluded; latency considered; register-based; hazard ratio	ALL	Change in HR per dose	Crude, born 1990-2008	Ever / Never	490	1224914	1.00	(0.96 to 1.05)
					ALL	Change in HR per dose	Adjusted, born 1990-2008	Ever / Never	490	1224914	1.01	(0.93 to 1.10)
					ALL	DTPolio	Crude, born 1990-2008	Ever / Never	486 / 4	1224914	1.07	(0.4 to 2.86)
					ALL	DTPolio	Adjusted, born 1990-2008	Ever / Never	486 / 4	1224914	1.14	(0.42 to 3.13)[d]
					ALL	Hib	Crude, born 1990-2008	Ever / Never	435 / 55	1224914	1.19	(0.9 to 1.57)
					ALL	Hib	Adjusted, born 1990-2008	Ever / Never	435 / 55	1224914	1.04	(0.68 to 1.61)[d]
					ALL	Hib	Adjusted, born 1981-2008	Ever / Never	337 / 0	1225067	0.97	(0.68 to 1.37)
					ALL	MMR	Crude, born 1990-2008	Ever / Never	418 / 72	1224914	1.09	(0.84 to 1.41)
					ALL	MMR	Adjusted, born 1990-2008	Ever / Never	418 / 72	1224914	1.01	(0.76 to 1.34)[d]
					ALL	No Hib	Born < 1993 / > 1993	Ever / Never	1 / -	1225403	0.90	(0.38 to 2.13)
					ALL	Pertussis	Crude, born 1990-2008	Ever / Never	486 / 4	1224914	0.90	(0.75 to 1.09)

Table continues

Supplementary Table 2. Continued

Reference	Age Range	Exposure	Outcome	Comments	Cancer Site	Vaccine	Subsample	Model (Estimate / Ref)	No. of cases (Estimate / Ref)	No. of Controls or Expected (Estimate / Ref)	OR	95%CI
					ALL	Pertussis	Adjusted, born 1990-2008	Ever / Never	486 / 4	1224914	1.10	(0.51 to 2.39)
					ALL	Routine	Adjusted	Ever / No or uncomplete		1225401	0.91	(0.28 to 2.93)[d]
					ALL (prenatal initiated)	Change in HR per dose	Crude, born 1990-2008	Ever / Never	227	1225177	1.00	(0.94 to 1.07)
					ALL (prenatal initiated)	Change in HR per dose	Adjusted, born 1990-2008	Ever / Never	227	1225177	1.09	(0.95 to 1.24)
					ALL (prenatal initiated)	DTPolio	Crude, born 1990-2008	Ever / Never	226 / 1	1225177	2.04	(0.29 to 14.5)
					ALL (prenatal initiated)	DTPolio	Adjusted, born 1990-2008	Ever / Never	226 / 1	1225177	2.47	(0.33 to 18.3)
					ALL (prenatal initiated)	Hib	Crude, born 1990-2008	Ever / Never	217 / 10	1225177	0.95	(0.5 to 1.79)
					ALL (prenatal initiated)	Hib	Adjusted, born 1990-2008	Ever / Never	217 / 0	1225177	0.55	(0.24 to 1.24)
					ALL (prenatal initiated)	MMR	Crude, born 1990-2008	Ever / Never	200 / 27	1225177	1.14	(0.75 to 1.73)
					ALL (prenatal initiated)	MMR	Adjusted, born 1990-2008	Ever / Never	200 / 27	1225177	1.1	(0.71 to 1.71)
					ALL (prenatal initiated)	Pertussis	Crude, born 1990-2008	Ever / Never	227 / 1	1225176	0.82	(0.62 to 1.09)
					ALL (prenatal initiated)	Pertussis	Adjusted, born 1990-2008	Ever / Never	227 / 1	1225176	0.62	(0.23 to 1.67)
					ALL (prenatal initiated)	Routine	Adjusted	Ever / Never	1 / 0	1225403	1.66	(0.22 to12.7)
					BCP ALL	Change in HR per dose	Crude, born 1990-2008	Ever / Never	426	1224978	1.00	(0.96 to 1.05)
					BCP ALL	Change in HR per dose	Adjusted, born 1990-2008	Ever / Never	426	1224978	1.00	(0.92 to1.09)
					BCP ALL	DTPolio	Crude, born 1990-2008	Ever / Never	423 / 3	1224978	1.26	(0.4 to 3.92)
					BCP ALL	DTPolio	Adjusted, born 1990-2008	Ever / Never	423 / 3	1224978	1.38	(0.43 to 4.4)
					BCP ALL	Hib	Crude, born 1990-2008	Ever / Never	378 / 48	1224978	1.17	(0.86 to 1.58)
					BCP ALL	Hib	Adjusted, born 1990-2008	Ever / Never	378 / 48	1224978	0.91	(0.56 to 1.46)
					BCP ALL	Hib	Adjusted, born 1981-2008	Ever / Never	289 / -	1225115	0.84	(0.57 to 1.24)
					BCP ALL	MMR	Crude, born 1990-2008	Ever / Never	362 / 64	1224978	1.08	(0.82 to 1.42)
					BCP ALL	MMR	Adjusted, born 1990-2008	Ever / Never	362 / 64	1224978	1.01	(0.75 to 1.36)
					BCP ALL	Pertussis	Crude, born 1990-2008	Ever / Never	423 / 3	1224978	0.89	(0.73 to 1.09)
					BCP ALL	Pertussis	Adjusted, born 1990-2008	Ever / Never	423 / 3	1224978	1.08	(0.46 to 2.52)
					BCP ALL	Routine	Adjusted	Ever / Never	2 / 2	1225400	1.25	(0.30 to 5.20)
					t-cell ALL	Change in HR per dose	Crude, born 1990-2008	Ever / Never	58	1225346	1.01	(0.88 to 1.16)
					t-cell ALL	Change in HR per dose	Adjusted, born 1990-2008	Ever / Never	58	1225346	1.07	(0.85 to 1.36)
					t-cell ALL	Hib	Crude, born 1990-2008	Ever / Never	58 / 0	1225346	1.43	(0.61 to 3.35)
					t-cell ALL	Hib	Adjusted, born 1990-2008	Ever / Never	58 / 0	1225346	1.66	(0.57 to 4.8)
					t-cell ALL	Hib	Adjusted, born 1981-2008	Ever / Never	38 / 0	1225366	1.60	(0.65 to 3.92)
					t-cell ALL	MMR	Crude, born 1990-2008	Ever / Never	52 / 6	1225346	1.24	(0.53 to2.91)
					t-cell ALL	MMR	Adjusted, born 1990-2008	Ever / Never	52 / 6	1225346	0.95	(0.4 to 2.27)
					t-cell ALL	Pertussis	Crude, born 1990-2008	Ever / Never	58 / 0	1225346	1.15	(0.66 to2.00)
					t-cell ALL	Pertussis	Adjusted, born 1990-2008	Ever / Never	58 / 0	1225346	1.76	(0.69 to 4.47)

Table continues

Supplementary Table 2B. Estimates of case-control studies included in the systematic review

Reference	Age Range	Exposure	Outcome	Comments	Cancer Site	Vaccine	Subsample	Model (Estimate / Ref)	No. of cases (Estimate / Ref)	No. of Controls or Expected (Estimate / Ref)	OR	95%CI
Innis, 1965	Children	Records	Hospital	Exc: without complete estimates; hospital-based; pilot study; letter to editor; original study of Innis 1968	Leukemia	Any (Diphtheria, Pertussis, Tetanus)		Ever / Never	65		x^2: 4.49	P < 0.05
Stewart, 1965[a]	0-9	Self-report	Hospital	Exc: overlapping population; cancer death; population-based; letter to the editor; original study of Kneale 1986 & Gilman 1989	Cancer death	Diphtheria		Ever / Never	768 / 340	781 / 327	0.95	(0.79 to 1.13)[a]
					Cancer death	Pertussis		Ever / Never	527 / 581	571 / 537	0.85	(0.72 to 1.01)[a]
					Cancer death	Smallpox		Ever / Never	455 / 653	488 / 620	0.89	(0.75 to 1.05)[a]
					Cancer death	Polio		Ever / Never	259 / 849	265 / 843	0.97	(0.80 to 1.18)[a]
					Cancer death	Tetanus		Ever / Never	204 / 904	227 / 881	0.88	(0.71 to 1.08)[a]
					Cancer death	BCG		Ever / Never	32 / 1076	29 / 1079	1.11	(0.66 to 1.84)[a]
					Cancer death	Yellow fever		Ever / Never	2 / 1106	0 / 1108		
					Cancer death	Typhoid		Ever / Never	3 / 1105	1 / 1107	3.01	(0.31 to 28.94)[a]
					Leukemia death	Diphtheria		Ever / Never	753 / 246	758 / 241	0.97	(0.79 to 1.19)[a]
					Leukemia death	Pertussis		Ever / Never	536 / 463	561 / 438	0.90	(0.76 to 1.08)[a]
					Leukemia death	Smallpox		Ever / Never	486 / 513	458 / 541	1.12	(0.94 to 1.33)[a]
					Leukemia death	Polio		Ever / Never	290 / 729	259 / 740	1.14	(0.93 to 1.38)[a]
					Leukemia death	Tetanus		Ever / Never	176 / 823	222 / 777	0.75	(0.60 to 0.93)[a]
					Leukemia death	BCG		Ever / Never	27 / 972	29 / 970	0.93	(0.55 to 1.58)[a]
					Leukemia death	Yellow fever		Ever / Never	4 / 995	0 / 999		
					Leukemia death	Typhoid		Ever / Never	3 / 996	0 / 999		
Innis, 1968[a,c]	Children	Records	Hospital	[Mat: age, sex;] hospital-based without cancer; update of Innis 1965	Cancer	BGG	Vaccination age >1	Ever / Never	13 / 693	5 / 701	2.63	(0.93 to 7.42)[a,e]
					Cancer	Cholera	Vaccination age >1	Ever / Never	2 / 704	1 / 705	2.00	(0.18 to 22.14)[a]
					Cancer	Diphtheria	Vaccination age <1	Ever / Never	49 / 61	52 / 58	0.90	(0.53 to 1.52)[a]
					Cancer	Diphtheria	Vaccination age >1	Ever / Never	646 / 60	640 / 66	1.11	(0.77 to 1.60)[a]
					Cancer	Pertussis	Vaccination age <1	Ever / Never	48 / 62	53 / 57	0.83	(0.49 to 1.42)[a]
					Cancer	Pertussis	Vaccination age >1	Ever / Never	624 / 82	626 / 80	0.97	(0.70 to 1.35)[a]
					Cancer	Polio	Vaccination age <1	Ever / Never	29 / 81	28 / 82	1.05	(0.57 to 1.92)[a,d]
					Cancer	Polio	Vaccination age >1	Ever / Never	618 / 88	569 / 137	1.69	(1.26 to 2.26)[a,d]
					Cancer	Smallpox	Vaccination age >1	Ever / Never	29 / 677	20 / 686	1.47	(0.82 to 2.62)[a]
					Cancer	Tetanus	Vaccination age <1	Ever / Never	49 / 61	51 / 59	0.93	(0.55 to 1.58)[a]
					Cancer	Tetanus	Vaccination age >1	Ever / Never	616 / 90	609 / 97	1.09	(0.80 to 1.48)[a]
					Cancer	Typhoid	Vaccination age >1	Ever / Never	0 / 706	2 / 704		
Mathé, 1974[a,c]	<20	Vaccina-tion card	Hospital	[Mat: age]; population-based without cancer; socioeconomical status not considered	Leukemia	BCG		Ever / Never	76 / 130	67 / 130	1.13	(0.75 to 1.71)[a,d]

Table continues

Supplementary Table 2. Continued

Reference	Age Range	Exposure	Outcome	Comments	Cancer Site	Vaccine	Subsample	Model (Estimate / Ref)	No. of cases (Estimate / Ref)	No. of Controls or Expected (Estimate / Ref)	OR	95%CI
Salonen, 1975[c]	<15	Records	Registry	Mat: age, area, birth season; population-based without cancer; original study of Salonen 1976	Bone Tumor	BCG		Ever / Never			1.3	(0.17 to 13)[d]
					Brain Tumor	BCG		Ever / Never	670 / 74	666 / 83	1.1	(0.41 to 2.9)[d]
					Brain Tumor	Polio		Ever / Never			0.29	(0.02 to 1.9)[d]
					Cancer	BCG		Ever / Never	674 / 70	677 / 72	0.96	(0.59 to 1.6)[d]
					Cancer	Polio		Ever / Never			1.0	(0.43 to 2.3)[d]
					Eye Tumor	BCG		Ever / Never			0.33	(0.01 to 6.1)
					Kidney Tumor	BCG		Ever / Never			1.1	(0.3 to 4.6)[d]
					Leukemia	BCG		Ever / Never	694 / 50	707 / 42	0.8	(0.29 to 2.1)[d]
					Leukemia	Polio		Ever / Never			1.8	(0.36 to 11.0)[d]
					Other Tumor	BCG		Ever / Never	654 / 90	651 / 98	1.0	(0.48 to 2.1)
					Other Tumor	Polio		Ever / Never			1.3	(0.33 to 5.9)
Salonen, 1976[a,c]	<15	Records	Registry	[Mat: age, area, birth season;] population-based without cancer; update of Salonen 1975	Bone Tumor	BCG	Vaccination newborns	Ever / Never	42 / 14	39 / 17	1.31	(0.57 to 3.00)[a]
					Brain Tumor	Any	Vaccination newborns	Ever / Never	221 / 24	218 / 27	1.14	(0.64 to 2.04)[a]
					Brain Tumor	Any	Vaccination prenatal	Ever / Never	6 / 239	11 / 234	0.53	(0.19 to 1.47)[a]
					Cancer	Any	Vaccination newborns	Ever / Never	881 / 91	879 / 93	1.02	(0.76 to 1.39)[a,d]
					Cancer	Any	Vaccination prenatal	Ever / Never	32 / 940	32 / 940	1.00	(0.61 to 1.65)[a]
					Eye Tumor	Any	Vaccination prenatal	Ever / Never	3 / 34	7 / 30	0.38	(0.09 to 1.59)[a]
					Eye Tumor	BCG	Vaccination newborns	Ever / Never	34 / 3	36 / 1	0.31	(0.03 to 3.18)[a]
					Kidney Tumor	Any	Vaccination prenatal	Ever / Never	6 / 90	2 / 94	3.13	(0.62 to 15.93)[a]
					Kidney Tumor	BCG	Vaccination newborns	Ever / Never	86 / 10	85 / 11	1.11	(0.45 to 2.76)[a]
					Leukemia	Any	Vaccination newborns	Ever / Never	348 / 25	352 / 21	0.83	(0.46 to 1.51)[a,d]
					Leukemia	Any	Vaccination prenatal	Ever / Never	11 / 362	8 / 365	1.39	(0.55 to 3.49)[a]
					Other Tumor	Any	Vaccination newborns	Ever / Never	311 / 43	308 / 46	1.08	(0.69 to 1.68)[a]
					Other Tumor	Any	Vaccination prenatal	Ever / Never	15 / 339	13 / 341	1.16	(0.54 to 2.48)[a]
Andersen, 1978[a,c]	School Children	Records	Registry	[Mat: age, sex, socioeconomical status;] 1:3; population-based without cancer; Fisher´s exact test	Hodgkin lymphoma	BCG	Vaccination 1 time	Ever / Never	40 / 15	111 / 41	0.98	(0.49 to 1.97)[a,d]
					Hodgkin lymphoma	BCG	Vaccination 2+ times	Ever / Never	2 / 15	13 / 41	0.42	(0.08 to 2.09)[a]
Farwell, 1979[a,c]	≤19	Self-report	Registry	[Mat: age, sex, area of residence;] original study of Farwell 1984	CNS tumor	Polio	Vaccination prenatal	Ever / Never	19 / 33	8 / 30	2.16	(0.82 to 5.65)[a,d]
					Glioma	Polio	Vaccination prenatal	Ever / Never	8 / 15	8 / 30	2.00	(0.63 to 6.38)[a]
					Medulloblastoma	Polio	Vaccination prenatal	Ever / Never	10 / 15	8 / 30	2.50	(0.82 to 7.64)[a]
Neumann, 1980[a,c]	0-14	Self-report	Death certificates	Cancer death; [Mat: age, sex;] population-based; article in German	Cancer death	BCG		Ever / Never	31 / 33	37 / 27	0.69	(0.34 to 1.38)[a,d]
					Cancer death	BCG	Death age 0-5	Ever / Never	16 / 5	15 / 6	1.28	(0.32 to 5.09)[a]
					Cancer death	BCG	Death age 5-10.	Ever / Never	14 / 15	20 / 9	0.42	(0.14 to 1.23)[a]
					Cancer death	BCG	Death age 10-15	Ever / Never	1 / 13	2 / 12	0.46	(0.04 to 5.77)[a]
					Cancer death	Diphtheria		Ever / Never	48 / 16	59 / 5	0.25	(0.09 to 0.74)[a]
					Cancer death	Diphtheria	Death age 0-5	Ever / Never	13 / 8	17 / 4	0.38	(0.09 to 1.55)[a]
					Cancer death	Diphtheria	Death age 5-10.	Ever / Never	25 / 4	29 / 0		
					Cancer death	Diphtheria	Death age 10-15	Ever / Never	10 / 4	13 / 1	0.19	(0.02 to 2.00)[a]

Table continues

Supplementary Table 2. Continued

Reference	Age Range	Exposure	Outcome	Comments	Cancer Site	Vaccine	Subsample	Model (Estimate / Ref)	No. of cases (Estimate / Ref)	No. of Controls or Expected (Estimate / Ref)	OR	95%CI
					Cancer death	Pocks		Ever / Never	49 / 23	45 / 27	1.28	(0.64 to 2.54)[a]
					Cancer death	Pocks	Death age 0-5	Ever / Never	5 / 17	9 / 13	0.42	(0.11 to 1.57)[a]
					Cancer death	Pocks	Death age 5-10.	Ever / Never	29 / 4	19 / 14	5.34	(1.53 to 18.70)[a]
					Cancer death	Pocks	Death age 10-15	Ever / Never	15 / 2	17 / 0		
					Cancer death	Polio		Ever / Never	55 / 9	57 / 7	0.75	(0.26 to 2.16)[a]
					Cancer death	Polio	Death age 0-5	Ever / Never	15 / 6	16 / 5	0.78	(0.20 to 3.11)[a]
					Cancer death	Polio	Death age 5-10.	Ever / Never	27 / 2	29 / 0		
					Cancer death	Polio	Death age 10-15	Ever / Never	13 / 1	12 / 2	2.17	(0.17 to 27.08)[a]
					Cancer death	Tetanus		Ever / Never	54 / 10	59 / 5	0.46	(0.15 to 1.42)[a]
					Cancer death	Tetanus	Death age 0-5	Ever / Never	13 / 8	17 / 4	0.38	(0.09 to 1.55)[a]
					Cancer death	Tetanus	Death age 5-10.	Ever / Never	28 / 1	29 / 0		
					Cancer death	Tetanus	Death age 10-15	Ever / Never	13 / 1	13 / 1	1.00	(0.06 to 17.75)[a]
					Leukemia death	BCG		Ever / Never	15 / 17	20 / 12	0.53	(0.20 to 1.43)[a,d]
					Leukemia death	BCG	Death age 0-5	Ever / Never	8 / 3	8 / 3	1.00	(0.15 to 6.53)[a]
					Leukemia death	BCG	Death age 5-10.	Ever / Never	6 / 9	11 / 4	0.24	(0.05 to 1.13)[a]
					Leukemia death	BCG	Death age 10-15	Ever / Never	1 / 5	1 / 5	1.00	(0.05 to 20.83)[a]
					Cancer death, other than Leukemia	BCG		Ever / Never	16 / 16	17 / 15	0.88	(0.33 to 2.35)[a]
					Cancer death, other than Leukemia	BCG	Death age 0-5	Ever / Never	8 / 2	7 / 3	1.71	(0.22 to 13.41)[a]
					Cancer death, other than Leukemia	BCG	Death age 5-10.	Ever / Never	8 / 6	9 / 5	0.74	(0.16 to 3.39)[a]
					Cancer death, other than Leukemia	BCG	Death age 10-15	Ever / Never	0 / 8	1 / 7		
Gruffermann, 1982	0-14	Self-report	Hospital	Exc: rare cancer; Mat: age, sex, race; results from unmatched analyses; population-based	Rhabdomyosarcoma	Any		Ever / Never	32 / -	96 / -	0.7	(0.1 to 7.6)
					Rhabdomyosarcoma	DTP		Ever / Never	32 / -	96 / -	0.7	(0.1 to 7.6)
					Rhabdomyosarcoma	MMR		Ever / Never	29 / -	93 / -	0.3	(0.1 to 1.5)
					Rhabdomyosarcoma	Smallpox		Ever / Never	21 / -	88 / -	0.2	(0.1 to 0.6)
Farwell, 1984	≤19	Self-report	Registry	Exc: without complete estimates; Mat: age, sex, area of residence; information for prenatal vaccination for ~60% of cases and controls; update of Farwell 1979	Medulloblastoma	Polio		Ever / Never	57% / 43 %	27% / 73%		
Van Steensel-Moll, 1985[c]	<15	Self-report	Registry	Mat: age, sex, area; Adj: age, sex; population-based	Leukemia	Any		Ever / Never	15 / 610	14 / 601	0.8	(0.4 to 1.6)[d]

Table continues

Supplementary Table 2. Continued

Reference	Age Range	Exposure	Outcome	Comments	Cancer Site	Vaccine	Subsample	Model (Estimate / Ref)	No. of cases (Estimate / Ref)	No. of Controls or Expected (Estimate / Ref)	OR	95%CI
Kneale, 1986[c]	0-15	Self-report	Hospital	Cancer death; Mat: sex, area, birth date (birth year, season); % risk; population-based child alive; update of Stewart 1965 & original study of Gilman 1989	Cancer death	Any	Diagnosis age 0-1, vaccination age 0-1	Ever / Never			12.6%	SE: 2.4; P > 0.01
					Cancer death	Any	Diagnosis age 2-4, vaccination age 0-1	Ever / Never			7.6%	SE: 2; P > 0.01
					Cancer death	Any	Diagnosis age 2-4, vaccination age 2-4	Ever / Never			14.4%	SE: 5.8; P > 0.01
					Cancer death	Any	Diagnosis age 10-15, vaccination age 0-1	Ever / Never			7.4%	SE: 2.6; P > 0.05
					Cancer death	Any	Diagnosis age 10-15, vaccination age 0-1	Ever / Never			7.4%	SE: 2.6; P > 0.05
					Cancer death	Any	Diagnosis age 10-15, vaccination age 10-15	Ever / Never			24%	SE: 7.2; P > 0.01
					Cancer death	Any	Diagnosis age 10-15, vaccination all ages	Ever / Never			8.9%	SE: 1.1; P > 0.01
					Cancer death	BGC		Ever / Never	792 / 11489	883 / 11398	0.82	(0.72 to 0.93)[b,d]
					Cancer death	DT		Ever / Never	7797 / 4484	8110 / 4171	0.80	(0.75 to 0.86)[b]
					Cancer death	Measles		Ever / Never	622 / 11659	676 / 11605	0.83	(0.70 to 0.98)[b]
					Cancer death	Pertussis		Ever / Never	6623 / 5658	7022 / 5259	0.78	(0.73 to 0.84)[b]
					Cancer death	Polio		Ever / Never	5295 / 6986	5649 / 6632	0.81	(0.75 to 0.86)[b]
					Cancer death	Rubella		Ever / Never	22 / 12259	34 / 12247	0.57	(0.31 to 1.06)[b]
					Cancer death	Smallpox		Ever / Never	4419 / 7862	4615 / 7666	0.90	(0.85 to 0.96)[b]
					Cerebral tumor death	Any		Ever / Never			9.7%	SE: 2.9; P > 0.01
					Leukemia death	Any		Ever / Never			7%	SE: 1.7; P > 0.01
					Lymphoma death	Any		Ever / Never			9.1%	SE: 3.7; P > 0.05
					Neuroblastoma death	Any		Ever / Never			12.5%	SE: 4.1; P > 0.01
					Osteosarcoma death	Any		Ever / Never			16.9%	SE: 5.7; P > 0.05
					Other solid tumor death	Any		Ever / Never			6.9%	SE: 1.5; P > 0.01
					Wilms tumor death	Any		Ever / Never			15.6%	SE: 4.5; P > 0.01
McKinney, 1987[c]	1-15	Self-report	Registry	Mat: age, sex; hospital-based without cancer; original study of Hartley 1988	Leukemia	Any (T, D, P, Polio, Mea, triple, Sma)		Ever / Never	171 / -	342 / -	0.2	(0.1 to 0.9)[d]
					Myeloid Leukemia	Any (T, D, P, Polio, Mea, triple, Sma)		Ever / Never	23 / -	46 / -	0.1	(0.0 to1.0)
					Leukemia and Lymphoma	Any (T, D, P, Polio, Mea, triple, Sma)		Ever / Never	234 / -	468 / -	0.3	(0.1 to 1.1)
					Lymphoma	Any (T, D, P, Polio, Mea, triple, Sma)		Ever / Never	63 / -	126 / -	2	(0.1 to 11.5)
Hartley, 1988	2-14	Self-report	Registry	Exc: rare vaccination; Mat: age, sex; hospital-based without cancer; never versus 1+; update of McKinney 1978	Cancer	Any (T, D, P, Polio, R, Mea, Sma)		Ever / Never	439 / -		3.58	(1.57 to 8.15)

Table continues

Supplementary Table 2. Continued

Reference	Age Range	Exposure	Outcome	Comments	Cancer Site	Vaccine	Subsample	Model (Estimate / Ref)	No. of cases (Estimate / Ref)	No. of Controls or Expected (Estimate / Ref)	OR	95%CI
Gilman, 1989	Children	Self-report	Hospital	Exc: rare vaccinantion; cancer death; Mat: sex, area, birth date (birth year, season); Self-report supplied by general practitioner in 55% of cases and 59% of controls; population-based child alive; update of Stewart 1965 & Kneale 1986	All cancers	Any (Gamma globulin, Influenza, Polio, Smallpox, Tetanus, other and unspecified)	Adjustment set 1 (Paper Tab11b)	Ever / Never	139 / 7920	97 / 7962	1.36	$P < 0.05$; β=0.305
					All cancers	Any (Gamma globulin, Influenza, Polio, Smallpox, Tetanus, other and unspecified)	Adjustment set 1 (Paper Tab11b)	Ever / Never	139 / 7920	97 / 7962	1.44	(1.11 to 1.86)[a]
					Reticulo-endothelial system neoplasms	Any (Gamma globulin, Influenza, Polio, Smallpox, Tetanus, other and unspecified)	Adjustment set 1 (Paper Tab11b)	Ever / Never	139 / 7920	97 / 7962	1.79	$P < 0.05$; β=0.584
					Reticulo-endothelial system neoplasms	Any (Gamma globulin, Influenza, Polio, Smallpox, Tetanus, other and unspecified)	Adjustment set 1 (Paper Tab11b)	Ever / Never	139 / 7920	97 / 7962	1.44	(1.11 to 1.86)[a]
					Solid cancers	Any (Gamma globulin, Influenza, Polio, Smallpox, Tetanus, other and unspecified)	Adjustment set 1 (Paper Tab11b)	Ever / Never	139 / 7920	97 / 7962		β=0.047
					Solid cancers	Any (Gamma globulin, Influenza, Polio, Smallpox, Tetanus, other and unspecified)	Adjustment set 1 (Paper Tab11b)	Ever / Never	139 / 7920	97 / 7962	1.44	(1.11 to 1.86)[a]
					All cancers	Any (Gamma globulin, Influenza, Polio, Smallpox, Tetanus, other and unspecified)	Adjustment set 2 (Paper Tab12)	Ever / Never	139 / 7920	97 / 7962	1.37	$P < 0.05$; β=0.317
					All cancers	Any (Gamma globulin, Influenza, Polio, Smallpox, Tetanus, other and unspecified)	Adjustment set 2 (Paper Tab12)	Ever / Never	139 / 7920	97 / 7962	1.44	(1.11 to 1.86)[a]
					Reticulo-endothelial system neoplasms	Any (Gamma globulin, Influenza, Polio, Smallpox, Tetanus, other and unspecified)	Adjustment set 2 (Paper Tab12)	Ever / Never	139 / 7920	97 / 7962	1.78	$P < 0.05$; β=0.577
					Reticulo-endothelial system neoplasms	Any (Gamma globulin, Influenza, Polio, Smallpox, Tetanus, other and unspecified)	Adjustment set 2 (Paper Tab12)	Ever / Never	139 / 7920	97 / 7962	1.44	(1.11 to 1.86)[a]
					Solid cancers	Any (Gamma globulin, Influenza, Polio, Smallpox, Tetanus, other and unspecified)	Adjustment set 2 (Paper Tab12)	Ever / Never	139 / 7920	97 / 7962		β=0.074

Table continues

Supplementary Table 2. Continued

Reference	Age Range	Exposure	Outcome	Comments	Cancer Site	Vaccine	Subsample	Model (Estimate / Ref)	No. of cases (Estimate / Ref)	No. of Controls or Expected (Estimate / Ref)	OR	95%CI
					Solid cancers	Any (Gamma globulin, Influenza, Polio, Smallpox, Tetanus, other and unspecified)	Adjustment set 2 (Paper Tab12)	Ever / Never	139 / 7920	97 / 7962	1.44	(1.11 to 1.86)[a]
					All cancers	Any (Gamma globulin, Influenza, Polio, Smallpox, Tetanus, other and unspecified)	Adjustment set 3 (Paper Tab13)	Ever / Never	139 / 7920	97 / 7962	1.37	P < 0.05; β=0.316
					All cancers	Any (Gamma globulin, Influenza, Polio, Smallpox, Tetanus, other and unspecified)	Adjustment set 3 (Paper Tab13)	Ever / Never	139 / 7920	97 / 7962	1.44	(1.11 to 1.86)[a]
					Reticulo-endothelial system neoplasms	Any (Gamma globulin, Influenza, Polio, Smallpox, Tetanus, other and unspecified)	Adjustment set 3 (Paper Tab13)	Ever / Never	139 / 7920	97 / 7962	1.75	(P < 0.05; β=0.562)
					Reticulo-endothelial system neoplasms	Any (Gamma globulin, Influenza, Polio, Smallpox, Tetanus, other and unspecified)	Adjustment set 3 (Paper Tab13)	Ever / Never	139 / 7920	97 / 7962	1.44	(1.11 to 1.86)[a]
					Solid cancers	Any (Gamma globulin, Influenza, Polio, Smallpox, Tetanus, other and unspecified)	Adjustment set 3 (Paper Tab13)	Ever / Never	139 / 7920	97 / 7962		β=0.089
					Solid cancers	Any (Gamma globulin, Influenza, Polio, Smallpox, Tetanus, other and unspecified)	Adjustment set 3 (Paper Tab13)	Ever / Never	139 / 7920	97 / 7962	1.44	(1.11 to 1.86)[a]
					All cancers	Any (Gamma globulin, Influenza, Polio, Smallpox, Tetanus, other and unspecified)		Ever / Never	138 / 7920	97 / 7962	1.42	(P <0.01)
					All cancers	Gamma globulin		Ever / Never	57 / 7920	39 / 7962	1.47	(0,98 to 2.21)[a]
					All cancers	Influenza		Ever / Never	5 / 7920	1 / 7962	5.03	(0.59 to 43.01)
					All cancers	Polio		Ever / Never	47 / 7920	46 / 7962	1.03	(0.68 to 1.54)
					All cancers	Smallpox		Ever / Never	9 / 7920	1 / 7962	9.05	(1.15 to 71.40)
					All cancers	Tetanus		Ever / Never	7 / 7920	2 / 7962	3.52	(0.73 to 16.93)
Nishi, 1989[c]	0-15	Self-report	Hospital	Mat: age, sex, area; hospital-based	Leukemia (non-t cell ALL)	BCG		Ever / Never	63 / -	126 / -	0.26	(0.09 to 0.78)
					Leukemia (non-t cell ALL)	BCG	Diagnosis age 0-2	Ever / Never	16 / -	16 / -	0.33	(0.03 to 4.16)
					Leukemia (non-t cell ALL)	BCG	Diagnosis age 3-6	Ever / Never	25 / -	50 / -	0.07	(0.01 to 0.58)
					Leukemia (non-t cell ALL)	BCG	Diagnosis age 7 -14	Ever / Never	22 / -	44 / -	1.5	(0.16 to 14.4)
					Leukemia (non-t cell ALL)	Measles		Ever / Never	63 / -	126 / -	0.24	(0.1 to 0.6)[d]

Table continues

Supplementary Table 2. Continued

Reference	Age Range	Exposure	Outcome	Comments	Cancer Site	Vaccine	Subsample	Model (Estimate / Ref)	No. of cases (Estimate / Ref)	No. of Controls or Expected (Estimate / Ref)	OR	95%CI
					Leukemia (non-t cell ALL)	Measles	Diagnosis age 0-2	Ever / Never	16 / -	32 / -	0.14	(0.03 to 0.69)
					Leukemia (non-t cell ALL)	Measles	Diagnosis age 3-6	Ever / Never	25 / -	50 / -	0.17	(0.19 to 2.74)
					Leukemia (non-t cell ALL)	Measles	Diagnosis age 7 -14	Ever / Never	22 / -	44 / -	0	(p: <0,05)
					Leukemia (non-t cell ALL)	Measles	RR conditional logistic regression	Ever / Never	- / -	- / -	0.1	(0.7; -)
Buckley, 1994	0+	Self-report	Hospital	Exc: without complete estimates; Mat: age, race, area, family income; Children Cancer Group (CCG) study; population-based with & without cancer	ALL	MMR	Cancer controls	Ever / Never	990 / -	1636 / -	1.2	
					ALL	MMR	Population controls	Ever / Never	404 / -	440 / -	1.3	
					ALL	Smallpox	Cancer controls	Ever / Never	990	1636	1.0	
					ALL	Smallpox	Population controls	Ever / Never	404	440	0.7	P < 0.01
					cALL	MMR	Cancer controls	Ever / Never	286 / -	572 / -	1.7	P < 0.01
					cALL	MMR	Population controls	Ever / Never	177 / -	177 / -	1.5	
					cALL	Smallpox	Cancer controls	Ever / Never	286	572	1.2	
					cALL	Smallpox	Population controls	Ever / Never	177	177	0.3	
					null ALL	MMR	Cancer controls	Ever / Never	65 / -	193 / -	0.7	
					null ALL	MMR	Population controls	Ever / Never	61 / -	61 / -	0.7	
					null ALL	Smallpox	Cancer controls	Ever / Never	65	193	1.1	
					null ALL	Smallpox	Population controls	Ever / Never	61	61	0.5	
					pre-b ALL	MMR	Cancer controls	Ever / Never	38 / -	114 / -	2.2	
					pre-b ALL	MMR	Population controls	Ever / Never	36 / -	72 / -	3.7	
					pre-b ALL	Smallpox	Cancer controls	Ever / Never	38	114	0.6	
					pre-b ALL	Smallpox	Population controls	Ever / Never	36	72	1.2	
					t-cell ALL	MMR	Cancer controls	Ever / Never	158 / -	314 / -	0.7	
					t-cell ALL	MMR	Population controls	Ever / Never	130 / -	130 / -	1.1	
					t-cell ALL	Smallpox	Cancer controls	Ever / Never	158	314	0.8	
					t-cell ALL	Smallpox	Population controls	Ever / Never	130	130	0.5	P < 0.01
					Unknown ALL	MMR	Cancer controls	Ever / Never	443 / -	443 / -	1.8	P < 0.001
					Unknown ALL	Smallpox	Cancer controls	Ever / Never	443	443	1.0	
Lewis, 1994	30-79	Self-report	Registry	Adj: age, sex, education, study site; population-based	Multiple Myeloma	childhood vaccines	Whites	Ever / Never	339 / 8	1071 / 15	0.7	(0.3 to 1.8)
					Multiple Myeloma	childhood vaccines	Blacks	Ever / Never	186 / 10	880 / 34	0.8	(0.4 to 1.6)
					Multiple Myeloma	childhood vaccines	Total	Ever / Never	525 / 18	1951 / 49	0.7	(0.4 to 1.3)
					Multiple Myeloma	childhood vaccines	Whites	1-2 / Never	56 / 8	132 / 15	0.7	(0.3 to 1.8)
					Multiple Myeloma	childhood vaccines	Blacks	1-2 / Never	44 / 10	183 / 34	0.8	(0.4 to 1.9)
					Multiple Myeloma	childhood vaccines	Total	1-2 / Never	100 / 18	315 / 49	0.8	(0.4 to 1.5)
					Multiple Myeloma	childhood vaccines	Whites	3-4 / Never	123 / 8	351 / 15	0.7	(0.3 to 1.8)
					Multiple Myeloma	childhood vaccines	Blacks	3-4 / Never	78 / 10	356 / 34	0.8	(0.4 to 1.6)
					Multiple Myeloma	childhood vaccines	Total	3-4 / Never	201 / 18	707 / 49	0.7	(0.4 to 1.3)
					Multiple Myeloma	childhood vaccines	Whites	5+ / Never	160 / 8	588 / 15	0.7	(0.3 to 1.9)
					Multiple Myeloma	childhood vaccines	Blacks	5+ / Never	64 / 10	341 / 34	0.7	(0.3 to 1.5)
					Multiple Myeloma	childhood vaccines	Total	5+ / Never	224 / 18	929 / 49	0.7	(0.4 to 1.3)

Table continues

Supplementary Table 2. Continued

Reference	Age Range	Exposure	Outcome	Comments	Cancer Site	Vaccine	Subsample	Model (Estimate / Ref)	No. of cases (Estimate / Ref)	No. of Controls or Expected (Estimate / Ref)	OR	95%CI
					IgG myeloma	childhood vaccines	Whites	Ever / Never	129 / -	1071 / -	1.1	(0.2 to 5.0)
					IgG myeloma	childhood vaccines	Blacks	Ever / Never	99 / -	880 / -	1.3	(0.4 to 4.2)
					IgG myeloma	childhood vaccines	Total	Ever / Never	228 / -	1951 / -	1.2	(0.5 to 3.1)
					IgA myeloma	childhood vaccines	Whites	Ever / Never	65 / -	1071 / -	0.6	(0.1 to 2.8)
					IgA myeloma	childhood vaccines	Blacks	Ever / Never	27 / -	880 / -	0.5	(0.1 to 2.4)
					IgA myeloma	childhood vaccines	Total	Ever / Never	92 / -	1951 / -	0.6	(0.2 to 1.7)
Shu, 1995	0-15	Self-report	Hospital	Exc: rare cancer; Adj: age, sex, gestational age, number livebirth (birth order), smoking during pregnancy, maternal education; population-based	Malignant germ-cell tumors	Diphtheria		Ever / Never	103 / -	629 / -	2.4	(0.4 to 14.4)
					Malignant germ-cell tumors	Measles		Ever / Never	85 / -	583 / -	0.7	(0.3 to 1.5)
					Malignant germ-cell tumors	Mumps		Ever / Never	86 / -	563 / -	1.2	(0.6 to 2.5)
					Malignant germ-cell tumors	Pertussis		Ever / Never	98 / -	619 / -	0.8	(0.3 to 2.3)
					Malignant germ-cell tumors	Polio		Ever / Never	98 / -	615 / -	1.1	(0.4 to 3.4)
					Malignant germ-cell tumors	Rubella		Ever / Never	87 / -	579 / -	1	(0.5 to 2.2)
					Malignant germ-cell tumors	Smallpox		Ever / Never	45 / -	397 / -	0.5	(0.3 to 0.9)
					Malignant germ-cell tumors	Tetanus		Ever / Never	102 / -	618 / -	2	(0.5 to 8.6)
Kaatsch, 1996	0-15	Self-report	Registry	Exc: without complete estimates; Mat: age, sex; population-based without & with cancer; original study Kaatsch 1998, Schüz 1999 & von Kries 2000	Leukemia	not specified		Ever / Never	348	433		
Bhatia, 1997	0-15	Self-report	Records	Exc: rare cancer; Adj: age at questionnaire, area, birth year & order, annual household income, paternal education, maternal age; compared to community & cancer controls	Langerhans cell histocytosis (multisystem)	Diphtheria	Community controls	Ever / Never			0.2	(0.0 to 0.8)
					Langerhans cell histocytosis (multisystem)	Diphtheria	Cancer controls	Ever / Never			0.4	(0.2 to 1.0)
					Langerhans cell histocytosis (multisystem)	Pertussis	Community controls	Ever / Never			0.2	(0.1 to 0.6)
					Langerhans cell histocytosis (multisystem)	Pertussis	Cancer controls	Ever / Never			0.4	(0.2 to 0.6)
					Langerhans cell histocytosis (multisystem)	Tetanus	Community controls	Ever / Never			0.2	(0.0 to 0.7)
					Langerhans cell histocytosis (multisystem)	Tetanus	Cancer controls	Ever / Never			0.4	(0.2 to 0.8)
					Langerhans cell histocytosis (multisystem)	Measles	Community controls	Ever / Never			0.2	(0.1 to 0.4)

Table continues

Supplementary Table 2. Continued

Reference	Age Range	Exposure	Outcome	Comments	Cancer Site	Vaccine	Subsample	Model (Estimate / Ref)	No. of cases (Estimate / Ref)	No. of Controls or Expected (Estimate / Ref)	OR	95%CI
					Langerhans cell histocytosis (multisystem)	Measles	Cancer controls	Ever / Never			0.4	(0.2 to 0.7)
					Langerhans cell histocytosis (multisystem)	Rubella	Community controls	Ever / Never			0.1	(0.0 to 0.4)
					Langerhans cell histocytosis (multisystem)	Rubella	Cancer controls	Ever / Never			0.4	(0.2 to 0.7)
					Langerhans cell histocytosis (multisystem)	Mumps	Community controls	Ever / Never			0.2	(0.1 to 0.4)
					Langerhans cell histocytosis (multisystem)	Mumps	Cancer controls	Ever / Never			0.3	(0.2 to 0.5)
					Langerhans cell histocytosis (multisystem)	Polio	Community controls	Ever / Never			0.3	(0.1 to 1.0)
					Langerhans cell histocytosis (multisystem)	Polio	Cancer controls	Ever / Never			0.5	(0.2 to 0.9)
					Langerhans cell histo-cytosis (single-system)	Diphtheria	Community controls	Ever / Never			0.1	(0.0 to 0.4)
					Langerhans cell histo-cytosis (single-system)	Diphtheria	Cancer controls	Ever / Never			0.4	(0.1 to 1.1)
					Langerhans cell histo-cytosis (single-system)	Pertussis	Community controls	Ever / Never			0.1	(0.0 to 0.5)
					Langerhans cell histo-cytosis (single-system)	Pertussis	Cancer controls	Ever / Never			0.4	(0.2 to 0.9)
					Langerhans cell histo-cytosis (single-system)	Tetanus	Community controls	Ever / Never			0.2	(0.1 to 1.0)
					Langerhans cell histo-cytosis (single-system)	Tetanus	Cancer controls	Ever / Never			0.5	(0.2 to 1.2)
					Langerhans cell histo-cytosis (single-system)	Measles	Community controls	Ever / Never			0.3	(0.1 to 1.1)
					Langerhans cell histo-cytosis (single-system)	Measles	Cancer controls	Ever / Never			0.7	(0.4 to 1.6)
					Langerhans cell histo-cytosis (single-system)	Rubella	Community controls	Ever / Never			0.2	(0.1 to 0.7)
					Langerhans cell histo-cytosis (single-system)	Rubella	Cancer controls	Ever / Never			0.7	(0.3 to 1.4)
					Langerhans cell histo-cytosis (single-system)	Mumps	Community controls	Ever / Never			0.3	(0.1 to 0.8)
					Langerhans cell histo-cytosis (single-system)	Mumps	Cancer controls	Ever / Never			0.5	(0.2 to 0.9)
					Langerhans cell histo-cytosis (single-system)	Polio	Community controls	Ever / Never			0.2	(0.1 to 0.8)
					Langerhans cell histo-cytosis (single-system)	Polio	Cancer controls	Ever / Never			0.6	(0.3 to 1.3)

Table continues

Supplementary Table 2. Continued

Reference	Age Range	Exposure	Outcome	Comments	Cancer Site	Vaccine	Subsample	Model (Estimate / Ref)	No. of cases (Estimate / Ref)	No. of Controls or Expected (Estimate / Ref)	OR	95%CI
Petridou, 1997[c]	0-15	Self-report	Hospital	Mat: age, sex, area; hospital-based without cancer	Leukemia	BCG		Ever / Never	22 / 131	40 / 260	1.44	(0.66 to 3.13)[d]
					Leukemia	DPT		Increment by ~3 shots	- / 14	- / 49	0.97	(0.71 to 1.32)[d]
					Leukemia	DPT		1-8 shots / Never	27 / 14	42 / 49	2.25	(1.05 to 4.84)[a]
					Leukemia	DPT		9-11 shots / Never	37 / 14	51 / 49	2.54	(1.22 to 5.27)[a]
					Leukemia	DPT		12-13 shots / Never	36 / 14	97 / 49	1.30	(0.64 to 2.63)[a]
					Leukemia	DPT		≥14 shots / Never	39 / 14	61 / 49	2.24	(1.09 to 4.58)[a]
					Leukemia	MMR&Hep		Increment by ~3 shots	- / 15	- / 51	1.23	(0.91 to 1.66)[d]
					Leukemia	MMR&Hep		1-8 shots / Never	14 / 15	30 / 51	1.59	(0.67 to 3.74)[a]
					Leukemia	MMR&Hep		9-12 shots / Never	35 / 15	65 / 51	1.83	(0.90 to 3.71)[a]
					Leukemia	MMR&Hep		13-15 shots / Never	51 / 15	81 / 51	2.14	(1.09 to 4.20)[a]
					Leukemia	MMR&Hep		≥16 shots / Never	38 / 15	73 / 51	1.77	(0.88 to 3.55)[a]
Kaatsch, 1998[c]	0-15	Self-report	Registry	Adj: socio-economic status, urban-rural status; Mat: age, sex, area; population-based; update Kaatsch 1996 & original study Schüz 1999 & von Kries 2000	Leukemia	Any		0-3 / >6	79 / 263	42 / 334	2.79	(1.79 to 4.35)
					Leukemia	Any		>6 / 0-3	263 / 79	334 / 42	0.36	(0.23 to 0.56)[d,e]
					Leukemia	Any		4-6 / >6	312 / 263	278 / 334	1.51	(1.18 to 1.92)
Dockerty, 1999[c]	0-15	Records (parent held)	Registry	Adj: age, sex; Mat: age, sex; latency considered; population-based	Leukemia	Any	Adjusted for age, sex	Ever / Never	81 / 37	209 / 78	0.62	(0.37 to 1.03)
					Leukemia	Any	Adjusted for age, sex, other	Ever / Never	81 / 37	209 / 78	0.71	(0.36 to 1.38)[d]
					Leukemia	BCG	Adjusted for age, sex	Ever / Never	8 / 112	11 / 284	1.55	(0.6 to 4.05)
					Leukemia	BCG	Adjusted for age, sex, other	Ever / Never	8 / 112	11 / 284	1.05	(0.33 to 3.32)[d]
					Leukemia	BCG (Mantoux)	Adjusted for age, sex	Ever / Never	2 / 118	2 / 295	2.08	(0.27 to 15.95)
					Leukemia	BCG (Mantoux)	Adjusted for age, sex, other	Ever / Never	2 / 118	2 / 295	1.74	(0.19 to 15.86)
					Leukemia	Any	Adjusted for age, sex	1 or 2 / Never	3 / 37	19 / 78	0.26	(0.07 to 0.95)
					Leukemia	Any	Adjusted for age, sex	3 or 4 / Never	34 / 37	111 / 78	0.55	(0.3 to 0.98)
					Leukemia	Any	Adjusted for age, sex	5 or more / Never	44 / 37	79 / 78	0.8	(0.44 to 1.46)
					Leukemia	Any	Adjusted for age, sex, other	1 or 2 / Never	3 / 37	19 / 78	0.52	(0.12 to 2.23)
					Leukemia	Any	Adjusted for age, sex, other	3 or 4 / Never	34 / 37	111 / 78	0.57	(0.27 to 1.21)[d]
					Leukemia	Any	Adjusted for age, sex, other	5 or 6 / Never	44 / 37	79 / 78	0.99	(0.45 to 2.18)
					Leukemia	Double vaccine (DT)	Adjusted for age, sex	Ever / Never	57 / 57	132 / 151	0.84	(0.52 to 1.37)
					Leukemia	Double vaccine (DT)	Adjusted for age, sex, other	Ever / Never	57 / 57	132 / 151	0.82	(0.44 to 1.54)
					Leukemia	Hep B	Adjusted for age, sex	Ever / Never	62 / 52	151 / 131	0.69	(0.41 to 1.17)
					Leukemia	Hep B	Adjusted for age, sex, other	Ever / Never	62 / 52	151 / 131	0.93	(0.49 to 1.76)[d]
					Leukemia	Measles	Adjusted for age, sex	Ever / Never	58 / 55	106 / 166	1.52	(0.95 to 2.45)

Table continues

Supplementary Table 2. Continued

Reference	Age Range	Exposure	Outcome	Comments	Cancer Site	Vaccine	Subsample	Model (Estimate / Ref)	No. of cases (Estimate / Ref)	No. of Controls or Expected (Estimate / Ref)	OR	95%CI
					Leukemia	Measles	Adjusted for age, sex, other	Ever / Never	58 / 55	106 / 166	1.87	(1.0 to 3.48)
					Leukemia	MMR	Adjusted for age, sex	Ever / Never	6 / 112	15 / 270	0.73	(0.27 to 2.0)
					Leukemia	MMR	Adjusted for age, sex, other	Ever / Never	6 / 112	15 / 270	0.8	(0.26 to 2.42)[d]
					Leukemia	Other vaccination before reference date	Adjusted for age, sex	Ever / Never	2 / 117	17 / 280	0.93	(0.18 to 4.91)
					Leukemia	Other	Adjusted for age, sex	Ever / Never	2 / 117	17 / 280	0.2	(0.05 to 0.92)
					Leukemia	Other	Adjusted for age, sex, other	Ever / Never	2 / 117	17 / 280	0.21	(0.04 to 1.12)
					Leukemia	Polio (Booster)	Adjusted for age, sex	Ever / Never	5 / 112	12 / 279	0.99	(0.32 to 3.04)
					Leukemia	Polio (Booster)	Adjusted for age, sex, other	Ever / Never	5 / 112	12 / 279	0.97	(0.26 to 3.59)
					Leukemia	Polio (Sip)	Adjusted for age, sex	Ever / Never	80 / 37	197 / 87	0.74	(0.45 to 1.23)
					Leukemia	Polio (Sip)	Adjusted for age, sex, other	Ever / Never	80 / 37	197 / 87	0.9	(0.47 to 1.74)[d]
					Leukemia	Routine	Adjusted for age, sex	Ever / Never	94 / 5	268 / 25	2.1	(0.75 to 5.83)
					Leukemia	Routine	Adjusted for age, sex, other	Ever / Never	94 / 5	268 / 25	2.38	(0.73 to 1.74)
					Leukemia	Rubella	Adjusted for age, sex	Ever / Never	1 / 114	5 / 257	0.78	(0.08 to 7.71)
					Leukemia	Rubella	Adjusted for age, sex, other	Ever / Never	1 / 114	5 / 257	0.5	(0.03 to 8.1)
					Leukemia	Triple vaccine (DTP)	Adjusted for age, sex	Ever / Never	80 / 38	197 / 88	0.73	(0.44 to 1.2)
					Leukemia	Triple vaccine (DTP)	Adjusted for age, sex, other	Ever / Never	80 / 38	197 / 88	1.01	(0.52 to 1.95)[d]
Groves, 1999[c]	0-15	Records		Adj: age, sex, race, birth year, day care attendance, parental education, family income; Mat: age, race, telepone number; population-based	ALL	Diphtheria		Ever / Never	431 / -	430 / -	0.75	(0.26 to 2.16)
					ALL	DTP		Ever / Never	424 / -	428 / -	0.66	(0.27 to 1.65)[d]
					ALL	Hib (polysaccharid)		Ever / Never	53 / -	50 / -	1.13	(0.64 to 1.98)
					ALL	Hib		Ever / Never	206 / -	232 / -	0.73	(0.5 to 1.06)[d]
					ALL	Hib (conjugate)		Ever / Never	153 / -	182 / -	0.57	(0.36 to 0.89)
					ALL	MMR		Ever / Never	395 / -	394 / -	1.19	(0.67 to 2.1)[d]
					ALL	Polio (Sip)		Ever / Never	429 / -	428 / -	1.05	(0.41 to 2.67)[d]
					ALL	Tetanus		Ever / Never	431 / -	430 / -	0.75	(0.26 to 2.16)
Schüz, 1999[c]	0-14	Self-report	Registry	Adj: socio-economic status; Mat: sex, birth year; population-based non-diseased; update Kaatsch 1996 & 1998	AL	Number (D, T, P, Polio, Mum, Mea, R, Sma, Men, routine)		0-3 / >6	150 / 341	70 / 458	3.2	(2.3 to 4.6)
					AL	Number (D, T, P, Polio, Mum, Mea, R, Sma, Men, routine)		4-6 / >6	501 / 341	464 / 458	1.5	(1.3 to 1.9)
					cALL	Number (D, T, P, Polio, Mum, Mea, R, Sma, Men, routine)		0-3 / >6	80 / 255	228 / 1196	2.1	(1.5 to 2.9)
					cALL	Number (D, T, P, Polio, Mum, Mea, R, Sma, Men, routine)		>6 / 0-3	255 / 80	1196 / 228	0.48	(0.34 to 0.67)[d,e]
					cALL	Number (D, T, P, Polio, Mum, Mea, R, Sma, Men, routine)		4-6 / >6	343 / 255	1155 / 1196	1.6	(1.3 to 1.9)

Table continues

Supplementary Table 2. Continued

Reference	Age Range	Exposure	Outcome	Comments	Cancer Site	Vaccine	Subsample	Model (Estimate / Ref)	No. of cases (Estimate / Ref)	No. of Controls or Expected (Estimate / Ref)	OR	95%CI
Von Kries, 2000[c]	0-16	Self-report	Registry	Adj: age, sex; Mat: age, sex; population-based without cancer; power only 50%; update Kaatsch 1996 & 1998 & Schüz 1999	Leukemia	BCG	Population controls	Ever / Never	129 / -	323 / -	0.88	(0.49 to 1.56)
					Leukemia	BCG	Local controls	Ever / Never	83 / -	83 / -	1.0	(0.38 to 2.66)
					Leukemia	BCG	State controls	Ever / Never	107 / -	133 / -	0.85	(0.43 to1.69)
					Leukemia	BCG	Local & state controls	Ever / Never	107 / -	273 / -	0.9	(0.51 to 1.61)[d]
					Tumor	BCG	Population controls	Ever / Never	130 / -	232 / -	0.9	(0.50 to 1.61)
					Tumor	BCG	Local & state controls	Ever / Never	109 / -	273 / -	0.61	(0.25 to 1.5)[d]
					Leukemia & tumor	BCG	Population controls	Ever / Never	259 / -	232 / -	0.89	(0.55 to 1.42)
					Leukemia & tumor	BCG	Local & state controls	Ever / Never	216 / -	273 / -	0.85	(0.51 to 1.41)
Krone, 2003[c]	0+	Self-report (some cards)	Hospital	Adj: age, sex, race, study centre, skin type, pigmented naevi, sunburns, freckling index; population-based	Melanoma	BCG	Vienna	Ever / Never	23 / -	32 / -	0.43	(0.17 to 1.0)
					Melanoma	BCG	Sofia	Ever / Never	100 / -	85 / -	0.43	(0.21 to 0.84)
					Melanoma	BCG	Tallinn	Ever / Never	59 / -	68 / -	0.67	(0.38 to 1.21)
					Melanoma	BCG	Dijon	Ever / Never	29 / -	25 / -	1.52	(0.72 to 3.18)
					Melanoma	BCG	Berlin	Ever / Never	23 / -	37 / -	0.42	(0.21 to 0.84)
					Melanoma	BCG	Dresden	Ever / Never	25 / -	45 -	0.42	(0.22 to 0.81)
					Melanoma	BCG	Tel Aviv	Ever / Never	24 / -	40 / -	0.37	(0.16 to 0.83)
					Melanoma	BCG	Padova	Ever / Never	22 / -	20 / -	1.2	(0.61 to 2.37)
					Melanoma	BCG	Total	Ever / Never	290 / -	367 / -	0.69	(0.52 to 0.92)[d]
					Melanoma	BCG & Smallpox		Ever / Never	271 / 63	341 / 37	0.41	(0.25 to 0.67)
					Melanoma	Influenza	Vienna	Ever / Never	10 / -	7 / -	0.79	(0.18 to 3.39)
					Melanoma	Influenza	Sofia	Ever / Never	8 / -	8 / -	0.74	(0.19 to 2.62)
					Melanoma	Influenza	Tallinn	Ever / Never	6 / -	3 / -	1.77	(0.4 to 9.37)
					Melanoma	Influenza	Dijon	Ever / Never	22 / -	16 / -	2.13	(0.6 to 8.24)
					Melanoma	Influenza	Berlin	Ever / Never	9 / -	11 / -	0.91	(0.28 to 2.89)
					Melanoma	Influenza	Dresden	Ever / Never	20 / -	29 / -	0.45	(0.19 to 1.05)
					Melanoma	Influenza	Tel Aviv	Ever / Never	12 / -	9 / -	0.83	(0.13 to 5.25)
					Melanoma	Influenza	Padova	Ever / Never	18 / -	27 / -	0.71	(0.33 to1.51)
					Melanoma	Influenza	Total	Ever / Never	105 / -	110 / -	0.91	(0.65 to 1.28)
					Melanoma	Only BCG		Ever / Never	19 / 63	26 / 37	0.4	(0.18 to 0.85)
					Melanoma	Smallpox	Vienna	Ever / Never	37 / -	37 / -	1	(0.33 to 2.99)
					Melanoma	Smallpox	Sofia	Ever / Never	104 / -	111 / -	0.34	(0.09 to 1.03)
					Melanoma	Smallpox	Tallinn	Ever / Never	95 / -	100 / -	0.16	(0.01 to 0.95)
					Melanoma	Smallpox	Dijon	Ever / Never	42 / -	45 / -	1.01	(0.42 to 2.41)
					Melanoma	Smallpox	Berlin	Ever / Never	50 / -	64 / -	0.2	(0.06 to 0.52)
					Melanoma	Smallpox	Dresden	Ever / Never	66 / -	74 / -	1.07	(0.31 to 3.87)
					Melanoma	Smallpox	Tel Aviv	Ever / Never	29 / -	31 / -	1.2	(0.54 to 2.67)
					Melanoma	Smallpox	Padova	Ever / Never	98 / -	102 / -	1.92	(0.17 to 2.15)
					Melanoma	Smallpox	Total	Ever / Never	521 / -	564 / -	0.65	(0.44 to 0.98)
					Melanoma	Smallpox		Ever / Never	250 / 63	223 / 37	0.6	(0.36 to 0.99)
Frentzel-Beyme, 2004[c]	8-25	Self-report	Registry	Mat: age, sex; population-based, hopital-based	Bone tumor	BCG		Ever / Never			0.7	(0.3 to 1.59)[d]
					Bone tumor	BCG	Female	Ever / Never			0.64	(0.19 to -)
					Bone tumor	BCG	Male	Ever / Never			0.75	(0.24 to 2.35)

Table continues

Supplementary Table 2. Continued

Reference	Age Range	Exposure	Outcome	Comments	Cancer Site	Vaccine	Subsample	Model (Estimate / Ref)	No. of cases (Estimate / Ref)	No. of Controls or Expected (Estimate / Ref)	OR	95%CI
					Bone tumor	BCG (repeated)		Ever / Never			0.66	(0.28 to 1.52)
					Bone tumor	BCG (repeated)	Female	Ever / Never			0.74	(0.22 to -)
					Bone tumor	BCG (repeated)	Male	Ever / Never			0.59	(0.19 to 2.49)
					Bone tumor	Chickenpox		Ever / Never			1.04	(0.39 to 2.77)
					Bone tumor	Chickenpox	Female	Ever / Never			0.68	(0.18 to 2.55)
					Bone tumor	Chickenpox	Male	Ever / Never			1.79	(0.36 to 2.55)
					Bone tumor	Chickenpox (repeated)		Ever / Never			0.77	(0.43 to 1.41)
					Bone tumor	Chickenpox (repeated)	Female	Ever / Never			1.02	(0.42 to 2.46)
					Bone tumor	Chickenpox (repeated)	Male	Ever / Never			0.62	(0.28 to 2.46)
					Bone tumor	Diphtheria		Ever / Never			0.43	(0.16 to 1.15)
					Bone tumor	Diphtheria	Female	Ever / Never			0.41	(0.1 to -)
					Bone tumor	Diphtheria	Male	Ever / Never			0.45	(0.11 to 1.64)
					Bone tumor	Diphtheria (repeated)		Ever / Never			0.88	(0.43 to 1.82)
					Bone tumor	Diphtheria (repeated)	Female	Ever / Never			1.49	(0.47 to -)
					Bone tumor	Diphtheria (repeated)	Male	Ever / Never			0.56	(0.21 to 4.71)
					Bone tumor	Pertussis		Ever / Never			1.07	(0.56 to 2.04)
					Bone tumor	Pertussis	Female	Ever / Never			0.56	(0.21 to -)
					Bone tumor	Pertussis	Male	Ever / Never			1.78	(0.71 to 1.54)
					Bone tumor	Pertussis (repeated)		Ever / Never			0.7	(0.39 to 1.27)
					Bone tumor	Pertussis (repeated)	Female	Ever / Never			0.33	(0.11 to -)
					Bone tumor	Pertussis (repeated)	Male	Ever / Never			1.16	(0.53 to 0.93)
					Bone tumor	Polio (repeated)		Ever / Never			2.16	(0.77 to 6.06)
					Bone tumor	Polio (repeated)	Female	Ever / Never			3.4	(0.69 to -)
					Bone tumor	Polio (repeated)	Male	Ever / Never			1.45	(0.37 to 16.8)
					Bone tumor	Polio		Ever / Never			0.52	(0.15 to1.86)
					Bone tumor	Polio	Female	Ever / Never			0.48	(0.06 to -)
					Bone tumor	Polio	Male	Ever / Never			0.55	(0.11 to 3.6)
					Bone tumor	Reaction after vaccination		Ever / Never			1.14	(0.45 to 2.86)
					Bone tumor	Reaction after vaccination	Female	Ever / Never			1.43	(0.38 to -)
					Bone tumor	Reaction after vaccination	Male	Ever / Never			0.93	(0.26 to 5.34)
					Bone tumor	Tetanus		Ever / Never			0.47	(0.15 to 1.49)
					Bone tumor	Tetanus	Female	Ever / Never			0.41	(0.08 to -)
					Bone tumor	Tetanus	Male	Ever / Never			0.55	(0.11 to 2.04)
					Bone tumor	Tetanus (repeated)		Ever / Never			1.35	(0.58 to 3.15)
					Bone tumor	Tetanus (repeated)	Female	Ever / Never			1.75	(0.47 to -)
					Bone tumor	Tetanus (repeated)	Male	Ever / Never			1.09	(0.35 to 6.56)

Table continues

Supplementary Table 2. Continued

Reference	Age Range	Exposure	Outcome	Comments	Cancer Site	Vaccine	Subsample	Model (Estimate / Ref)	No. of cases (Estimate / Ref)	No. of Controls or Expected (Estimate / Ref)	OR	95%CI
Ma, 2005[c]	0-15	Records	Registry	Adj: birth weight, day care attendence, family income, maternal education; Mat: age, sex, mother´s race, hispanic status; population-based	Leukemia	Hep B		Each additional dose	323 / -	409 / -	0.97	(0.77 to 1.23)[d]
					Leukemia	Hep B	Vaccination during infancy	3+ / 1-2	323 / -	409 / -	1.41	(0.9 to 2.2)[d]
					Leukemia	Hep B	Born <1995	Each additional dose	308 / -	392 / -	1.06	(0.81 to 1.38)
					Leukemia	Hep B	Vaccination during infancy, born <1995	3+ / 1-2	308 / -	392 / -	1.81	(0.99 to 3.3)
					Leukemia	Hep B	Born 1995+	Each additional dose	308 / -	392 / -	0.69	(0.41 to 1.16)
					Leukemia	Hep B	Vaccination during infancy, born 1995+	3+ / 1-2	308 / -	392 / -	1.08	(0.55 to 2.14)
					Leukemia	Hib		Each additional dose	323 / -	409 / -	0.81	(0.68 to 0.96)
					Leukemia	Hib		3+ / 1-2	323 / -	409 / -	0.55	(0.32 to 0.94)[d]
					Leukemia	Hib	Born <1995	Each additional dose	308 / -	392 / -	0.78	(0.63 to 0.96)
					Leukemia	Hib	Born <1995	3+ / 1-2	308 / -	392 / -	0.41	(0.21 to 0.81)
					Leukemia	Hib	Born 1995+	Each additional dose	308 / -	392 / -	0.86	(0.61 to 1.22)
					Leukemia	Hib	Born 1995+	3+ / 1-2	308 / -	392 / -	0.86	(0.32 to 2.31)
					Leukemia	MMR		Each additional dose	323 / -	409 / -	1.06	(0.69 to 1.63)[d]
					Leukemia	MMR	Born <1995	Each additional dose	308 / -	392 / -	0.94	(0.57 to 1.53)
					Leukemia	MMR	Born 1995+	Each additional dose	308 / -	392 / -	0.79	(0.35 to 1.78)
					Leukemia	Polio		Each additional dose	323 / -	409 / -	1.14	(0.88 to 1.47)[d]
					Leukemia	Polio	Born <1995	Each additional dose	308 / -	392 / -	1.28	(0.94 to 1.74)
					Leukemia	Polio	Born 1995+	Each additional dose	308 / -	392	0.77	(0.45 to 1.32)
					Leukemia	DPT		Each additional dose	323 / -	409 / -	0.97	(0.74 to 1.28)[d]
					Leukemia	DPT	Born <1995	Each additional dose	308 / -	392 / -	1.06	(0.78 to 1.46)
					Leukemia	DPT	Born 1995+	Each additional dose	308 / -	392 / -	0.7	(0.39 to 1.28)
					ALL	DPT		Each additional dose	282 / -	360 / -	0.96	(0.72 to 1.28)[d]
					ALL	DPT	Born <1995	Each additional dose	270 / -	346 / -	1.07	(0.77 to 1.49)
					ALL	DPT	Born 1995+	Each additional dose	270 / -	346 / -	0.65	(0.34 to 1.23)
					ALL	Hep B		Each additional dose	282 / -	360 / -	1.01	(0.78 to 1.31)[d]
					ALL	Hep B	Vaccination during infancy	3+ / 1-2	282 / -	360 / -	1.42	(0.88 to 2.27)
					ALL	Hep B	Born <1995	Each additional dose	282 / -	360 / -	1.08	(0.8 to 1.46)
					ALL	Hep B	Vaccination during infancy, born <1995	3+ / 1-2	270 / -	346 / -	1.76	(0.94 to 3.3)
					ALL	Hep B	Born 1995+	Each additional dose	270 / -	346 / -	0.75	(0.43 to 1.33)

Table continues

Supplementary Table 2. Continued

Reference	Age Range	Exposure	Outcome	Comments	Cancer Site	Vaccine	Subsample	Model (Estimate / Ref)	No. of cases (Estimate / Ref)	No. of Controls or Expected (Estimate / Ref)	OR	95%CI
					ALL	Hep B	Vaccination during infancy, born 1995+	3+ / 1-2	270 / -	346 / -	1	(0.5 to 2.18)
					ALL	Hib		Each additional dose	282 / -	360 / -	0.81	(0.66 to 0.98)[d]
					ALL	Hib		3+ / 1-2	282 / -	360 / -	0.61	(0.34 to 1.1)[d]
					ALL	Hib	Born <1995	Each additional dose	270 / -	346 / -	0.79	(0.63 to 0.99)
					ALL	Hib	Born <1995	3+ / 1-2	270 / -	346 / -	0.48	(0.23 to 0.99)
					ALL	Hib	Born 1995+	Each additional dose	270 /-	346 / -	0.81	(0.55 to 1.19)
					ALL	Hib	Born 1995+	3+ / 1-2	270 / -	346 / -	0.84	(0.29 to 2.42)
					ALL	MMR		Each additional dose	282 / -	360 / -	0.87	(0.55 to 1.37)[d]
					ALL	MMR	Born <1995	Each additional dose	282 / -	360 / -	0.95	(0.56 to 1.6)
					ALL	MMR	Born 1995+	Each additional dose	270 / -	346 / -	0.65	(0.24 to 1.72)
					ALL	Polio		Each additional dose	308 / -	392 / -	1.08	(0.82 to 1.41)[d]
					ALL	Polio	Born <1995	Each additional dose	270 / -	346 / -	1.21	(0.88 to 1.68)
					ALL	Polio	Born 1995+	Each additional dose	270 / -	346 / -	0.72	(0.4 to 1.26)
Mallol-Mesnard, 2007[c]	0-15	Vaccina-tion card	Registry	Adj: age, sex, birth order, maternal and paternal educational level, degree of urbanization; Mat: age, sex; population-based	AL	Any	Number of injections	0 / >9	4 / 176	13 / 446	0.7	(0.2 to 2.2)
					AL	Any	Number of injections	1-3 / >9	17 / 176	57 / 446	0.7	(0.4 to 1.3)
					AL	Any	Number of injections	4-6 / >9	246 / 176	380 / 446	1.3	(1.0 to 1.7)
					AL	Any	Number of injections	7-9 / >9	229 / 176	507 / 446	1.0	(0.8 to 1.3)
					AL	Any	Number of injections age <0.5	0 / >3	34 / 310	73 / 684	1.1	(0.7 to 1.7)
					AL	Any	Number of injections age <0.5	1-2 / >3	121 / 310	266 / 684	1.1	(0.9 to 1.5)
					AL	Any	Number of injections age <0.5	3 / >3	207 / 310	380 / 684	1.2	(1.0 to 1.6)
					AL	Any	Number of injections age <0.5	>3 / 3	310 / 207	684 / 380	0.83	(0.63 to 1.0)[d,e]
					AL	Any	Number of vaccine doses	0-14 / >24	38 / 352	104 / 797	0.8	(0.5 to 1.2)
					AL	Any	Number of vaccine doses	15-19 / >24	85 / 352	180 / 797	1.1	(0.8 to 1.6)
					AL	Any	Number of vaccine doses	20-24 / >24	197 / 352	322 / 797	1.1	(0.9 to 1.5)
					AL	Any	Number of vaccine doses age <0.5	0 / >9	34 / 538	73 / 1052	1.0	(0.6 to 1.5)
					AL	Any	Number of vaccine doses age <0.5	1-9 / >9	100 / 538	278 / 1052	0.9	(0.7 to 1.1)
					AL	BCG	Vaccination age <0.5.	Ever / Never	624 / 48	1309 / 94	0.93	(0.65 to 1.34)[a]
					AL	Diphtheria	Vaccination age <0.5.	Ever / Never	663 / 9	1365 / 38	2.05	(0.99 to 4.27)[a]
					AL	Hep B	Vaccination age <0.5.	Ever / Never	250 / 422	510 / 893	1.04	(0.86 to 1.26)[a]
					AL	Hib	Vaccination age <0.5.	Ever / Never	582 / 90	1132 / 271	1.55	(1.20 to 2.00)[a]
					AL	Measles	Vaccination age <0.5.	Ever / Never	541 / 55	1110 / 119	1.05	(0.75 to 1.48)[a]
					AL	Meningo-coccus	Vaccination age <0.5.	Ever / Never	31 / 641	71 / 1332	0.91	(0.59 to 1.40)[a]
					AL	Mumps	Vaccination age <0.5.	Ever / Never	539 / 57	1103 / 126	1.08	(0.78 to 1.50)[a]

Table continues

Supplementary Table 2. Continued

Reference	Age Range	Exposure	Outcome	Comments	Cancer Site	Vaccine	Subsample	Model (Estimate / Ref)	No. of cases (Estimate / Ref)	No. of Controls or Expected (Estimate / Ref)	OR	95%CI
					AL	Pertussis	Vaccination age <0.5.	Ever / Never	646 / 26	1325 / 78	1.46	(0.93 to 2.30)[a]
					AL	Pneumococcus	Vaccination age <0.5.	Ever / Never	30 / 642	101 / 1302	0.6	(0.40 to 0.92)[a]
					AL	Polio	Vaccination age <0.5.	Ever / Never	665 / 7	1366 / 37	2.57	(1.14 to 5.80)[a]
					AL	Rubella	Vaccination age <0.5.	Ever / Never	540 / 56	1104 / 125	1.09	(0.78 to 1.52)[a]
					AL	Tetanus	Vaccination age <0.5.	Ever / Never	664 / 8	1368 / 35	2.12	(0.98 to 4.60)[a]
					ALL	Any	Number of injections	0 / >9	4 / 148	13 / 446	0.8	(0.3 to 2.7)
					ALL	Any	Number of injections	1-3 / >9	15 / 148	57 / 446	0.8	(0.4 to 1.5)
					ALL	Any	Number of injections	4-6 / >9	223 / 148	380 / 446	1.4	(1.0 to 1.8)
					ALL	Any	Number of injections	7-9 / >9	197 / 148	507 / 446	1.0	(0.7 to 1.3)
					ALL	Any	Number of injections age <0.5	0 / >3	27 / 278	73 / 684	1.0	(0.6 to 1.6)
					ALL	Any	Number of injections age <0.5	1-2 / >3	97 / 278	266 / 684	1.0	(0.8 to 1.3)
					ALL	Any	Number of injections age <0.5	3 / >3	185 / 278	380 / 684	1.2	(1.0 to 1.6)
					ALL	Any	Number of injections age <0.5	>3 / 3	278 / 185	684 / 380	0.83	(0.63 to 1.00)[d,e]
					ALL	Any	Number of vaccine doses	0-14 / >24	32 / 302	104 / 797	0.8	(0.5 to 1.2)
					ALL	Any	Number of vaccine doses	15-19 / >24	72 / 302	180 / 797	1.2	(0.8 to 1.7)
					ALL	Any	Number of vaccine doses	20-24 / >24	181 / 302	322 / 797	1.2	(0.9 to 1.5)
					ALL	Any	Number of vaccine doses age <0.5	0 / >9	27 / 475	73 / 1052	0.9	(0.6 to 1.4)
					ALL	Any	Number of vaccine doses age <0.5	1-9 / >9	85 / 475	278 / 1052	0.8	(0.6 to 1.1)
					ALL	BCG	Vaccination age <0.5.	Ever / Never	548 / 39	1309 / 94	1.01	(0.69 to 1.48)[a]
					ALL	Diphtheria	Vaccination age <0.5.	Ever / Never	578 / 9	1365 / 38	1.79	(0.86 to 3.72)[a]
					ALL	Hep B	Vaccination age <0.5.	Ever / Never	211 / 376	510 / 893	0.98	(0.80 to 1.20)[a]
					ALL	Hib	Vaccination age <0.5.	Ever / Never	512 / 75	1132 / 271	1.63	(1.24 to 2.15)[a]
					ALL	Measles	Vaccination age <0.5.	Ever / Never	480 / 46	1110 / 119	1.12	(0.78 to 1.60)[a]
					ALL	Meningo-coccus	Vaccination age <0.5.	Ever / Never	28 / 559	71 / 1332	0.94	(0.60 to 1.47)[a]
					ALL	Mumps	Vaccination age <0.5.	Ever / Never	478 / 48	1103 / 126	1.14	(0.80 to 1.61)[a]
					ALL	Pertussis	Vaccination age <0.5.	Ever / Never	565 / 22	1325 / 78	1.51	(0.93 to 2.45)[a]
					ALL	Pneumococcus	Vaccination age <0.5.	Ever / Never	24 / 563	101 / 1302	0.55	(0.35 to 0.87)[a]
					ALL	Polio	Vaccination age <0.5.	Ever / Never	580 / 7	1366 / 37	2.24	(0.99 to 5.06)[a]
					ALL	Rubella	Vaccination age <0.5.	Ever / Never	479 / 47	1104 / 125	1.15	(0.81 to 1.64)[a]
					ALL	Tetanus	Vaccination age <0.5.	Ever / Never	579 / 8	1368 / 35	1.85	(0.85 to 4.02)[a]
					AML	Any	Number of injections	0 / >9	0 / 21	13 / 446		
					AML	Any	Number of injections	1-3 / >9	2 / 21	57 / 446	0.5	(0.1 to 2.5)
					AML	Any	Number of injections	4-6 / >9	18 / 21	380 / 446	0.9	(0.4 to 1.9)
					AML	Any	Number of injections	7-9 / >9	30 / 21	507 / 446	1.3	(0.7 to 2.3)
					AML	Any	Number of injections age <0.5	0 / >3	6 / 26	73 / 684	2.2	(0.9 to 5.7)
					AML	Any	Number of injections age <0.5	1-2 / >3	21 / 26	266 / 684	2.3	(1.2 to 4.2)
					AML	Any	Number of injections age <0.5	3 / >3	18 / 26	380 / 684	1.3	(0.7 to 2.4)

Table continues

Supplementary Table 2. Continued

Reference	Age Range	Exposure	Outcome	Comments	Cancer Site	Vaccine	Subsample	Model (Estimate / Ref)	No. of cases (Estimate / Ref)	No. of Controls or Expected (Estimate / Ref)	OR	95%CI
					AML	Any	Number of vaccine doses	0-14 / >24	5 / 42	104 / 797	0.7	(0.3 to 2.1)
					AML	Any	Number of vaccine doses	15-19 / >24	12 / 42	180 / 797	0.9	(0.3 to 2.2)
					AML	Any	Number of vaccine doses	20-24 / >24	12 / 42	322 / 797	0.7	(0.4 to 1.5)
					AML	Any	Number of vaccine doses age <0.5	0 / >9	6 / 51	73 / 1052	1.7	(0.7 to 4.2)
					AML	Any	Number of vaccine doses age <0.5	1-9 / >9	14 / 51	278 / 1052	1.1	(0.6 to 2.1)
					AML	BCG	Vaccination age <0.5.	Ever / Never	63 / 8	1309 / 94	0.57	(0.26 to 1.22)[a]
					AML	Diphtheria	Vaccination age <0.5.	Ever / Never	71 / 0	1365 / 38		
					AML	Hep B	Vaccination age <0.5.	Ever / Never	31 / 40	510 / 893	1.36	(0.84 to 2.20)[a]
					AML	Hib	Vaccination age <0.5.	Ever / Never	58 / 13	1132 / 271	1.07	(0.58 to 1.98)[a]
					AML	Measles	Vaccination age <0.5.	Ever / Never	50 / 8	1110 / 119	0.67	(0.31 to 1.45)[a]
					AML	Meningo-coccus	Vaccination age <0.5.	Ever / Never	2 / 69	71 / 1332	0.54	(0.13 to 2.26)[a]
					AML	Mumps	Vaccination age <0.5.	Ever / Never	50 / 8	1103 / 126	0.71	(0.33 to 1.54)[a]
					AML	Pertussis	Vaccination age <0.5.	Ever / Never	67 / 4	1325 / 78	0.99	(0.35 to 2.77)[a]
					AML	Pneumococcus	Vaccination age <0.5.	Ever / Never	5 / 66	101 / 1302	0.98	(0.38 to 2.48)[a]
					AML	Polio	Vaccination age <0.5.	Ever / Never	71 / 0	1366 / 37		
					AML	Rubella	Vaccination age <0.5.	Ever / Never	50 / 8	1104 / 125	0.71	(0.33 to 1.53)[a]
					AML	Tetanus	Vaccination age <0.5.	Ever / Never	71 / 0	1368 / 35		
Daniels, 2008	<16	Self-report	Hospital	Exc: rare vacc; Mat: age, area; Adj: age, area, sex, maternal education, household income; population-based	Wilms tumor	Any	Adjusted for age, area	Ever / Never	14 / 502	16 / 498	0.8	(0.4 to 1.7)
					Wilms tumor	Any	Adjusted for age, area, other	Ever / Never	14 / 502	16 / 498	0.9	(0.4 to 1.8)
MacArthur, 2008[c]	0-15	Vaccina-tion card	Cancer registry	Adj: race, family income, maternal education & age at birth, number of residences since birth; Mat: age, sex area; population-based	Leukemia	BCG		Ever / Never	15 / 356	15 / 368	0.97	(0.43 to 2.2)[d]
					Leukemia	Diphtheria		Ever / Never	373 / 15	386 / 13	0.85	(0.29 to 2.49)
					Leukemia	Hep		Ever / Never	8 / 368	7 / 378	1.09	(0.34 to 3.52)[d]
					Leukemia	Measles	Vaccination age >1	Ever / Never			0.49	(0.2 to 1.18)
					Leukemia	Measles		Ever / Never	338 / 49	350 / 48	0.88	(0.41 to 1.9)
					Leukemia	Mumps	Vaccination age >1	Ever / Never			0.18	(0.18 to 1.02)
					Leukemia	Mumps		Ever / Never	335 / 50	349 / 49	0.83	(0.39 to 1.75)
					Leukemia	Other vaccination		Ever / Never	168 / 154	207 / 178	0.81	(0.58 to 1.13)[d]
					Leukemia	Pertussis		Ever / Never	372 / 16	383 / 15	0.71	(0.27 to 1.85)
					Leukemia	Polio		Ever / Never	371 / 15	384 / 15	0.9	(0.35 to 2.29)[d]
					Leukemia	Rubella	Vaccination age >1	Ever / Never			0.23	(0.23 to 1.12)
					Leukemia	Rubella		Ever / Never	334 / 54	346 / 51	0.85	(0.42 to 1.7)
					Leukemia	Tetanus		Ever / Never	373 / 15	386 / 13	0.74	(0.27 to 2.03)
					ALL	BCG		Ever / Never	15 / 356	15 / 368	1.1	(0.48 to 2.51)
					ALL	Diphtheria		Ever / Never	333 / 7	386 / 13	0.99	(0.3 to 3.27)
					ALL	Hep		Ever / Never	7 / 321	7 / 378	1.08	(0.32 to 3.68)[d]
					ALL	Measles		Ever / Never	304 / 35	350 / 48	0.96	(0.43 to 2.13)[d]
					ALL	Mumps		Ever / Never	301 / 37	349 / 49	0.86	(0.4 to 1.86)

Table continues

Supplementary Table 2. Continued

Reference	Age Range	Exposure	Outcome	Comments	Cancer Site	Vaccine	Subsample	Model (Estimate / Ref)	No. of cases (Estimate / Ref)	No. of Controls or Expected (Estimate / Ref)	OR	95%CI
					ALL	Other vaccination		Ever / Never	184 / 184	207 / 178	0.81	(0.58 to 1.15)
					ALL	Pertussis		Ever / Never	331 / 9	383 / 15	0.84	(0.29 to 2.42)
					ALL	Polio		Ever / Never	329 / 9	384 / 15	1.01	(0.37 to 2.8)[d]
					ALL	Rubella		Ever / Never	301 / 39	346 / 51	0.86	(0.42 to 1.78)
					ALL	Tetanus		Ever / Never	332 / 8	386 / 13	0.85	(0.28 to 2.57)
Sankaran, 2016	0-20	Self-report		Exc: rare cancer; Adj: family income, maternal education; Mat: age, sex, race; population-based; no controls for 8% cases	Rhabdomyosarcoma	DPT	Unadjusted	Incomplete / Complete	95 / 213	67 / 246	1.66	(1.15 to 2.4)
					Rhabdomyosarcoma	DPT	Unadjusted	Never / Complete	14 / 213	9 / 246	1.91	(0.81 to 4.40)
					Rhabdomyosarcoma	DPT	Adjusted	Incomplete / Complete	95 / 213	67 / 246	1.56	(1.06 to 2.29)
					Rhabdomyosarcoma	DPT	Adjusted	Never / Complete	14 / 213	9 / 246	1.74	(0.73 to 4.15)
					Rhabdomyosarcoma	Incomplete vaccination	Unadjusted	Ever / Never	47 / 257	12 / 304	4.5	(2.27 to 8.93)
					Rhabdomyosarcoma	Incomplete vaccination	Adjusted	Ever / Never	47 / 257	12 / 304	5.3	(2.47 to 11.33)
					Rhabdomyosarcoma	MMR	Unadjusted	Ever / Never	81 / 241	61 / 261	1.57	(1.02 to 2.4)
					Rhabdomyosarcoma	MMR	Adjusted	Ever / Never	81 / 241	61 / 261	1.43	(0.92 to 2.21)
					Rhabdomyosarcoma	Polio	Unadjusted	Incomplete / Complete	95 / 213	67 / 246	1.52	(0.93 to 2.48)
					Rhabdomyosarcoma	Polio	Unadjusted	Never / Complete	14 / 213	9 / 246	1.69	(0.95 to 3.03)
					Rhabdomyosarcoma	Polio	Adjusted	Incomplete / Complete	95 / 213	67 / 246	1.42	(0.86 to 2.35)
					Rhabdomyosarcoma	Polio	Adjusted	Never / Complete	14 / 213	9 / 246	1.87	(1.00 to 3.50)
Figueroa, 2019	1-15	Self-report	Registry	Adj: sex, birth year, SES	ALL	Complete vaccination	Unadjusted, one exposure at a time	Complete / Incomplete	218/22	550/26	0.47	(0.26 to 0.84)
					ALL	Complete vaccination	Adjusted, one exposure at a time	Complete / Incomplete	218/22	550/26	0.45	(0.24 to 0.83)
					ALL	Complete vaccination	Adjusted, All exposures in the same model	Complete / Incomplete	218/22	550/26	0.48	(0.26 to 0.89)[d]

Table continues

Supplementary Table 2C. Estimates of ecological studies included in the systematic review

Reference	Age Range	Exposure	Outcome	Comments	Cancer Site	Vaccine	Subsample	Model (Estimate / Ref)	No. of cases (Estimate / Ref)	No. of Controls or Expected (Estimate / Ref)	OR	95%CI
Fraumeni, 1963	>1	Records		Exc: without complete estimates; cancer death	Cancer	Polio						
Waaler, 1970	0-43			Exc: without complete estimates; age specific mortality; letter to editor; original study of Villumsen 2009	Leukemia	BCG						
Berkeley, 1971	0-14		Registry	Exc: without complete estimates; outcome cancer deaths; letter to editor	Leukemia	BCG						
Hems, 1971	>10			Exc: without complete estimates; cancer deaths; letter to editor; latency considered	Leukemia	BCG						
Kinlen, 1971		Registry	Registry	Exc: without complete estimates; standardized incidence ratio	Leukemia	BCG	Quebec, 1950	Ever / Never			0.93	
					Leukemia	BCG	Quebec, 1951	Ever / Never			0.90	
					Leukemia	BCG	Quebec, 1952	Ever / Never			0.83	
					Leukemia	BCG	Quebec, 1953	Ever / Never			1.26	
					Leukemia	BCG	Quebec, 1954	Ever / Never			1.05	
					Leukemia	BCG	Quebec, 1955	Ever / Never			1.02	
					Leukemia	BCG	Quebec, 1956	Ever / Never			0.70	
					Leukemia	BCG	Quebec, 1957	Ever / Never			1.25	
					Leukemia	BCG	Quebec, 1958	Ever / Never			0.83	
					Leukemia	BCG	Quebec, 1959	Ever / Never			0.92	
					Leukemia	BCG	Quebec, 1960	Ever / Never			1.02	
					Leukemia	BCG	Quebec, 1961	Ever / Never			1.27	
					Leukemia	BCG	Quebec, 1962	Ever / Never			1.82	
					Leukemia	BCG	Quebec, 1963	Ever / Never			1.04	
					Leukemia	BCG	Quebec, 1964	Ever / Never			0.93	
					Leukemia	BCG	Quebec, 1965	Ever / Never			1.10	
					Leukemia	BCG	Quebec, 1966	Ever / Never			1.30	
					Leukemia	BCG	Quebec, 1967	Ever / Never			1.28	
					Leukemia	BCG	Quebec, 1968	Ever / Never			0.86	
					Leukemia	BCG	Quebec, 1969	Ever / Never			0.75	
					Leukemia	BCG	Quebec, 1950-54	Ever / Never			1.00	

Table continues

Supplementary Table 2. Continued

Reference	Age Range	Exposure	Outcome	Comments	Cancer Site	Vaccine	Subsample	Model (Estimate / Ref)	No. of cases (Estimate / Ref)	No. of Controls or Expected (Estimate / Ref)	OR	95%CI
					Leukemia	BCG	Quebec, 1955-59	Ever / Never			0.93	
					Leukemia	BCG	Quebec, 1960-64	Ever / Never			1.20	
					Leukemia	BCG	Quebec, 1965-69	Ever / Never			1.07	
					Leukemia	BCG	Quebec, 1950-69	Ever / Never			1.05	
					Leukemia	BCG	Glasgow, 1951-54	Ever / Never			1.06	
					Leukemia	BCG	Glasgow, 1955-59	Ever / Never			0.86	
					Leukemia	BCG	Glasgow, 1960-64	Ever / Never			0.97	
					Leukemia	BCG	Glasgow, 1965-69	Ever / Never			1.16	
					Leukemia	BCG	Glasgow, 1951-69	Ever / Never			1.00	
Hofmann, 1977	0-24			Exc: without complete estimates; article in Polish	Leukemia	BCG						
Ambrosch, 1978	0-5			Exc: without complete estimates; protection rate	Leukemia	BCG		Ever / Never			0.77	
Sinniah, 1978	Children		Hospital	Exc: without complete estimates; comparison of individual vaccination rates of cases & aggregated controls	ALL	BCG	Vaccination rate cases (%)	Ever / Never	34		Rate: 89.2%	
					ALL	BCG	Vaccination rate general population (%)	Ever / Never			Rate: 82.4%	
Skegg, 1978	13-23	Registry	Registry	Exc: without complete estimates; Adj: routine vaccination	Non-Hodgkin lymphoma	BCG	North Island, early cohort	Ever / Never	12	15.8	Ratio: 0.76	
					Non-Hodgkin lymphoma	BCG	South Island, early cohort	Ever / Never	6	7.1	Ratio: 0.85	
					Non-Hodgkin lymphoma	BCG	North Island, late cohort	Ever / Never	20	15	Ratio: 1.33	
					Non-Hodgkin lymphoma	BCG	North Island, early cohort	Ever / Never	2	6.8	Ratio: 0.29	
Nilsson, 1979	<2		Registry	Exc: without complete estimates; letter to editor	Cancer	BCG			37	194314		
Pagaoa, 2011[c]	2-17	Registry	Registry	Adj: age, sex, race, birth weight, birth year, birth type, birth order, premature birth, maternal education, maternal marital status, prior births, diabetes, preterm labor, tobacco use, and alcohol use, mother age at birth; Mat: sex, birth year; 1:4; population-based without cancer	Cancer	Chickenpox	County-level vaccination rates; 1 dose	Ever / Never			1.03	(0.92 to 1.16)
					Cancer	DTP	Public health region vaccination rates; 4 doses	Ever / Never	2800 / -		0.92	(0.80 to 1.07)
					Cancer	DTP	County-level vaccination rates; 4 doses	Ever / Never	1707 / -	6828 / -	1.20	(0.90 to 1.60)
					Cancer	DTP, Polio (IPV) & MMR	Public health region vaccination rates; 4 doses DTP, 3 doses Polio (IPV), 1 dose MMR	Ever / Never			0.90	(0.80 to 1.03)
					Cancer	DTP, Polio (IPV) & MMR	County-level vaccination rates; 4 doses DTP, 3 doses Polio (IPV), 1 dose MMR	Ever / Never			1.00	(0.89 to 1.11)
					Cancer	DTP, Polio (IPV), MMR & Hib	Public health region vaccination rates; 4 doses DTP, 3 doses Polio (IPV), 1 dose MMR, 3 doses Hib	Ever / Never			0.98	(0.87 to 1.11)

Table continues

Supplementary Table 2. Continued

Reference	Age Range	Exposure	Outcome	Comments	Cancer Site	Vaccine	Subsample	Model (Estimate / Ref)	No. of cases (Estimate / Ref)	No. of Controls or Expected (Estimate / Ref)	OR	95%CI
					Cancer	DTP, Polio (IPV), MMR & Hib	County-level vaccination rates; 4 doses DTP, 3 doses Polio (IPV), 1 dose MMR, 3 doses Hib	Ever / Never			0.90	(0.74 to 1.09)
					Cancer	DTP, Polio (IPV), MMR, Hib, Hep & Chickenpox	County-level vaccination rates; 4 doses DTP, 3 doses Polio (IPV), 1 dose MMR, 3 doses Hib, 3 doses Hep, 1 dose Chickenpox	Ever / Never			0.98	(0.88 to 1.10)
					Cancer	Hep B	County-level vaccination rates; 3 doses	Ever / Never			0.81	(0.67 to 0.98)
					Cancer	Hib	Public health region vaccination rates; 3 doses	Ever / Never			0.84	(0.70 to 1.00)
					Cancer	Hib	County-level vaccination rates; 3 doses	Ever / Never			0.92	(0.82 to 1.04)
					Cancer	MMR	Public health region vaccination rates; 1 dose	Ever / Never			0.92	(0.81 to 1.02)
					Cancer	MMR	County-level vaccination rates; 1 dose	Ever / Never			1.1	(0.84 to 1.45)
					Cancer	Polio (IPV)	Public health region vaccination rates; 3 doses	Ever / Never			0.93	(0.81 to 1.07)[d]
					Cancer	Polio (IPV)	County-level vaccination rates; 3 doses	Ever / Never			0.88	(0.74 to 1.05)
					ALL	Chickenpox	County-level vaccination rates; 1 dose	Ever / Never			1.07	(0.78 to 1.47)
					ALL	DTP	Public health region vaccination rates; 4 doses	Ever / Never	895 / -		0.82	(0.63 to 1.06)[d]
					ALL	DTP	County-level vaccination rates; 4 doses	Ever / Never	547 / -		1.02	(0.61 to 1.72)
					ALL	DTP, Polio (IPV) & MMR	Public health region vaccination rates; 4 doses DTP, 3 doses Polio (IPV), 1 dose MMR	Ever / Never			0.77	(0.60 to 1.00)
					ALL	DTP, Polio (IPV) & MMR	County-level vaccination rates; 4 doses DTP, 3 doses Polio (IPV), 1 dose MMR	Ever / Never			0.73	(0.51 to 1.06)
					ALL	DTP, Polio (IPV), MMR & Hib	Public health region vaccination rates; 4 doses DTP, 3 doses Polio (IPV), 1 dose MMR, 3 doses Hib	Ever / Never			1.04	(0.74 to 1.47)
					ALL	DTP, Polio (IPV), MMR & Hib	County-level vaccination rates; 4 doses DTP, 3 doses Polio (IPV), 1 dose MMR, 3 doses Hib	Ever / Never			0.62	(0.44 to 0.87)
					ALL	DTP, Polio (IPV), MMR, Hib, Hep & Chickenpox	County-level vaccination rates; 4 doses DTP, 3 doses Polio (IPV), 1 dose MMR, 3 doses Hib, 3 doses Hep, 1 dose Chickenpox	Ever / Never			0.77	(0.50 to 1.17)
					ALL	Hep B	County-level vaccination rates; 3 doses	Ever / Never			0.63	(0.46 to 0.88)[d]
					ALL	Hib	Public health region vaccination rates; 3 doses	Ever / Never			0.58	(0.42 to 0.82)[d]

Table continues

Supplementary Table 2. Continued

Reference	Age Range	Exposure	Outcome	Comments	Cancer Site	Vaccine	Subsample	Model (Estimate / Ref)	No. of cases (Estimate / Ref)	No. of Controls or Expected (Estimate / Ref)	OR	95%CI
					ALL	Hib	County-level vaccination rates; 3 doses	Ever / Never			0.76	(0.54 to 1.08)
					ALL	MMR	Public health region vaccination rates; 1 dose	Ever / Never			0.87	(0.71 to 1.08)[d]
					ALL	MMR	County-level vaccination rates; 1 dose	Ever / Never			1.00	(0.51 to 1.39)
					ALL	Polio (IPV)	Public health region vaccination rates; 3 doses	Ever / Never			0.83	(0.63 to 1.09)[d]
					ALL	Polio (IPV)	County-level vaccination rates; 3 doses	Ever / Never			0.67	(0.49 to 0.92)
					Medulloblastoma	Chickenpox	County-level vaccination rates; 1 dose	Ever / Never			0.90	(0.54 to 1.51)
					Medulloblastoma	DTP	Public health region vaccination rates; 4 doses	Ever / Never	114 / -		1.11	(0.71 to 1.73)
					Medulloblastoma	DTP	County-level vaccination rates; 4 doses	Ever / Never	70 / -		1.43	(0.44 to 4.63)
					Medulloblastoma	DTP, Polio (IPV) & MMR	Public health region vaccination rates; 4 doses DTP, 3 doses Polio (IPV), 1 dose MMR	Ever / Never			1.39	(0.85 to 2.27)
					Medulloblastoma	DTP, Polio (IPV) & MMR	County-level vaccination rates; 4 doses DTP, 3 doses Polio (IPV), 1 dose MMR	Ever / Never			1.14	(0.60 to 2.18)
					Medulloblastoma	DTP, Polio (IPV), MMR & Hib	Public health region vaccination rates; 4 doses DTP, 3 doses Polio (IPV), 1 dose MMR, 3 doses Hib	Ever / Never			1.46	(0.90 to 2.36)
					Medulloblastoma	DTP, Polio (IPV), MMR & Hib	County-level vaccination rates; 4 doses DTP, 3 doses Polio (IPV), 1 dose MMR, 3 doses Hib	Ever / Never			1.58	(0.76 to 3.30)
					Medulloblastoma	DTP, Polio (IPV), MMR, Hib, Hep & Chickenpox	County-level vaccination rates; 4 doses DTP, 3 doses Polio (IPV), 1 dose MMR, 3 doses Hib, 3 doses Hep, 1 dose Chickenpox	Ever / Never			1.12	(0.58 to 2.17)
					Medulloblastoma	Hep B	County-level vaccination rates; 3 doses	Ever / Never			1.39	(0.67 to 2.91)
					Medulloblastoma	Hib	Public health region vaccination rates; 3 doses	Ever / Never			1.45	(0.75 to 2.80)
					Medulloblastoma	Hib	County-level vaccination rates; 3 doses	Ever / Never			1.62	(1.00 to 2.62)
					Medulloblastoma	MMR	Public health region vaccination rates; 1 dose	Ever / Never			1.10	(0.70 to 1.72)
					Medulloblastoma	MMR	County-level vaccination rates; 1 dose	Ever / Never			1.20	(0.37 to 3.88)
					Medulloblastoma	Polio (IPV)	Public health region vaccination rates; 3 doses	Ever / Never			1.49	(0.89 to 2.52)[d]
					Medulloblastoma	Polio (IPV)	County-level vaccination rates; 3 doses	Ever / Never			1.47	(0.73 to 2.96)
					Non-Hodgkin lymphoma	Chickenpox	County-level vaccination rates; 1 doses	Ever / Never			0.97	(0.58 to 1.62)
					Non-Hodgkin lymphoma	DTP	Public health region vaccination rates; 4 doses	Ever / Never	115 / -		0.88	(0.58 to 1.32)

Table continues

Supplementary Table 2. Continued

Reference	Age Range	Exposure	Outcome	Comments	Cancer Site	Vaccine	Subsample	Model (Estimate / Ref)	No. of cases (Estimate / Ref)	No. of Controls or Expected (Estimate / Ref)	OR	95%CI
					Non-Hodgkin lymphoma	DTP	County-level vaccination rates; 4 doses	Ever / Never	71 / -		2.34	(0.93 to 5.90)
					Non-Hodgkin lymphoma	DTP, Polio (IPV) & MMR	Public health region vaccination rates; 4 doses DTP, 3 doses Polio (IPV), 1 dose MMR	Ever / Never			0.98	(0.59 to 1.64)
					Non-Hodgkin lymphoma	DTP, Polio (IPV) & MMR	County-level vaccination rates; 4 doses DTP, 3 doses Polio (IPV), 1 dose MMR	Ever / Never			1.13	(0.71 to 1.82)
					Non-Hodgkin lymphoma	DTP, Polio (IPV), MMR & Hib	Public health region vaccination rates; 4 doses DTP, 3 doses Polio (IPV), 1 dose MMR, 3 doses Hib	Ever / Never			1.18	(0.70 to 1.98)
					Non-Hodgkin lymphoma	DTP, Polio (IPV), MMR & Hib	County-level vaccination rates; 4 doses DTP, 3 doses Polio (IPV), 1 dose MMR, 3 doses Hib	Ever / Never			1.22	(0.40 to 3.69)
					Non-Hodgkin lymphoma	DTP, Polio (IPV), MMR, Hib, Hep & Chickenpox	County-level vaccination rates; 4 doses DTP, 3 doses Polio (IPV), 1 dose MMR, 3 doses Hib, 3 doses Hep, 1 dose Chickenpox	Ever / Never			0.84	(0.51 to 1.38)
					Non-Hodgkin lymphoma	Hep B	County-level vaccination rates; 3 doses	Ever / Never			0.77	(0.32 to 1.81)
					Non-Hodgkin lymphoma	Hib	Public health region vaccination rates; 3 doses	Ever / Never			0.65	(0.26 to 1.59)
					Non-Hodgkin lymphoma	Hib	County-level vaccination rates; 3 doses	Ever / Never			0.98	(0.59 to 1.64)
					Non-Hodgkin lymphoma	MMR	Public health region vaccination rates; 1 dose	Ever / Never			0.99	(0.63 to 1.55)
					Non-Hodgkin lymphoma	MMR	County-level vaccination rates; 1 dose	Ever / Never			2.81	(1.27 to 6.22)
					Non-Hodgkin lymphoma	Polio (IPV)	Public health region vaccination rates; 3 doses	Ever / Never			1.01	(0.59 to 1.74)
					Non-Hodgkin lymphoma	Polio (IPV)	County-level vaccination rates; 3 doses	Ever / Never			0.73	(0.31 to 1.72)

Abbreviations: Adj, Adjustment; AL, Acute leukemia; ALL Acute lymphoblastic leukemia; AML, Acute myeloid leukemia; BCG, Bacillus Calmette–Guérin (vole bacillus; tuberculosis); BCP, B-Cell precursor; cALL, Common acute lymphoblastic leukemia; CNS, Central nervous system; DT, Diphtheria-Tetanus; DTP, Diphtheria-Tetanus-Pertussis/Whooping cough; DTPolio, Diphtheria-Tetanus-Poliomyelitis; Exc, Exclusion for meta-analysis; Hep, Hepatitis; Hib, Haemophilus influenzae type b; IPV, Inactivated poliomyelitis vaccine; Mat, Matching; MMR, Measles-Mumps-Rubella; MRC, Medical Research Council; SE, standard error.

[a] Calculation of crude ORs.

[b] Calculation of crude ORs taking individual matching into account.

[c] Study included in meta-analysis.

[d] Estimate included in meta-analysis.

[e] Inverted reference category.

Table continues
