# Supplementary material for: Vaccination and the Risk of Childhood Cancer—A Systematic Review and Meta-Analysis
Source: Front Oncol. 2021 Jan 22;10:610843. doi: 10.3389/fonc.2020.610843 (PMC7862764; doi:10.3389/fonc.2020.610843)
Supplement: Supplementary file 3 [file DataSheet_3.pdf]

Supplementary Table 3A. Newcastle-Ottawa quality score of included studies in the review and meta-analysis on vaccination and risk of childhood cancer.

Reference	Inclusion in meta-analyzes (0 = No, 1 = Yes)	NOS cohort: sum of quality score 0-9 points (P)	NOS case-control: sum of quality score 0-9 P	Cohort																								
				Selection													Compa-rability		Outcome									
				Representative-ness of exposed cohort				Selection of non-exposed cohort			Ascertainment				No out-come present at start				Assessment				Follow up long enough		Adequacy of follow up			
				Truely representative 1 P	Somewhat representative 1 P	Selected group (nurses, volunteers, trial…)	No description	Same community 1 P	Different source	No description	Secure records 1 P	Structured interview 1 P	Self-report	No description	Yes 1 P	No	Study controls for other vaccines 1 P	Study controls for any immunological factors 1 P	Independent blind assessment 1 P	Record linkage 1 P	Self-report	No description	Yes (5+ years) 1 P	No	Complete follow up 1 P	Small number lost, unlikely to introduce bias 1 P	High number lost, no description of those lost	No description
Innis, 1968[a,d]	1		**3**																									
Davignon, 1970[a,d]	1	**4**		1				1			1					0				1				0				0
MRC, 1972[a,d]	1	**5**				0		1			1					0				1			1		1			
Heinonen, 1973[a,d]	1	**7**			1			1			1				1					1			1			1		
Mathé, 1974[a,d]	1		**3**																									
Comstock, 1975[a,d]	1	**5**				0		1			1				1					1			1					0
Salonen, 1975[d]	1		**6**																									
Crispen, 1976[a,d]	1	**5**		1				1			1					0				1			1					0
Salonen, 1976[a,d]	1		**5**																									
Andersen, 1978[a,d]	1		**3**																									
Snider, 1978[a,d]	1	**4**				0		1			1					0				1			1					0
Farwell, 1979[a,d]	1		**3**																									
Neumann, 1980[a,d]	1		**3**																									
Kendrick, 1981[a,d]	1	**3**				0		1			1					0						0	1				0	
Sutherland, 1982[a,d]	1	**5**				0		1			1					0				1			1		1			
Steensel-Moll, 1985[d]	1		**5**																									
Kneale, 1986[c,d]	1		**4**																									
McKinney, 1987[d]	1		**4**																									
Nishi, 1989[d]	1		**2**																									
Petridou, 1997[b,d]	1		**4**																									
Kaatsch, 1998[d]	1		**3**																									
Dockerty, 1999[d]	1		**5**																									
Groves, 1999[d]	1		**4**																									
Schüz, 1999[d]	1		**4**																									
Auvinen, 2000[d]	1	**6**				0		1			1				1		1		1				1					0
Von Kries, 2000[d]	1		**5**																									
Krone, 2003[d]	1		**6**																									
Frentzel-Beyme, 2004[d]	1		**5**																									
Ma, 2005[d]	1		**5**																									
Mallol-Mesnard, 2007[b,d]	1		**6**																									
MacArthur, 2008[d]	1		**5**																									
Villumsen, 2009[d]	1	**8**		1				1			1				1			1		1			1		1			
Pagaoa, 2011[d]	1		**5**																									
Soegaard, 2017[d]	1	**8**		1				1							1		1	1		1			1					0
Figueroa, 2019[d]	1		**6**																									
Fraumeni, 1963	0		**3**																									
Innis, 1965	0		**2**																									
Stewart, 1965[a]	0		**2**																									
Waaler, 1970	0		**3**																									
Berkeley, 1971	0		**3**																									
Comstock, 1971[a]	0	**4**				0		1			1					0				1			1				0	
Hems, 1971	0		**3**																									
Kinlen, 1971	0		**3**																									
Rosenthal, 1972[a]	0	**7**			1			1			1				1					1			1			1		
Hofman, 1977	0		**3**																									
Ambrosch, 1978	0		**3**																									
Sinniah, 1978	0		**4**																									
Skegg, 1978	0		**4**																									
Nilsson, 1979	0		**3**																									
Gruffermann, 1982	0		**3**																									
Farwell, 1984	0		**3**																									
Hartley, 1988	0		**2**																									
Gilman, 1989	0		**5**																									
Buckley, 1994	0		**5**																									
Shu, 1995	0		**6**																									
Kaatsch, 1996	0		**4**																									
Bhatia, 1997	0		**5**																									
Daniels, 2008	0		**5**																									
Qu, 2014[b]	0	**7**		1				1			1				1					1			1			1		
Sankaran, 2016	0		**4**																									

Abbreviations: MRC, Medical Research Council; P, points. [a] Calculation of crude ORs. [b] Partly calculation of crude ORs not included in meta-analysis. [c] Calculation of crude ORs taking individual matching into account. [d] Study included in meta-analysis.

Table continues

Supplementary Table 3A. Continued

Case-Control																						Comments
Selection										Compa-rability		Exposure										
Adequate case definition			Case represen-tative-ness		Control selection			Control definition				Ascertainment					Same ascertain-ment method		Non-response rate			
Yes (independent validation) 1 P	Yes (record linkage/self-reports)	No description	Consecutive or obviously representative 1 P	Potential for selection bias / not stated	Community controls 1 P	Hospital Controls	No description / aggregated data	No history of disease (endpoint) 1 P	No description of source	Study controls for other vaccines 1 P	Study controls for any immunological factors 1 P	Secure records 1 P	Structured interview with blind status 1 P	Interview without blind status	Self-report / medical record	No description /aggregated data	Yes 1 P	No	Same rate for both groups (<10%) 1 P	Non respondents described	Different rate / no designation	
	0		1			0		1							0		1				0	
	0		1		1				0						0		1				0	
1			1		1				0		1				0		1		1			
1			1		1				0		[1]				0		1		1			
	0		1		1				0					0			1				0	
	0		1		1				0						0		1				0	
	0		1		1				0						0		1				0	
																						Calculation of lost-to-follow-up from overall number
1			1		1				0						0		1		1			
	0		1		1				0		1				0		1				0	
1			1			0		1						0			1				0	
	0		1			0			0						0		1				0	
1			1			0			0						0		1		1			
	0		1		1				0					0			1				0	Response rate in cases & controls differs
1			1		1				0					0			1		1			
		0	1		1				0		1				0		1				0	Response rate in cases & controls differs
	0		1		1			1						0			1				0	Response rate in cases & controls differs
	0		1		1			1						0			1		1			
1			1		1			1						0			1		1			
1			1		1			1						0			1				0	
	0		1		1			1			1				0		1				0	Response rate in cases & controls differs
1			1		1			1			1				0		1				0	Response rate in cases & controls differs
1			1		1				0		1			0			1				0	Response rate in cases & controls differs
	0		1		1			1			1					0	1				0	
1			1		1			1			1			0			1			0		
		0	1		1				0							0	1				0	
	0		1			0			0						0		1				0	
	0		1				0		0							0	1				0	
		0	1		1				0							0	1				0	
	0		1		1				0							0	1				0	
		0	1		1				0							0	1				0	
	0		1		1				0							0	1				0	
		0	1		1				0							0	1				0	
		0	1		1				0							0	1				0	
1			1		1				0							0	1				0	
	0		1		1				0	1						0	1				0	
	0		1		1				0							0	1				0	
	0		1		1				0					0			1			0		
	0		1		1				0						0		1				0	
	0		1			0			0						0		1				0	
	0		1		1			1			1				0		1				0	
1			1		1			1							0		1				0	
1			1		1				0		1				0		1		1			
	0		1		1			1						0			1				0	Response rate in cases & controls differs
	0		1		1			1			1				0		1				0	
1			1		1				0						0		1		1			
	0		1		1				0						0		1		1			

Supplementary Table 3B. Detailed quality score of included studies in the review and meta-analysis on vaccination and risk of childhood cancer.

			Study design 0-6 P															Sample size 0-6 P							Outcome 0-6 P							Exposure 0-6 P							Confounder Control 0-6 P			
			Intervention studies					Observational studies																																		
Reference	Inclusion in meta-analyzes (0 = No, 1 = Yes)	**Sum of quality score -6 - 45 points (P)**	Randomized, double-blinded 6 P	Randomized, single-blinded 5 P	Randomized, unblinded 4 P	Not randomized 2 P	Uncontrolled experiment 1 P	Prospective cohort 4 P	Retrospective cohort 4 P	Case-cohort 4 P	Register 4 P	Nested case-control 4 P	Population-based case-control 3 P	Case-control 2 P	Hospital-based case-control 2 P	Cross-sectional 1 P	Ecological 0 P	>1 000 000 6 P	>100 000 - < 1 000 000 5 P	>10 000 - < 100 000 4 P	>5 000 - < 10 000 3 P	>1 000 - < 5 000 2 P	>500 - < 1 000 1 P	1 - 500 0 P / no information	Trial data / labor verification 6 P	Accounting data 5 P	Registry data 4 P	Medical documentation 3 P	Record (death certificate) 2 P	Self-report 1 P	Aggregated data / no information 0 P	Trial data 6 P	Accounting data 5 P	Registry data 4 P	Medical documentation 3 P	Personal documentation (vaccination card) 2 P	Self-report 1 P	Aggregated data / no information 0 P	Controlling for similar exposure 3 P (other vaccine)	Control for other immunological factors 2 P	Control for other factors 1 P	No controlling 0 P
**Number of studies in the review (N=60)**									**13**	**1**			**28**	**1**	**6**		**11**	**2**	**6**	**11**		**15**	**9**	**17**	**15**	**1**	**24**	**9**	**4**		**7**	**8**	**1**	**1**	**8**	**7**	**23**	**12**	**3**	**12**	**31**	**18**
**Percent of studies in the review (%)**									**22**	**2**			**47**	**2**	**10**		**18**	**3**	**10**	**18**		**25**	**15**	**28**	**25**	**2**	**40**	**15**	**7**		**12**	**13**	**2**	**2**	**13**	**12**	**38**	**20**	**5**	**20**	**52**	**30**
**Number of studies in meta-analysis (N=35)**									**10**	**1**			**19**		**4**		**1**	**1**	**4**	**7**		**10**	**5**	**8**	**11**		**15**	**4**	**3**		**2**	**6**	**1**	**1**	**6**	**7**	**13**	**1**	**2**	**9**	**20**	**7**
**Percent of studies in meta-analysis (%)**									**29**	**3**			**54**		**11**		**3**	**3**	**11**	**20**		**29**	**14**	**23**	**31**		**43**	**11**	**9**		**6**	**17**	**3**	**3**	**17**	**20**	**37**	**3**	**6**	**26**	**57**	**20**
Innis, 1968[a,d]	1	**15.8**													2							2						3							3						[1]	
Davignon, 1970[a,d]	1	**23.9**							4										5								4							4								0
MRC, 1972[a,d]	1	**21.4**							4											4							4					6										0
Heinonen, 1973[a,d]	1	**19.6**							4											4									2						3						[1]	
Mathé, 1974[a,d]	1	**11.2**											3											0				3								2					[1]	
Comstock, 1975[a,d]	1	**25.1**							4										5								4					6										0
Salonen, 1975[d]	1	**25.8**											3									2			6										3					2	1	
Crispen, 1976[a,d]	1	**21.5**							4										5										2						3							0
Salonen, 1976[a,d]	1	**22.4**											3									2			6										3					[2]	[1]	
Andersen, 1978[a,d]	1	**19.3**											3											0			4								3						[1]	
Snider, 1978[a,d]	1	**24.5**							4											4							4					6										0
Farwell, 1979[a,d]	1	**15.8**											3											0			4										1				[1]	
Neumann, 1980[a,d]	1	**13.8**											3											0					2								1				[1]	
Kendrick, 1981[a,d]	1	**21.2**							4											4											0	6										0
Sutherland, 1982[a,d]	1	**23.8**							4											4							4					6										0
Steensel-Moll, 1985[d]	1	**21.8**											3									2			6												1				1	
Kneale, 1986[c,d]	1	**19.6**											3							4								3									1			2	1	
McKinney, 1987[d]	1	**22.6**													2								1		6												1				1	
Nishi, 1989[d]	1	**15.8**													2									0				3									1				1	
Petridou, 1997[b,d]	1	**19.0**													2									0	6												1				1	
Kaatsch, 1998[d]	1	**21.6**											3									2					4										1				1	
Dockerty, 1999[d]	1	**24.2**											3											0	6											2					1	
Groves, 1999[d]	1	**18.0**											3										1								0					2				2	1	
Schüz, 1999[d]	1	**22.2**											3									2					4										1				1	
Auvinen, 2000[d]	1	**35.4**							4										5						6							6							3			
Von Kries, 2000[d]	1	**22.2**											3									2					4										1				1	
Krone, 2003[d]	1	**24.4**											3									2			6												1				1	
Frentzel-Beyme, 2004[d]	1	**21.0**											3											0	6												1				1	
Ma, 2005[d]	1	**23.2**											3										1				4									2				2	1	
Mallol-Mesnard, 2007[b,d]	1	**27.8**											3									2			6											2				2	1	
MacArthur, 2008[d]	1	**26.6**											3										1		6											2				2	1	
Villumsen, 2009[d]	1	**27.6**								4												2					4									2				2	1	
Pagaoa, 2011[d]	1	**13.7**															0			4							4											0		2	1	
Soegaard, 2017[d]	1	**37.0**							4									6									4						5						3	2	1	
Figueroa, 2019[d]	1	**21.6**											3										1				4										1				1	
Fraumeni, 1963	0	**6.9.**															0	6													0							0				0
Innis, 1965	0	**12.2**													2									0				3							3							0
Stewart, 1965[a]	0	**7.7**												2								2						3										0				0
Waaler, 1970	0	**-3.4**															0							0							0							0				0
Berkeley, 1971	0	**4.2**															0							0			4											0				0
Comstock, 1971[a]	0	**20.8**							4											4								3				6									[1]	
Hems, 1971	0	**-2.2**															0							0							0							0				0
Kinlen, 1971	0	**4.9**															0							0			4											0				0
Rosenthal, 1972[a]	0	**23.5**							4										5								4								3						[1]	
Hofman, 1977	0	**-2.6**															0							0							0							0				0
Ambrosch, 1978	0	**-0.6**															0							0							0							0				0
Sinniah, 1978	0	**11.5**															0			4					6													0				0
Skegg, 1978	0	**10.0**															0							0			4											0	3			
Nilsson, 1979	0	**6.2**															0		5								4											0				0
Gruffermann, 1982	0	**17.8**											3											0				3									1				1	
Farwell, 1984	0	**18.6**											3										1				4										1				1	
Hartley, 1988	0	**18.2**													2							2					4										1				1	
Gilman, 1989	0	**21.2**											3							4								3									1			2	1	
Buckley, 1994	0	**21.0**											3									2			6												1				1	
Shu, 1995	0	**22.4**											3										1		6												1			2	1	
Kaatsch, 1996	0	**21.0**											3										1				4										1				1	
Bhatia, 1997	0	**20.0**											3									2							2								1			2	1	
Daniels, 2008	0	**22.2**											3									2			6												1				1	
Qu, 2014[b]	0	**32.4**							4											4							4					6									1	
Sankaran, 2016	0	**21.2**											3										1			5											1				1	

Abbreviations: MRC, Medical Research Council; P, points. [a] Calculation of crude ORs. [b] Partly calculation of crude ORs not included in meta-analysis. [c] Calculation of crude ORs taking individual matching into account. [d] Study included in meta-analysis. [e] as indicated by published studies.

Table continues

Supplementary Table 3B. Continued

Statistics 0-6 P					Methods 0-6 P							Reported basic characteristics 0-3 P																
Statistic appropriate (CI and/or P-value) 2 P	Power calculations indicated 1 P	Statistical methods applied 1 P	Statistical methods indicated 1 P	Primary objective defined 1 P	Good selection of cases (incidence) 1 P	Good selection of controls (e.g. no cancer) 1 P	Generalization of results (extern validity) 0.5 P	Response 60+% 1 P	Follow-up of 5+ years / loss to follow up < 50 % 1P	Blinded evaluator / trained interviewer 1 P	Reference group randomized 0.5 P[e]	Information on study population 0.2 P	Sex 0.2 P	Age 0.2 P	Age at exposure (vaccination) 0.2 P	Age at diagnosis (cancer) 0.2 P	Exposure description (vaccination) 0.2 P	Outcome description (cancer Site) 0.2 P	In- and exclusion criteria indicated 0.2 P	Recruitment described 0.2 P	Numbers described 0.2 P	Reference group available 0.2 P	Information on study center 0.2 P	Multi-center study 0.2 P	Several times of investigation 0.2 P	Discussion of bias and limitations 0.2 P	Limitation, confounder, bias not noted up to -6 P	Comments
**45**	**4**	**53**	**43**	**56**	**20**	**41**	**24**	**23**	**23**	**11**	**22**	**40**	**55**	**29**	**16**	**44**	**27**	**55**	**47**	**51**	**54**	**53**	**29**	**13**	**6**	**50**	**104**	
**75**	**7**	**88**	**72**	**93**	**33**	**68**	**39**	**38**	**38**	**18**	**37**	**67**	**92**	**48**	**27**	**73**	**45**	**92**	**78**	**85**	**90**	**88**	**48**	**22**	**10**	**83**	**68**	
**30**	**1**	**32**	**27**	**34**	**15**	**24**	**17**	**19**	**12**	**9**	**15**	**27**	**34**	**19**	**9**	**26**	**17**	**33**	**33**	**33**	**35**	**34**	**19**	**8**	**3**	**31**	**31**	
**86**	**3**	**91**	**77**	**97**	**43**	**69**	**47**	**54**	**34**	**26**	**41**	**77**	**97**	**54**	**26**	**74**	**49**	**94**	**94**	**94**	**100**	**97**	**54**	**23**	**9**	**89**	**60**	
2		1				1	0.5				0.5		0.2				0.2		0.2	0.2	0.2	0.2	0.2	0.2		0.2	-1	No latency
2		1	1	1			0.5	1					0.2					0.2		0.2	0.2	0.2	0.2			0.2	-1	No latency
				1			0.5		1		0.5		0.2						0.2	0.2	0.2	0.2			0.2	0.2	-1	No latency
2				1			0.5		1		0.5	0.2	0.2		0.2	0.2	0.2	0.2	0.2	0.2	0.2	0.2		0.2	0.2	0.2	-1	No latency
				1		1	0.5				0.5		0.2					0.2	0.2		0.2	0.2	0.2				-1	No latency
		1		1		1	0.5		1			0.2	0.2			0.2	0.2	0.2	0.2		0.2	0.2						
2		1	1	1		1	0.5	1			0.5	0.2	0.2			0.2		0.2		0.2	0.2	0.2	0.2			0.2	-1	No latency
2		1	1	1			0.5		1			0.2	0.2	0.2		0.2	0.2	0.2	0.2	0.2	0.2		0.2				-1	No latency
2		1	1	1			0.5	1			0.5	0.2	0.2	0.2	0.2		0.2	0.2	0.2	0.2	0.2	0.2	0.2			0.2	-1	No latency
2		1	1	1		1	0.5			1		0.2	0.2					0.2	0.2	0.2	0.2	0.2	0.2			0.2		
		1		1	1		0.5		1			0.2	0.2			0.2	0.2	0.2	0.2	0.2	0.2	0.2				0.2		
2		1		1		1	0.5		1		0.5		0.2			0.2	0.2	0.2	0.2	0.2	0.2	0.2				0.2	-1	No latency
2		1	1	1			0.5		1		0.5		0.2	0.2		0.2		0.2	0.2	0.2	0.2	0.2				0.2	-1	No latency
2		1	1	1					1			0.2	0.2	0.2	0.2	0.2		0.2	0.2	0.2	0.2	0.2				0.2	-1	No latency
		1	1	1			0.5		1		0.5		0.2			0.2		0.2	0.2	0.2	0.2	0.2			0.2	0.2	-1	No latency
2		1	1	1		1	0.5	1			0.5	0.2				0.2	0.2	0.2	0.2	0.2	0.2	0.2				0.2	-1	No latency
2		1		1			0.5				0.5		0.2		0.2		0.2	0.2	0.2	0.2	0.2	0.2					-1	No latency
2		1	1	1		1	0.5	1		1	0.5	0.2	0.2	0.2	0.2	0.2		0.2	0.2	0.2	0.2	0.2	0.2	0.2		0.2		
2		1	1	1		1	0.5				0.5	0.2	0.2	0.2		0.2	0.2	0.2	0.2	0.2	0.2	0.2	0.2	0.2		0.2	-1	No latency
2		1	1	1	1		0.5	1			0.5	0.2	0.2	0.2		0.2		0.2	0.2	0.2	0.2	0.2				0.2	-1	No latency
2		1	1	1	1	1	0.5	1		1	0.5	0.2	0.2					0.2	0.2	0.2	0.2	0.2				0.2	-1	No latency
2		1	1	1	1	1	0.5	1		1	0.5	0.2	0.2			0.2	0.2	0.2	0.2	0.2	0.2	0.2	0.2			0.2		
2		1	1	1		1	0.5	1			0.5	0.2	0.2	0.2		0.2		0.2	0.2	0.2	0.2	0.2				0.2	-1	No latency
2		1	1	1	1	1	0.5	1		1	0.5	0.2	0.2	0.2		0.2		0.2	0.2	0.2	0.2	0.2	0.2			0.2	-1	No latency
2		1	1	1	1	1	0.5	1	1		0.5	0.2	0.2	0.2	0.2	0.2	0.2	0.2	0.2	0.2	0.2	0.2				0.2	-1	No latency
2		1	1	1	1	1	0.5	1		1	0.5	0.2	0.2			0.2	0.2	0.2	0.2	0.2	0.2	0.2	0.2			0.2	-1	No latency
2		1	1	1	1	1	0.5	1		1	0.5	0.2	0.2	0.2		0.2		0.2	0.2	0.2	0.2	0.2	0.2	0.2		0.2	-1	No latency
2		1	1	1	1	1	0.5			1	0.5	0.2	0.2	0.2		0.2		0.2	0.2	0.2	0.2	0.2				0.2	-1	No latency; study included incident & prevalent cases
2		1	1	1	1	1	0.5	1			0.5	0.2	0.2	0.2		0.2		0.2	0.2	0.2	0.2	0.2	0.2			0.2	-1	No latency
2	1	1	1	1	1	1	0.5	1			0.5	0.2	0.2	0.2	0.2	0.2	0.2	0.2	0.2	0.2	0.2	0.2	0.2	0.2		0.2	-1	No latency
2		1	1	1	1	1	0.5	1		1	0.5	0.2	0.2	0.2	0.2	0.2		0.2	0.2	0.2	0.2	0.2	0.2	0.2		0.2	-1	No latency
2		1	1	1	1	1	0.5	1	1		0.5	0.2	0.2	0.2	0.2	0.2	0.2	0.2	0.2	0.2	0.2	0.2	0.2			0.2		
2		1	1	1		1					0.5	0.2	0.2	0.2		0.2		0.2	0.2	0.2	0.2	0.2	0.2			0.2	-6	No latency & ecological bias
2		1	1	1	1	1	0.5	1	1		0.5	0.2	0.2	0.2			0.2	0.2	0.2	0.2	0.2	0.2				0.2		
2		1	1	1	1	1	0.5	1			0.5	0.2	0.2	0.2		0.2	0.2	0.2	0.2	0.2	0.2	0.2	0.2	0.2		0.2		
		1	1	1		1			1		0.5		0.2		0.2	0.2		0.2			0.2	0.2				0.2	-6	No latency & ecological bias
2		1					0.5				0.5						0.2	0.2		0.2	0.2	0.2	0.2				-1	No latency
							0.5						0.2			0.2		0.2			0.2	0.2				0.2	-1	No latency
						1			1				0.2				0.2	0.2									-6	No latency & ecological bias
		1	1	1		1			1				0.2			0.2	0.2	0.2		0.2	0.2						-6	No latency & ecological bias
		1		1					1			0.2	0.2					0.2	0.2	0.2	0.2	0.2	0.2			0.2	-1	No latency
				1		1			1				0.2			0.2		0.2		0.2							-6	No latency & ecological bias
		1	1	1		1			1		0.5		0.2			0.2		0.2			0.2	0.2		0.2		0.2	-6	No latency & ecological bias
2		1	1	1			0.5		1				0.2		0.2	0.2	0.2	0.2		0.2	0.2	0.2	0.2			0.2	-1	No latency
		1		1									0.2			0.2	0.2	0.2			0.2	0.2				0.2	-6	No latency & ecological bias
		1	1	1		1			1				0.2					0.2									-6	No latency & ecological bias
2		1	1	1							0.5	0.2	0.2	0.2	0.2			0.2	0.2	0.2	0.2	0.2				0.2	-6	No latency & ecological bias
2		1	1	1		1			1				0.2			0.2		0.2	0.2	0.2		0.2	0.2	0.2	0.2	0.2	-6	No latency & ecological bias
				1					1				0.2				0.2		0.2	0.2	0.2	0.2					-6	No latency & ecological bias
2		1	1	1	1	1	0.5			1	0.5	0.2	0.2			0.2		0.2	0.2	0.2	0.2	0.2				0.2	-1	No latency
2		1		1	1	1	0.5				0.5	0.2	0.2	0.2	0.2	0.2	0.2	0.2	0.2	0.2	0.2	0.2	0.2			0.2	-1	No latency
2		1	1	1			0.5				0.5	0.2	0.2	0.2		0.2			0.2	0.2	0.2	0.2	0.2	0.2		0.2		
2		1	1	1		1	0.5				0.5	0.2			0.2		0.2				0.2	0.2				0.2	-1	No latency; different assessment = self-report supplied by general practitioner in 55% of cases & 59% of controls
2		1	1	1		1	0.5				0.5	0.2	0.2	0.2		0.2		0.2	0.2	0.2	0.2	0.2				0.2	-1	No latency
2		1	1	1		1	0.5				0.5	0.2	0.2	0.2		0.2		0.2	0.2	0.2	0.2	0.2	0.2	0.2		0.2	-1	No latency
2	1	1	1	1		1	0.5	1		1	0.5	0.2		0.2		0.2		0.2	0.2	0.2	0.2	0.2	0.2			0.2	-1	No latency
2	1	1		1		1	0.5	1			0.5	0.2	0.2	0.2		0.2		0.2	0.2	0.2			0.2		0.2	0.2	-1	No latency
2		1	1	1	1	1	0.5				0.5	0.2		0.2	0.2	0.2	0.2	0.2	0.2	0.2	0.2	0.2				0.2	-1	No latency
2	1	1	1	1	1	1	0.5	1	1		0.5	0.2	0.2	0.2	0.2	0.2	0.2	0.2	0.2	0.2				0.2	0.2	0.2		
2		1	1	1	1	1	0.5	1			0.5	0.2	0.2	0.2		0.2		0.2	0.2	0.2	0.2	0.2	0.2			0.2	-1	No latency
