# Supplementary material for: Vaccination and the Risk of Childhood Cancer—A Systematic Review and Meta-Analysis
Source: Front Oncol. 2021 Jan 22;10:610843. doi: 10.3389/fonc.2020.610843 (PMC7862764; doi:10.3389/fonc.2020.610843)
Supplement: Supplementary file 4 [file DataSheet_4.pdf]

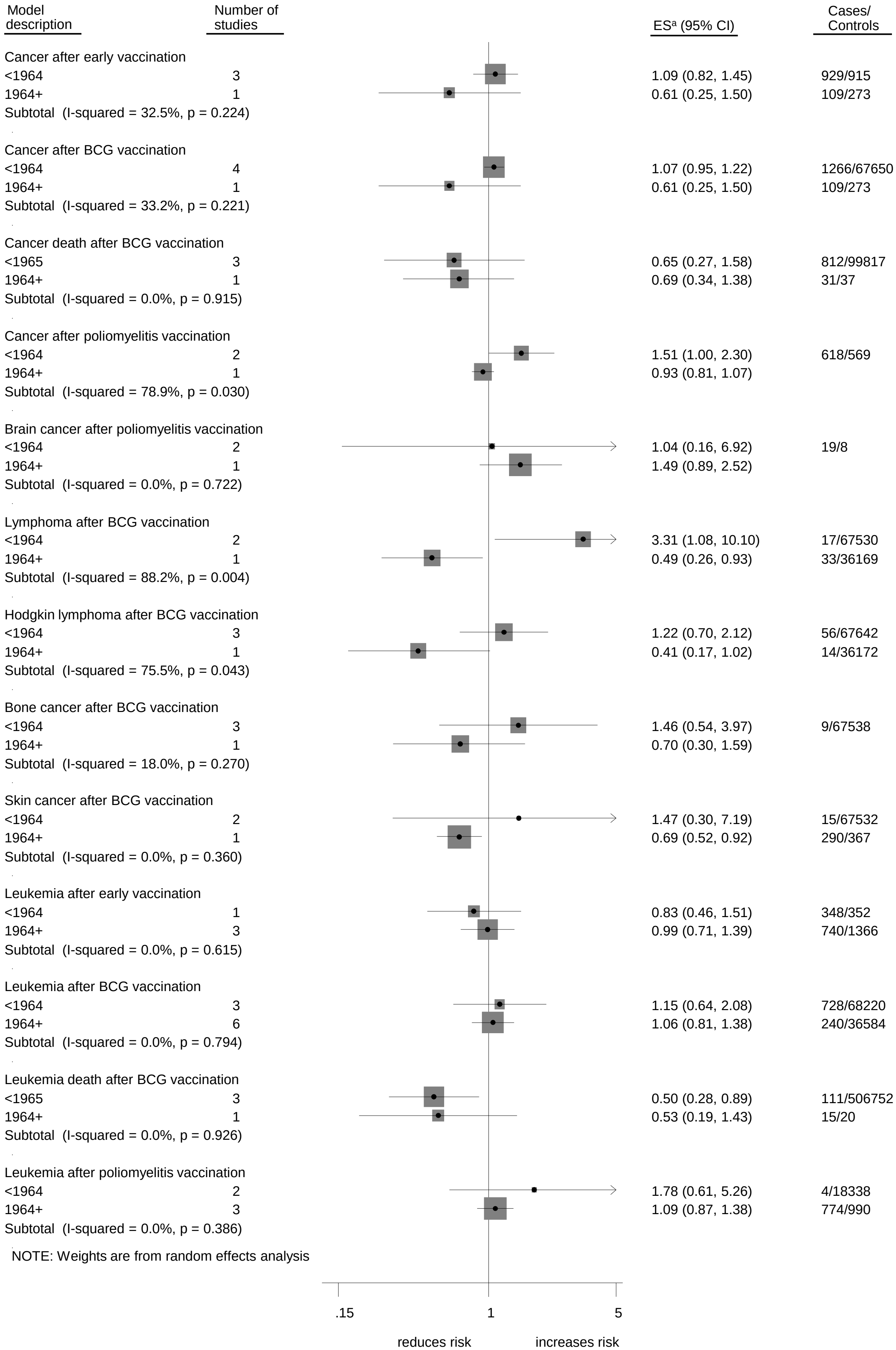

Supplementary Figure 1A. Vaccination and the risk of childhood cancer after stratification by year (study period).

Abbreviations: BCG, Bacillus Calmette-Guérin; OR, odds ratio; ES, estimate.

[a] ES includes single-study odds ratios or hazard ratios and summary odds ratios.

Model description	Number of studies	ES[a] (95% CI)	Cases/ Controls
Cancer after BCG vaccination			
case-control / ecological	3	1.08 (0.55, 2.15)	796/955
cohort / case-cohort	2	1.07 (0.94, 1.20)	579/66968
Subtotal (I-squared = 0.0%, p = 0.965)			
Cancer death after BCG vaccination			
case-control / ecological	2	0.81 (0.72, 0.93)	823/920
cohort / case-cohort	2	0.61 (0.10, 3.61)	20/98934
Subtotal (I-squared = 0.0%, p = 0.755)			
Hodgkin lymphoma after BCG vaccination			
case-control / ecological	1	0.98 (0.49, 1.97)	40/111
cohort / case-cohort	3	1.01 (0.34, 3.01)	30/103703
Subtotal (I-squared = 0.0%, p = 0.962)			
Bone cancer after BCG vaccination			
case-control / ecological	2	0.76 (0.35, 1.65)	
cohort / case-cohort	2	1.50 (0.49, 4.64)	9/67538
Subtotal (I-squared = 0.0%, p = 0.327)			
Skin cancer after BCG vaccination			
case-control / ecological	1	0.69 (0.52, 0.92)	290/367
cohort / case-cohort	2	1.47 (0.30, 7.19)	15/67532
Subtotal (I-squared = 0.0%, p = 0.360)			
Leukemia after BCG vaccination			
case-control / ecological	6	1.06 (0.81, 1.38)	922/1113
cohort / case-cohort	3	1.15 (0.64, 2.07)	46/103691
Subtotal (I-squared = 0.0%, p = 0.789)			
Leukemia death after BCG vaccination			
case-control / ecological	1	0.53 (0.19, 1.43)	15/20
cohort / case-cohort	3	0.50 (0.28, 0.89)	111/506752
Subtotal (I-squared = 0.0%, p = 0.926)			
Leukemia after poliomyelitis vaccination			
case-control / ecological	4	1.10 (0.88, 1.38)	774/990
cohort / case-cohort	1	1.78 (0.44, 7.10)	4/18338
Subtotal (I-squared = 0.0%, p = 0.504)			
Brain cancer after BCG vaccination			
case-control / ecological	1	1.10 (0.41, 2.90)	670/666
cohort / case-cohort	2	1.22 (0.50, 3.00)	16/67531
Subtotal (I-squared = 0.0%, p = 0.876)			
Kidney cancer after BCG vaccination			
case-control / ecological	1	1.10 (0.30, 4.60)	
cohort / case-cohort	2	2.09 (0.37, 11.77)	12/67535
Subtotal (I-squared = 0.0%, p = 0.568)			
ALL after number of vaccination			
case-control / ecological	4	0.60 (0.43, 0.85)	1033/2790
cohort / case-cohort	1	0.91 (0.28, 2.93)	3/
Subtotal (I-squared = 0.0%, p = 0.511)			
ALL after haemophilus influenzae type b vaccination			
case-control / ecological	3	0.73 (0.59, 0.88)	488/592
cohort / case-cohort	2	0.89 (0.63, 1.25)	464/1224914
Subtotal (I-squared = 0.0%, p = 0.324)			
ALL after diphtheria, tetanus, pertussis/poliomyelitis vaccination			
case-control / ecological	3	0.87 (0.72, 1.05)	1601/788
cohort / case-cohort	1	1.14 (0.42, 3.13)	486/
Subtotal (I-squared = 0.0%, p = 0.603)			
ALL after measles, mumps, rubella vaccination			
case-control / ecological	5	0.81 (0.58, 1.15)	1044/1230
cohort / case-cohort	1	1.01 (0.76, 1.34)	418/
Subtotal (I-squared = 0.0%, p = 0.341)			

NOTE: Weights are from random effects analysis

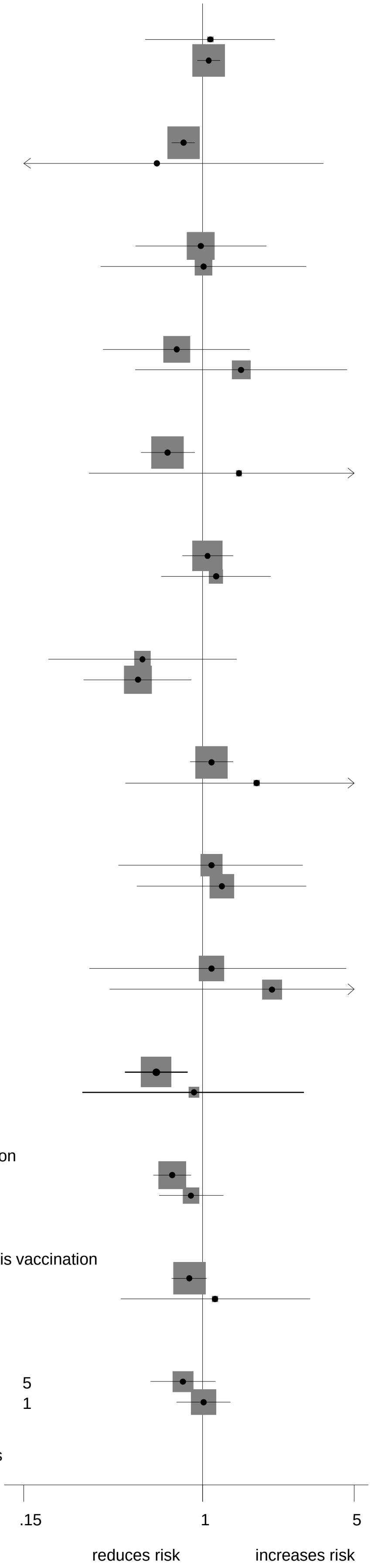

Supplementary Figure 1B. Vaccination and the risk of childhood cancer after stratification by study design.

Abbreviations: ALL, acute lymphoblastic leukemia; BCG, Bacillus Calmette-Guérin; OR, odds ratio; ES, estimate.

[a] ES includes single-study odds ratios or hazard ratios and summary odds ratios.

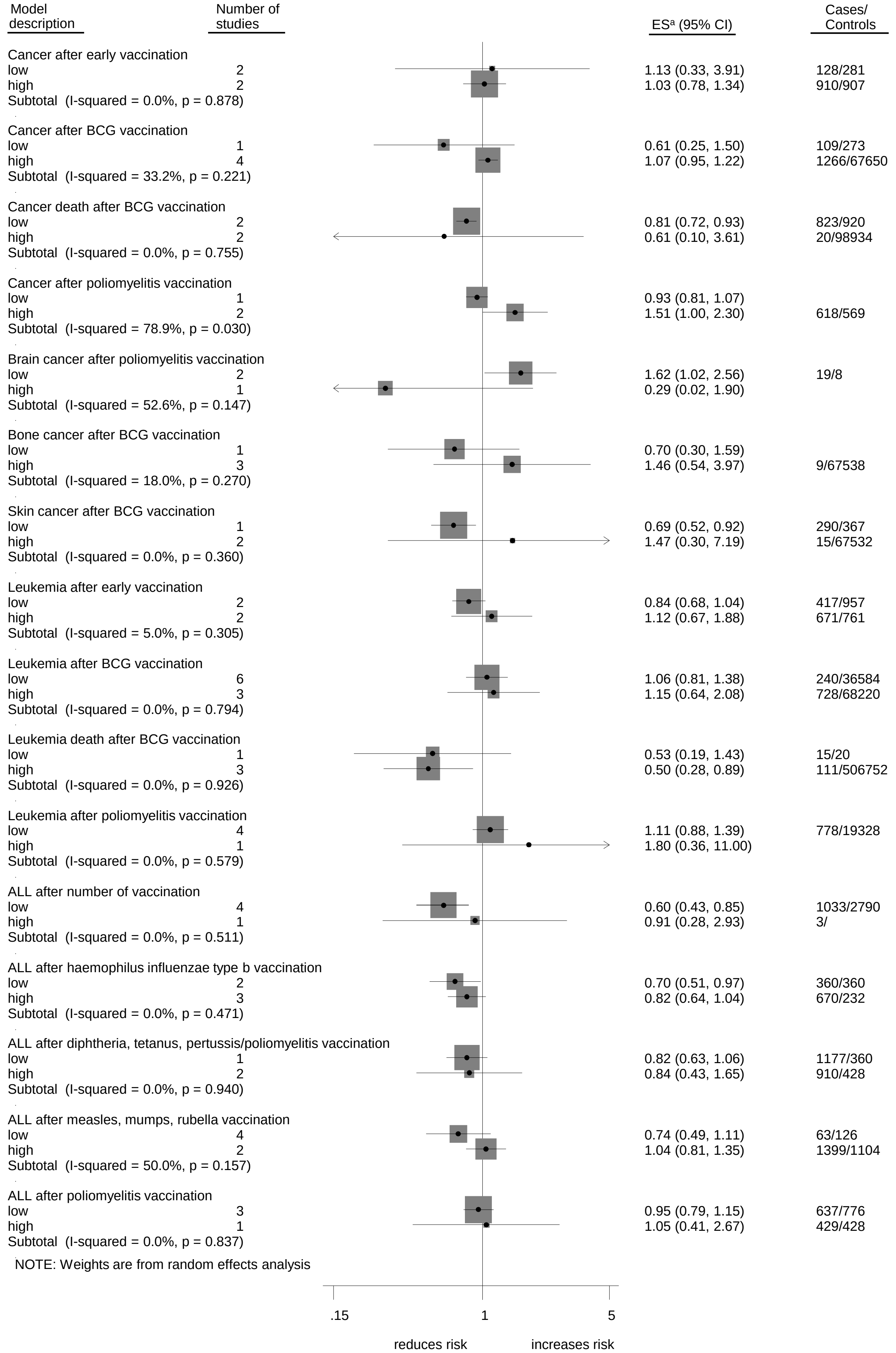

Supplementary Figure 1C. Vaccination and the risk of childhood cancer after stratification by exposure assessment (low: aggregated data, vaccination cards, self-report; high: trial data, accounting data, registry data, medical documentation).

Abbreviations: ALL, acute lymphoblastic leukemia; BCG, Bacillus Calmette-Guérin; OR, odds ratio; ES, estimate.

[a] ES includes single-study odds ratios or hazard ratios and summary odds ratios.

Model description	Number of studies	ES[a] (95% CI)	Cases/ Controls
Cancer after early vaccination			
no	1	1.05 (0.57, 1.92)	29/28
yes	3	1.06 (0.63, 1.78)	1009/1160
Subtotal (I-squared = 0.0%, p = 0.983)			
.			
Cancer after BCG vaccination [b]			
no	1	2.63 (0.93, 7.42)	13/5
yes	3	0.99 (0.79, 1.25)	933/51434
Subtotal (I-squared = 69.0%, p = 0.072)			
.			
Cancer after poliomyelitis vaccination			
no	1	1.69 (1.26, 2.26)	618/569
yes	2	0.93 (0.81, 1.07)	
Subtotal (I-squared = 92.4%, p = 0.000)			
.			
Skin cancer after BCG vaccination [b]			
no	1	0.69 (0.52, 0.92)	290/367
yes	1	0.72 (0.25, 2.08)	8/50626
Subtotal (I-squared = 0.0%, p = 0.939)			
.			
Leukemia after BCG vaccination [b]			
no	1	1.13 (0.75, 1.71)	76/677
yes	7	0.96 (0.70, 1.31)	872/87844
Subtotal (I-squared = 0.0%, p = 0.533)			
.			
Leukemia death after BCG vaccination			
no	2	0.41 (0.22, 0.78)	21/85370
yes	2	0.64 (0.23, 1.78)	105/421402
Subtotal (I-squared = 0.0%, p = 0.478)			
.			
Leukemia after poliomyelitis vaccination			
no	1	1.78 (0.44, 7.10)	4/18338
yes	4	1.10 (0.88, 1.38)	774/990
Subtotal (I-squared = 0.0%, p = 0.504)			
.			
ALL after measles, mumps, rubella vaccination [c]			
no	1	0.24 (0.10, 0.60)	63/126
yes	4	0.91 (0.78, 1.07)	1004/710
Subtotal (I-squared = 87.9%, p = 0.004)			
.			

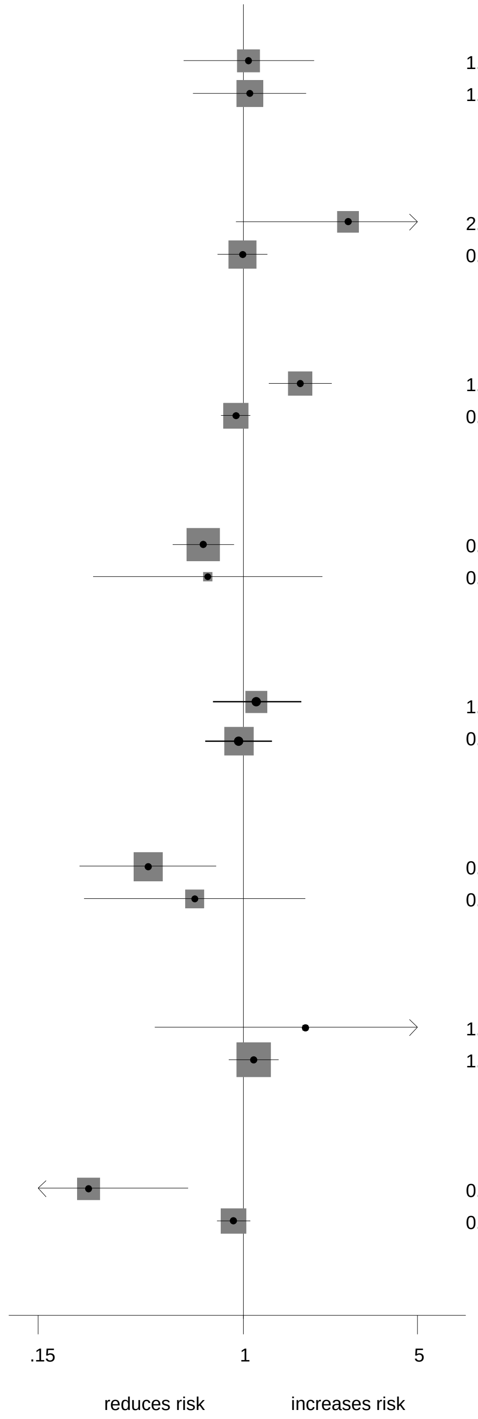

.

Supplementary Figure 1D. Vaccination and the risk of childhood cancer after stratification by assessment of outcome via trials, accounting data, or registries (yes) or other sources (no).

Abbreviations: ALL, acute lymphoblastic leukemia; BCG, Bacillus Calmette-Guérin; OR, odds ratio; ES, estimate.

[a] ES includes single-study odds ratios or hazard ratios and summary odds ratios.

ES from Kendrick [b] and Groves [c] were excluded for stratification, due to missing indication of outcome source.

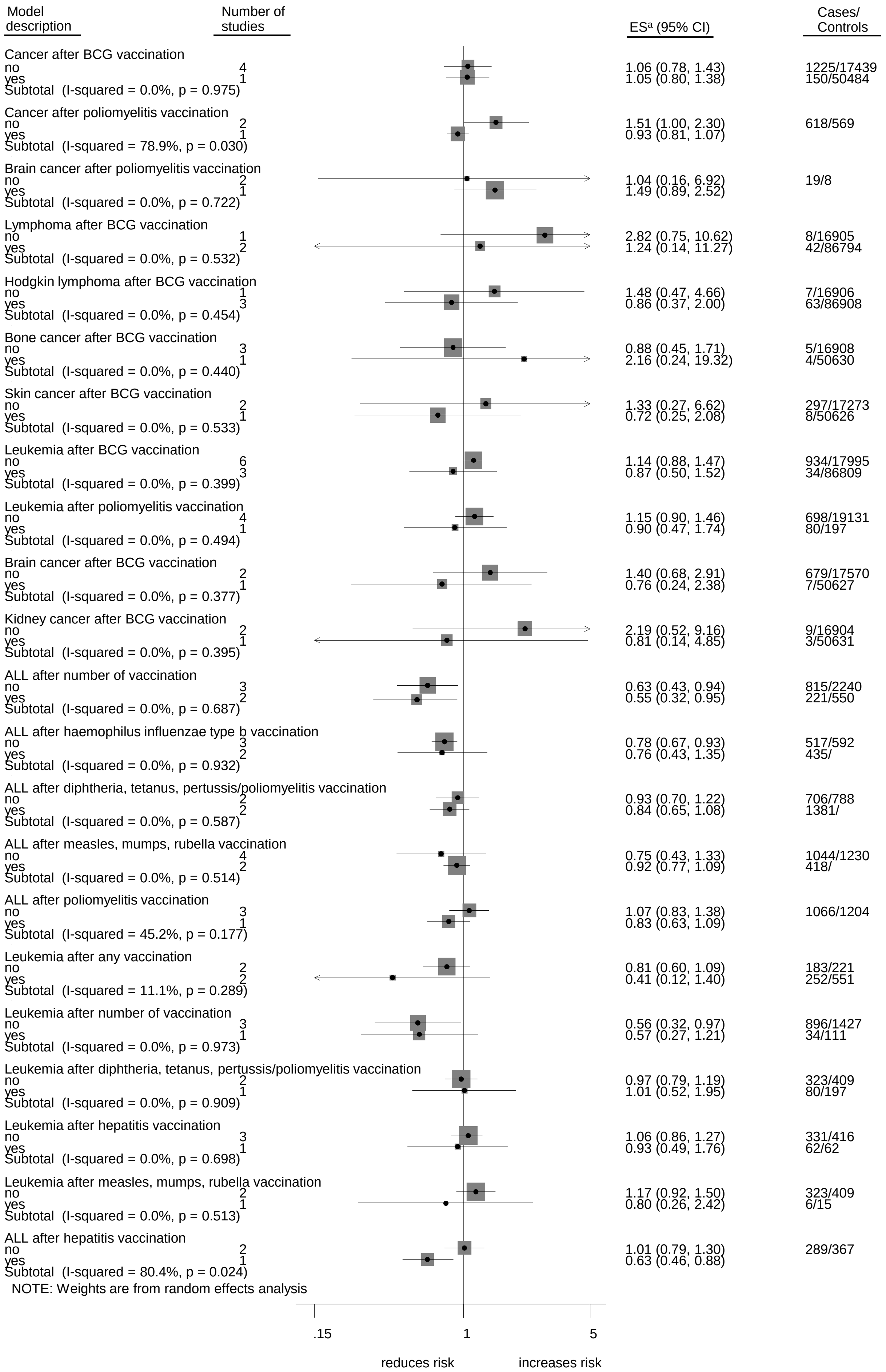

Supplementary Figure 1E. Vaccination and the risk of childhood cancer after stratification by latency period.

Abbreviations: ALL, acute lymphoblastic leukemia; BCG, Bacillus Calmette-Guérin; OR, odds ratio; ES, estimate.

[a] ES includes single-study odds ratios or hazard ratios and summary odds ratios.

Model description	Number of studies	ES[a] (95% CI)	Cases/ Controls
**Cancer after BCG vaccination**			
low	4	1.07 (0.87, 1.32)	701/67246
high	1	0.96 (0.58, 1.58)	674/677
Subtotal (I-squared = 0.0%, p = 0.694)			
**Cancer after poliomyelitis vaccination**			
low	2	1.24 (0.69, 2.22)	618/569
high	1	1.00 (0.43, 2.30)	
Subtotal (I-squared = 0.0%, p = 0.685)			
**Brain cancer after poliomyelitis vaccination**			
low	2	1.62 (1.02, 2.56)	19/8
high	1	0.29 (0.02, 1.90)	
Subtotal (I-squared = 52.6%, p = 0.147)			
**Lymphoma after BCG vaccination**			
low	2	3.31 (1.08, 10.10)	17/67530
high	1	0.49 (0.26, 0.93)	33/36169
Subtotal (I-squared = 88.2%, p = 0.004)			
**Hodgkin lymphoma after BCG vaccination**			
low	3	1.22 (0.70, 2.12)	56/67642
high	1	0.41 (0.17, 1.02)	14/36172
Subtotal (I-squared = 75.5%, p = 0.043)			
**Bone cancer after BCG vaccination**			
low	3	0.92 (0.47, 1.79)	9/67538
high	1	1.30 (0.17, 13.00)	
Subtotal (I-squared = 0.0%, p = 0.763)			
**Leukemia after early vaccination**			
low	3	1.07 (0.76, 1.51)	778/1034
high	1	0.83 (0.63, 1.00)	310/684
Subtotal (I-squared = 29.9%, p = 0.232)			
**Leukemia after BCG vaccination**			
low	6	1.15 (0.88, 1.49)	247/67904
high	3	0.87 (0.51, 1.48)	721/36900
Subtotal (I-squared = 0.0%, p = 0.359)			
**Leukemia after poliomyelitis vaccination**			
low	3	1.12 (0.88, 1.42)	407/18944
high	2	1.06 (0.46, 2.41)	371/384
Subtotal (I-squared = 0.0%, p = 0.895)			
**Brain cancer after BCG vaccination**			
low	1	1.90 (0.64, 5.67)	9/16904
high	2	0.94 (0.45, 1.98)	677/51293
Subtotal (I-squared = 7.5%, p = 0.299)			
**Kidney cancer after BCG vaccination**			
low	2	2.09 (0.37, 11.77)	12/67535
high	1	1.10 (0.30, 4.60)	
Subtotal (I-squared = 0.0%, p = 0.568)			
**ALL after number of vaccination**			
low	3	0.50 (0.39, 0.65)	755/2106
high	2	0.88 (0.66, 1.04)	278/684
Subtotal (I-squared = 90.3%, p = 0.001)			
**ALL after haemophilus influenzae type b vaccination**			
low	3	0.73 (0.59, 0.88)	488/592
high	2	0.89 (0.63, 1.25)	464/
Subtotal (I-squared = 0.0%, p = 0.324)			
**ALL after diphtheria, tetanus, pertussis/poliomyelitis vaccination**			
low	3	0.87 (0.72, 1.05)	1601/788
high	1	1.14 (0.42, 3.13)	486/
Subtotal (I-squared = 0.0%, p = 0.603)			
**ALL after measles, mumps, rubella vaccination**			
low	4	0.75 (0.43, 1.33)	740/880
high	2	0.92 (0.77, 1.09)	722/350
Subtotal (I-squared = 0.0%, p = 0.514)			
**ALL after poliomyelitis vaccination**			
low	3	0.95 (0.79, 1.15)	737/820
high	1	1.01 (0.37, 2.80)	329/384
Subtotal (I-squared = 0.0%, p = 0.910)			
**Leukemia after any vaccination**			
low	3	0.55 (0.27, 1.11)	267/565
high	1	0.81 (0.58, 1.13)	168/207
Subtotal (I-squared = 0.0%, p = 0.323)			
**Leukemia after number of vaccination**			
low	3	0.45 (0.33, 0.91)	620/854
high	1	0.83 (0.66, 1.05)	310/684
Subtotal (I-squared = 78.4%, p = 0.031)			
**Leukemia after hepatitis vaccination**			
low	3	1.05 (0.88, 1.25)	385/560
high	1	1.09 (0.34, 3.52)	8/7
Subtotal (I-squared = 0.0%, p = 0.951)			
**ALL after hepatitis vaccination**			
low	2	0.81 (0.51, 1.28)	282/360
high	1	1.08 (0.32, 3.68)	7/7
Subtotal (I-squared = 0.0%, p = 0.660)			

NOTE: Weights are from random effects analysis

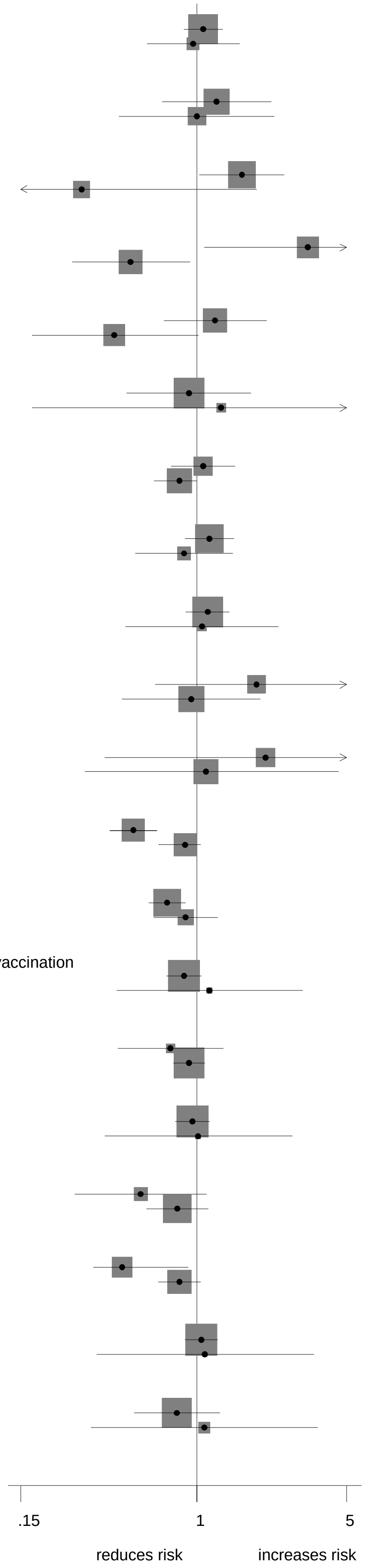

Supplementary Figure 1F. Vaccination and the risk of childhood cancer after stratification by study quality (low: below 24.7 points (fourth quintile of quality score of included studies); high: equal or above 24.7 points (fourth quintile of quality score of included studies)).

Abbreviations: ALL, acute lymphoblastic leukemia; OR, odds ratio; ES, estimate.

[a] ES includes single-study odds ratios or hazard ratios and summary odds ratios.

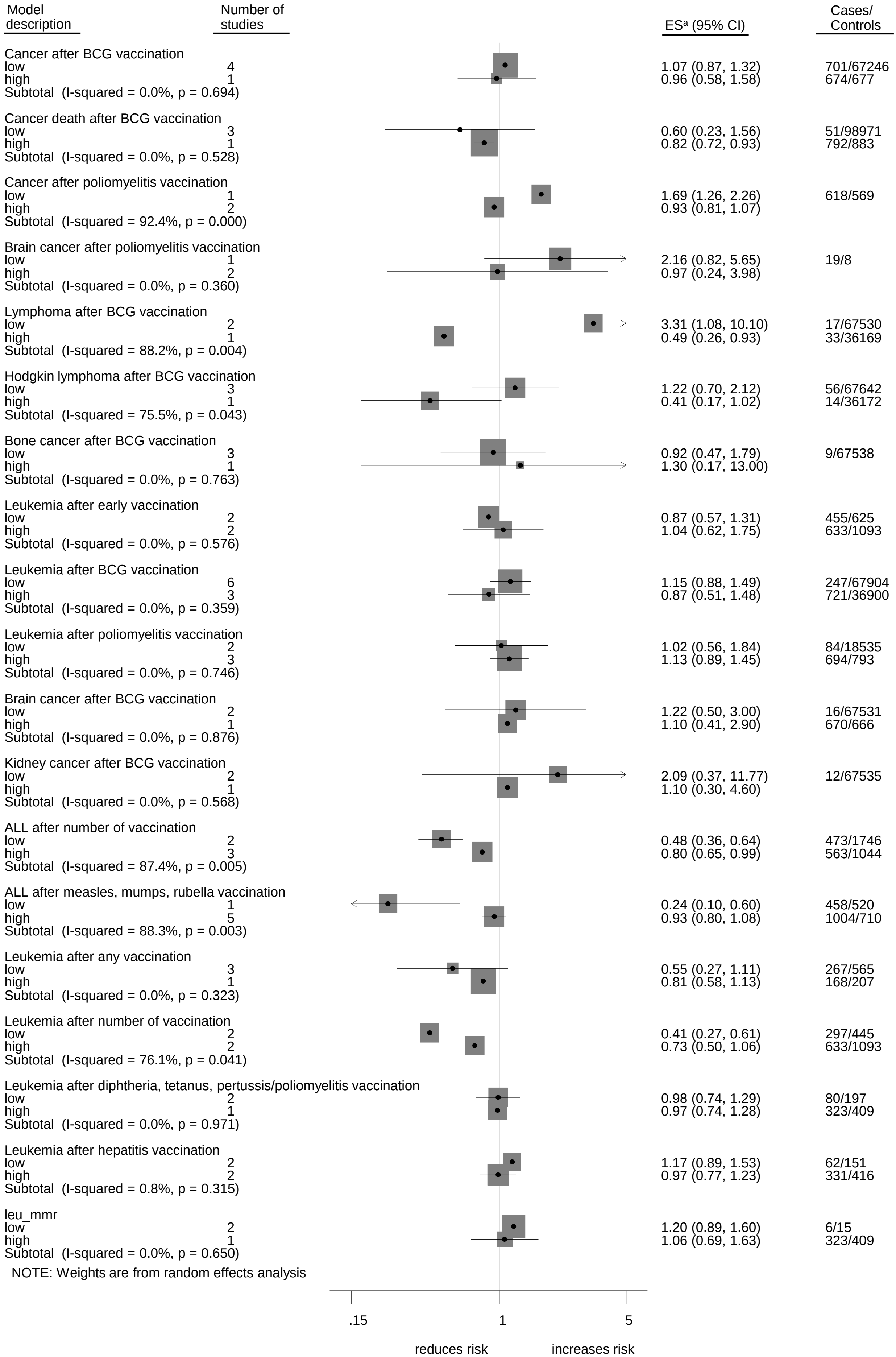

Supplementary Figure 1G. Vaccination and the risk of childhood cancer after stratification by controlling of confounders (low: no controlling, control for basic factors; high: control for other exposures in the pathway, control for similar exposure).

Abbreviations: ALL, acute lymphoblastic leukemia; BCG, Bacillus Calmette-Guérin; OR, odds ratio; ES, estimate.

[a] ES includes single-study odds ratios or hazard ratios and summary odds ratios.

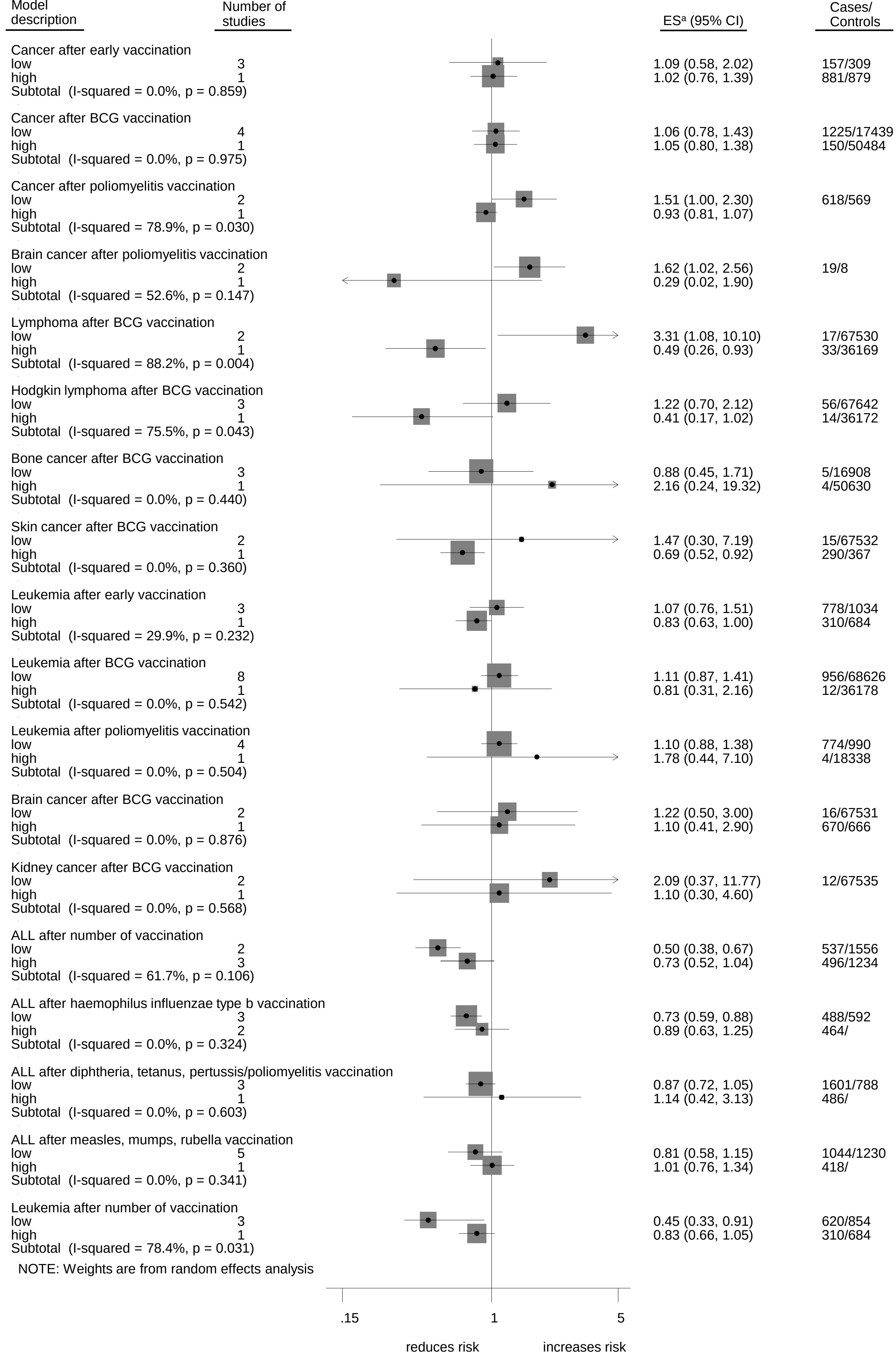

Supplementary Figure 1H. Vaccination and the risk of childhood cancer after stratification by study quality via the Newcastle-Ottawa Scale (low: below 6 points (fourth quintile of quality score of included studies); high: equal or above 6 points (fourth quintile of quality score of included studies)).

Abbreviations: ALL, acute lymphoblastic leukemia; BCG, Bacillus Calmette-Guérin; OR, odds ratio; ES, estimate.

[a] ES includes single-study odds ratios or hazard ratios and summary odds ratios.
