# Supplementary material for: Vaccination and the Risk of Childhood Cancer—A Systematic Review and Meta-Analysis
Source: Front Oncol. 2021 Jan 22;10:610843. doi: 10.3389/fonc.2020.610843 (PMC7862764; doi:10.3389/fonc.2020.610843)
Supplement: Supplementary file 5 [file DataSheet_5.pdf]

Author, year (reference)	Study design	Exposure assessment	Study years	Quality score	Latency period	ES[a] (95% CI)	Cases/Controls
Kaatsch, 1998 (92)[b]	case-control	self-report	1992-1994	21.6	no	0.94 (0.88, 0.99)	654/654
Dockerty, 1999 (93)[c]	case-control	records	1990-1993	24.2	yes	0.99 (0.85, 1.15)	118/287
Overall (I-squared = 0.0%, p = 0.537)						0.94 (0.89, 1.00)	

NOTE: Weights are from random effects analysis

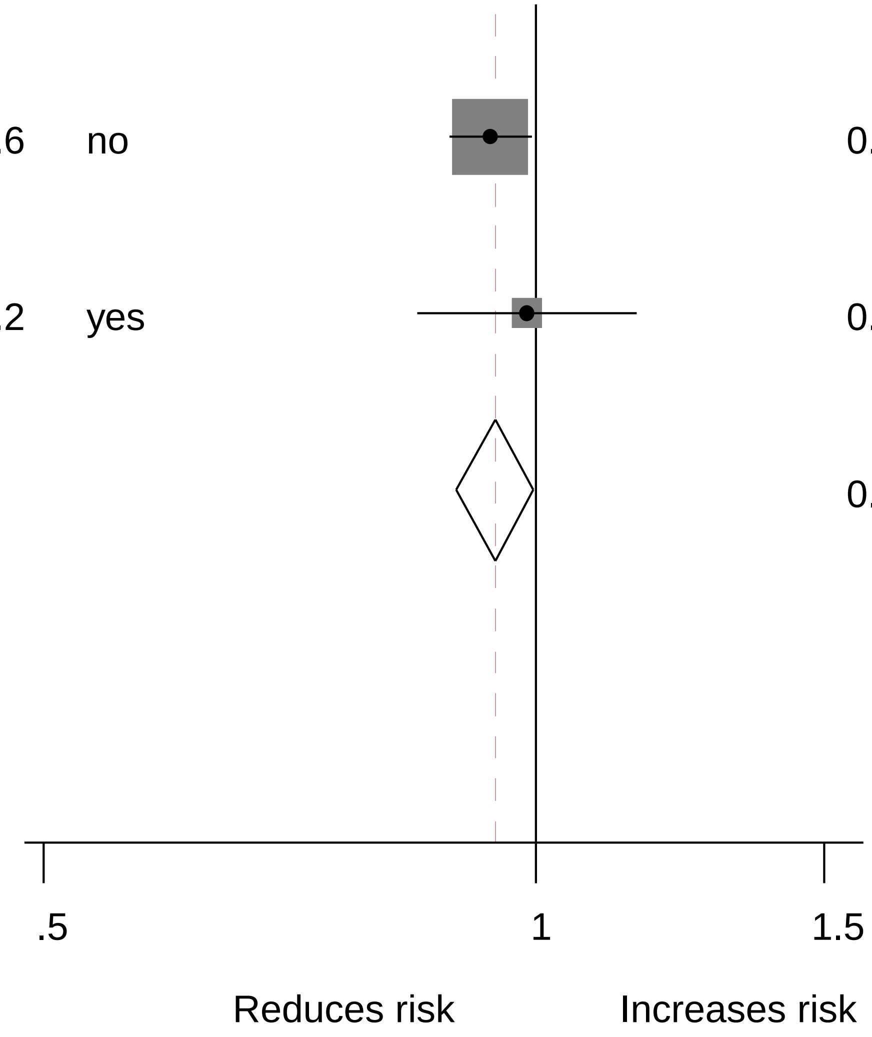

Supplementary Figure 2. Number of vaccination and the risk of leukemia (dose-response analysis for an increasing number of vaccinations).

Abbreviations: ES, estimate.

[a] ES includes estimates from trend analysis of single-studies and summary odds ratio.

[b] Estimate of trend analysis for >6 vs. 0-3 and 4-6 vs. 0-3 vaccine injections.

[c] Estimate of trend analysis for 1-2 vs. 0, 3-4 vs. 0, and 5+ vs. 0 vaccine injections.
