# Supplementary material for: Vaccination and the Risk of Childhood Cancer—A Systematic Review and Meta-Analysis
Source: Front Oncol. 2021 Jan 22;10:610843. doi: 10.3389/fonc.2020.610843 (PMC7862764; doi:10.3389/fonc.2020.610843)
Supplement: Supplementary file 6 [file DataSheet_6.pdf]

Supplementary Figure 3A. Publication bias: BCG vaccination and risk of leukemia

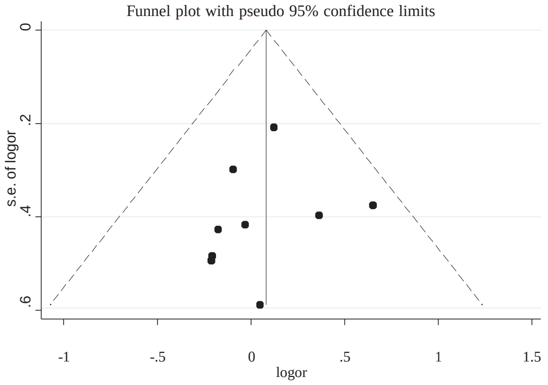

Supplementary Figure 3B. Publication bias: BCG vaccination and risk of leukemia

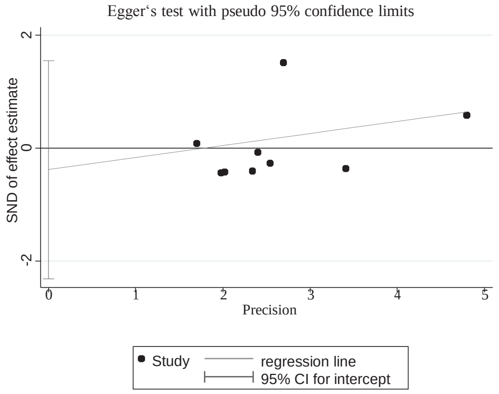

Supplementary Figure 3C. Publication bias: BCG vaccination and risk of leukemia

Egger's test with pseudo 95% confidence limits

Number of studies = 9 Root MSE = .7813

Std Eff	Coef.	Std. Err.	t	P>|t|	[95% Conf.	Interval]
slope	.2138512	.2929403	0.73	0.489	-.4788424	.9065449
bias	-.3865152	.8180817	-0.47	0.651	-2.320971	1.547941

Test of H0: no small-study effects P = 0.651

Supplementary Figure 3A-C. Publication bias: BCG vaccination and risk of leukemia.

Abbreviations: BCG, Bacillus Calmette–Guérin (vole bacillus; tuberculosis); CI, confidence interval; Coef, coefficient; Conf, confidence; Eff, effect; Err, error; logor, log odds ratio; MSE, mean square error; P, P-value; se; standard error; SND, standard normal deviate; Std, standard.

Supplementary Figure 4A. Publication bias: BCG vaccination and risk of cancer

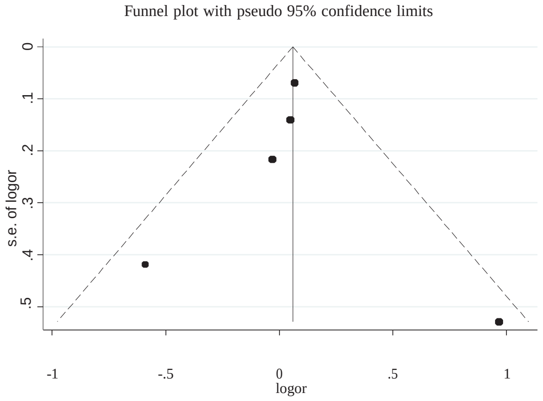

Supplementary Figure 4B. Publication bias: BCG vaccination and risk of cancer

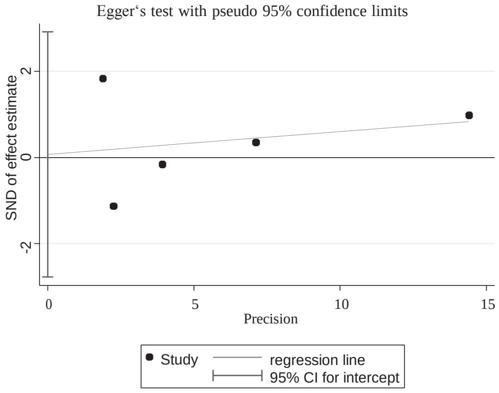

Supplementary Figure 4C. Publication bias: BCG vaccination and risk of cancer

Egger's test with pseudo 95% confidence limits

Number of studies = 5 Root MSE = 1.235

Std Eff	Coef.	Std. Err.	t	P>|t|	[95% Conf.	Interval]
slope	.0530243	.1190101	0.45	0.686	-.3257189	.4317675
bias	.0684937	.8942422	0.08	0.944	-2.777384	2.914371

Test of H0: no small-study effects P = 0.944

Supplementary Figure 4A-C. Publication bias: BCG vaccination and risk of cancer.

Abbreviations: BCG, Bacillus Calmette–Guérin (vole bacillus; tuberculosis); CI, confidence interval; Coef, coefficient; Conf, confidence; Eff, effect; Err, error; logor, log odds ratio; MSE, mean square error; P, P-value; se; standard error; SND, standard normal deviate; Std, standard.

Supplementary Figure 5A. Publication bias: Polio vaccination and risk of leukemia

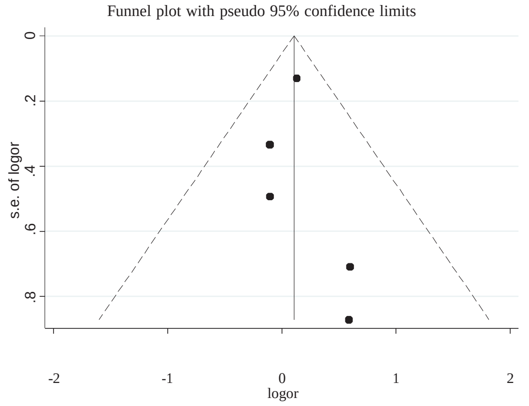

Supplementary Figure 5B. Publication bias: Polio vaccination and risk of leukemia

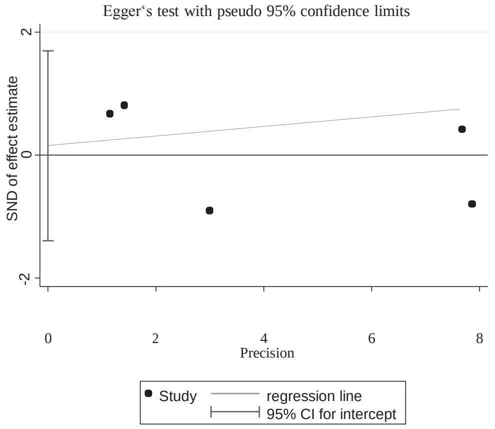

Supplementary Figure 5C. Publication bias: Polio vaccination and risk of leukemia

Egger's test with pseudo 95% confidence limits

Number of studies = 5 Root MSE = .6663

Std Eff	Coef.	Std. Err.	t	P>|t|	[95% Conf.	Interval]
slope	.0776011	.1252224	0.62	0.579	-.3209123	.4761146
bias	.1533261	.4849627	0.32	0.773	-1.390042	1.696694

Test of H0: no small-study effects P = 0.773

Supplementary Figure 5A-C. Publication bias: Polio vaccination and risk of leukemia.

Abbreviations: CI, confidence interval; Coef, coefficient; Conf, confidence; Eff, effect; Err, error; logor, log odds ratio; MSE, mean square error; P, P-value; Polio, poliomyelitis; se; standard error; SND, standard normal deviate; Std, standard.

Supplementary Figure 6A. Publication bias: Hib vaccination and risk of ALL

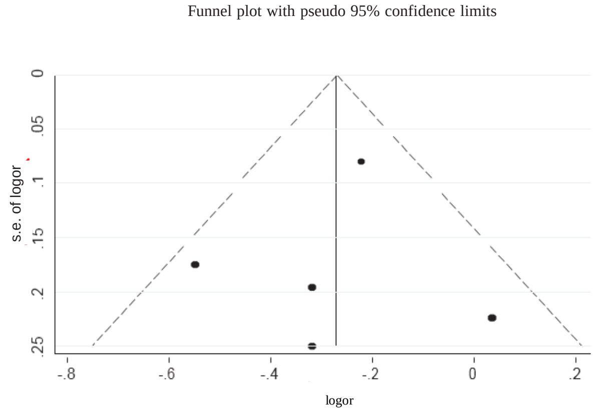

Supplementary Figure 6B. Publication bias: Hib vaccination and risk of ALL

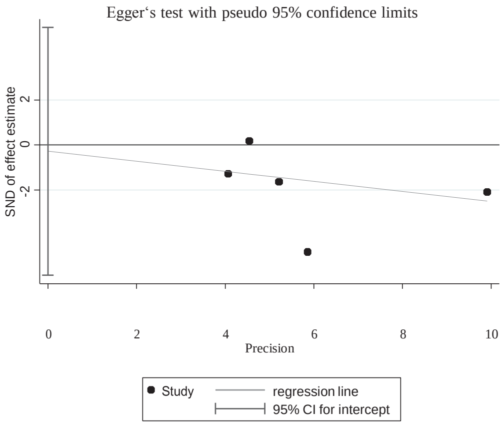

Supplementary Figure 6C. Publication bias: Hib vaccination and risk of ALL

Egger's test with pseudo 95% confidence limits

Number of studies = 5 Root MSE = 1.285

Std Eff	Coef.	Std. Err.	t	P>|t|	[95% Conf.	Interval]
slope	-.2232346	.2753133	-0.81	0.477	-1.099404	.6529351
bias	-.2830131	1.728541	-0.16	0.880	-5.784002	5.217976

Test of H0: no small-study effects P = 0.880

Supplementary Figure 6A-C. Publication bias: Hib vaccination and risk of ALL.

Abbreviations: ALL, Acute lymphoblastic leukemia; CI, confidence interval; Coef, coefficient; Conf, confidence; Eff, effect; Err, error; Hib, Haemophilus influenzae type b; logor, log odds ratio; MSE, mean square error; P, P-value; se; standard error; SND, standard normal deviate; Std, standard.

Supplementary Figure 7A. Publication bias: MMR vaccination and risk of ALL

Funnel plot with pseudo 95% confidence limits

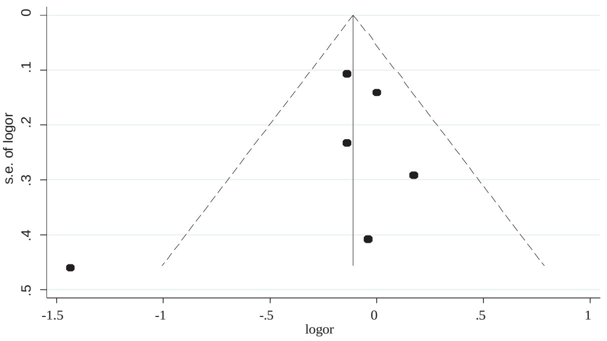

Supplementary Figure 7B. Publication bias: MMR vaccination and risk of ALL

Egger's test with pseudo 95% confidence limits

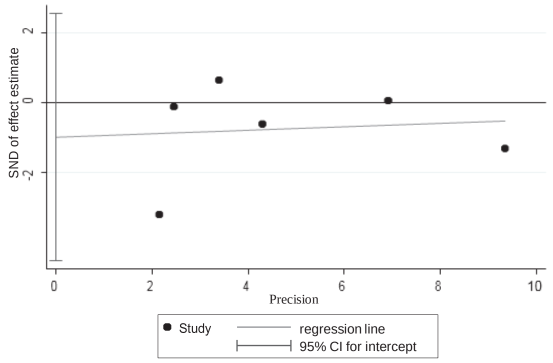

Supplementary Figure 7C. Publication bias: MMR vaccination and risk of ALL

Egger's test with pseudo 95% confidence limits

Number of studies = 6 RootMSE = 1.48

Std_Eff	Coef.	Std. Err.	t	P>|t|	[95% Conf. Interval]	
slope	.0498346	.2352268	0.21	0.843	-.6032597	.7029289
bias	-.9804296	1.274464	-0.77	0.485	-4.518909	2.55805

Test of H0: no small-study effects P = 0.485

Supplementary Figure 7A-C. Publication bias: MMR vaccination and risk of ALL.

Abbreviations: ALL, Acute lymphoblastic leukemia; CI, confidence interval; Coef, coefficient; Conf, confidence; Eff, effect; Err, error; logor, log odds ratio; MMR, Mumps-Measles-Rubella; MSE, mean square error; P, P-value; se; standard error; SND, standard normal deviate; Std, standard.

Supplementary Figure 8A. Publication bias: Number of vaccinations and risk of ALL

Funnel plot with pseudo 95% confidence limits

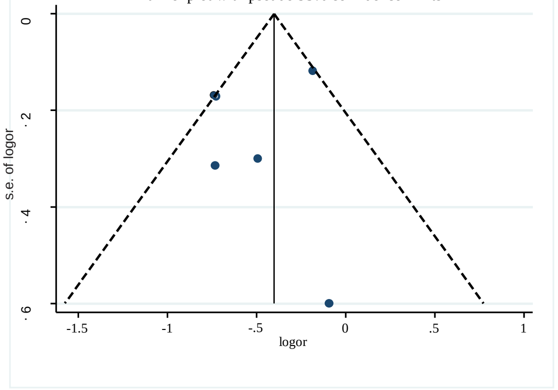

Supplementary Figure 8B. Publication bias: Number of vaccinations and risk of ALL

Egger's test with pseudo 95% confidence limits

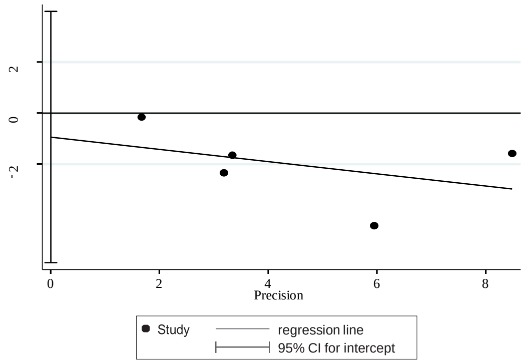

Supplementary Figure 8C. Publication bias: Number of vaccinations and risk of ALL

Egger's test with pseudo 95% confidence limits

Number of studies = 5 RootMSE = 1.627

Std Eff	Coef.	Std. Err.	t	P>|t|	[95% Conf. Interval]	
slope	-.2406651	.3017854	-0.80	0.483	-1.201081	.7197509
bias	-.9387165	1.547196	-0.61	0.587	-5.862586	3.985153

Test of H0: no small-study effects P = 0.587

Supplementary Figure 8A-C. Publication bias: Number of vaccinations and risk of ALL.

Abbreviations: ALL, Acute lymphoblastic leukemia; CI, confidence interval; Coef, coefficient; Conf, confidence; Eff, effect; Err, error; logor, log odds ratio; MSE, mean square error; P, P-value; se; standard error; SND, standard normal deviate; Std, standard.
